# Development of 7-hydroxy-4-methylcoumarin–cyanoacrylate hybrids with antiproliferative activity against breast cancer cells: from molecular design to *in vivo* validation

**DOI:** 10.1039/d6ra06101j

**Published:** 2026-10-27

**Authors:** Manar A. El-Zend, Ibrahim M. El-Deen, Zeinab Abdelkarim, Ibrahim M. Alanazi, Samar Zuhair Alshawwa, Essa M. Saied

**Affiliations:** a Department of Chemistry, Faculty of Science, Port Said University Port Said Egypt m.elzend@sci.psu.edu.eg ieldeen@yahoo.com Zeinab.elsayed@sci.psu.edu.eg; b Department of Pharmacology and Toxicology, Faculty of Medicine, Umm Al-Qura University Makkah 21955 Saudi Arabia imanzi@uqu.edu.sa; c Department of Pharmaceutical Sciences, College of Pharmacy, Princess Nourah bint Abdulrahman University P.O. Box 84428 Riyadh 11671 Saudi Arabia SZAlshawwa@pnu.edu.sa; d Chemistry Department, Faculty of Science, Suez Canal University Ismailia 41522 Egypt essa.saied@science.suez.edu.eg; e Institute for Chemistry, Humboldt Universität zu Berlin 12489 Berlin Germany

## Abstract

In the present study, a series of novel 6,8-dibromo-7-hydroxy-4-methylcoumarin–cyanoacrylate hybrids and their corresponding pyrazole derivatives were designed, synthesized, and evaluated for antiproliferative activity against breast cancer cells. Among the tested compounds, cyanoacrylate derivative 8 emerged as the most active analogue against MCF-7 cells (IC_50_ = 5.9 ± 0.4 µM) and retained antiproliferative activity against MDA-MB-231 cells (IC_50_ = 11.7 ± 0.4 µM). Compound 8 exhibited weaker cytotoxicity toward the non-cancerous MCF-10A and WI-38 cell lines (IC_50_ = 46 ± 3 and 37.4 ± 1.3 µM, respectively), although the magnitude of selectivity was cell-context dependent. Mechanistic investigations in MCF-7 cells revealed pronounced G2/M cell-cycle arrest (7.57% to 32.24%), inhibition of tubulin polymerization (IC_50_ = 4.7 ± 0.2 µM), and EGFR kinase activity (IC_50_ = 50 ± 3 nM). Flow-cytometric analysis showed an increase in total apoptotic cells from 2.71% to 33.19%, while LDH release increased 2.88-fold, indicating concomitant loss of membrane integrity. qRT-PCR demonstrated upregulation of BAX (7.9-fold), caspase-9 (8.8-fold), and p53 (4.7-fold) and downregulation of BCL-2 (0.27-fold), with corresponding protein-level changes confirmed by ELISA, providing complementary evidence for activation of the intrinsic apoptotic pathway. Molecular docking provided structural hypotheses for potential interactions of compound 8 within the tubulin colchicine-binding region and EGFR ATP-binding site. *In vivo*, compound 8 significantly suppressed tumor burden in the Ehrlich ascites carcinoma (EAC) mouse model, reducing viable tumor-cell counts by up to 72.4% and ascitic tumor volume by 85.3%. Treatment also ameliorated tumor-associated hepatic and renal dysfunction, oxidative stress, and inflammatory alterations, with histopathological and immunohistochemical findings supporting tissue recovery. Collectively, these findings identify compound 8 as a promising antiproliferative and antitumor lead and support further optimization and evaluation of this scaffold in more clinically relevant breast cancer models.

## Abstract 2

In the present study, a series of novel 6,8-dibromo-7-hydroxy-4-methylcoumarin–cyanoacrylate hybrids and their corresponding pyrazole derivatives were designed, synthesized, and evaluated for antiproliferative activity against breast cancer cells. Image created with the assistance of FigureLabs.

## Graphical abstract

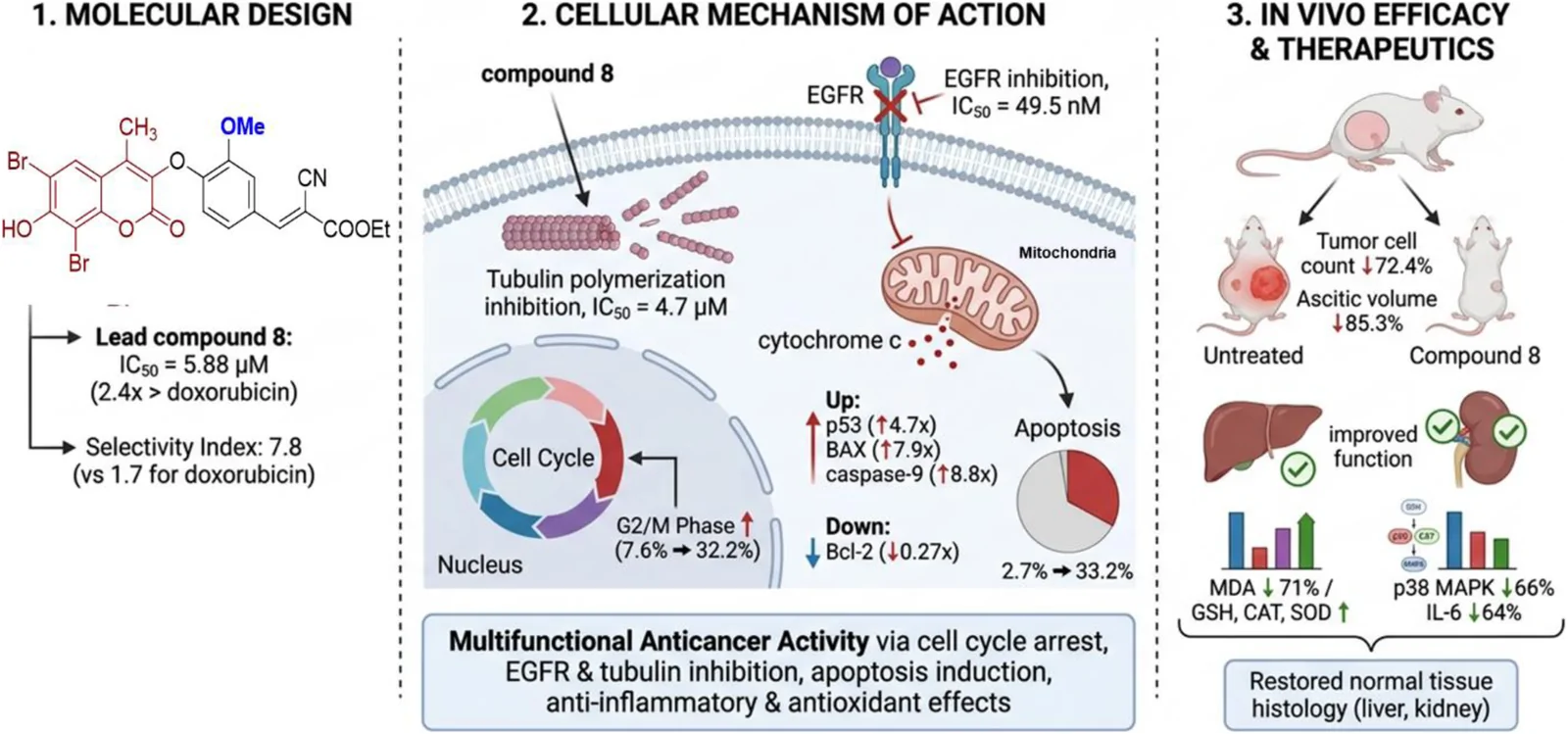

## 1. Introduction

Cancer chemotherapy has benefited substantially from the introduction of molecularly targeted therapies and precision medicine approaches. Nevertheless, the long-term clinical efficacy of many anticancer agents remains limited by the emergence of multidrug resistance, target mutations, activation of compensatory signaling pathways, and dose-limiting toxicities.^1^ In breast cancer, resistance frequently arises through complex molecular adaptations involving receptor tyrosine kinase signaling, PI3K/Akt/mTOR and MAPK pathways, and dynamic interactions with the tumor microenvironment.^2,3^ Among the molecular pathways implicated in breast cancer progression, dysregulation of cell-cycle progression, apoptosis, and growth factor signaling represent key hallmarks of malignancy. Microtubules are essential for mitotic spindle formation, and their disruption remains one of the most successful anticancer strategies, with agents such as paclitaxel, vinblastine, and combretastatin analogues inducing G2/M arrest and apoptotic cell death through inhibition of tubulin dynamics.^4–6^ Likewise, aberrant activation of epidermal growth factor receptor (EGFR) signaling promotes uncontrolled proliferation, invasion, metastasis, and therapeutic resistance, establishing EGFR as a validated target for anticancer drug development.^7^ In parallel, apoptosis-regulating proteins including p53, BAX, BCL-2, and caspase-9 play central roles in determining cancer cell survival and are widely utilized as mechanistic biomarkers of anticancer activity.^8^ In addition to proliferative signaling, oxidative stress and chronic inflammation are increasingly recognized as critical contributors to tumor progression. Excessive reactive oxygen species (ROS) generation promotes genomic instability and cancer development, while inflammatory mediators such as interleukin-6 (IL-6) and p38 MAPK support the establishment of a pro-tumorigenic microenvironment.^9,10^ Such biological complexity highlights the limitations of single-target therapeutic strategies^11,12^ and has stimulated growing interest in the rational design of structurally novel entities that combine multiple pharmacological features within a single framework capable of simultaneously modulating several cancer-associated pathways and reducing susceptibility to therapeutic resistance.^13,14^

Among the diverse heterocyclic frameworks explored in anticancer drug discovery, the coumarin nucleus has attracted sustained interest owing to its exceptional structural adaptability and ability to engage multiple cancer-relevant molecular targets. Mechanistic investigations have revealed that coumarin-based compounds can interfere with several hallmarks of cancer through inhibition of receptor tyrosine kinases, suppression of angiogenesis, modulation of PI3K/Akt/mTOR signaling, disruption of microtubule dynamics, inhibition of aromatase activity, and activation of mitochondrial apoptotic pathways.^15–17^ The growing interest in coumarins as potential anticancer scaffolds has generated a wide spectrum of structurally diverse derivatives with promising antiproliferative activity against breast cancer cells (Fig. 1). Among the earliest examples, umbelliferone (7-hydroxycoumarin) demonstrated notable antiproliferative activity against MDA-MB-231 and MCF-7 breast cancer cells (IC_50_ = 15.56 and 10.31 µM, respectively), accompanied by induction of G2/M cell-cycle arrest, highlighting the antiproliferative potential of the hydroxylated coumarin scaffold.^18^ Similarly, 4-methylumbelliferone suppressed breast cancer cell proliferation through inhibition of hyaluronan biosynthesis, further validating the biological relevance of the 7-hydroxy-4-methylcoumarin framework.^19^ Subsequent structural optimization led to the development of pharmacologically advanced analogues such as 4-methylcoumarin-7-*O*-sulfamate (COUMATE), a potent steroid sulfatase inhibitor capable of suppressing estrogen-dependent breast cancer growth *in vitro* and *in vivo*.^20^ Beyond the parent scaffold, extensive efforts have focused on exploiting molecular hybridization to enhance antiproliferative potency and introduce additional mechanisms of action. For example, coumarin–thiazolidinone hybrids demonstrated significant *in vitro* cytotoxicity against MCF-7 cells, with compound I emerging as the most active analogue and showing favorable interactions with the EGFR kinase domain, emphasizing the utility of coumarin hybridization for the development of multitarget antiproliferative compounds.^21^ Similar findings were reported for coumarin–chalcone hybrids, where compound II displayed potent *in vitro* antiproliferative activity against MCF-7 cells (IC_50_ = 22.4 µM), while compound III exhibited even greater potency (IC_50_ = 6.44 µM), highlighting the beneficial contribution of α,β-unsaturated pharmacophores to antiproliferative activity.^22,23^

**Fig. 1:**
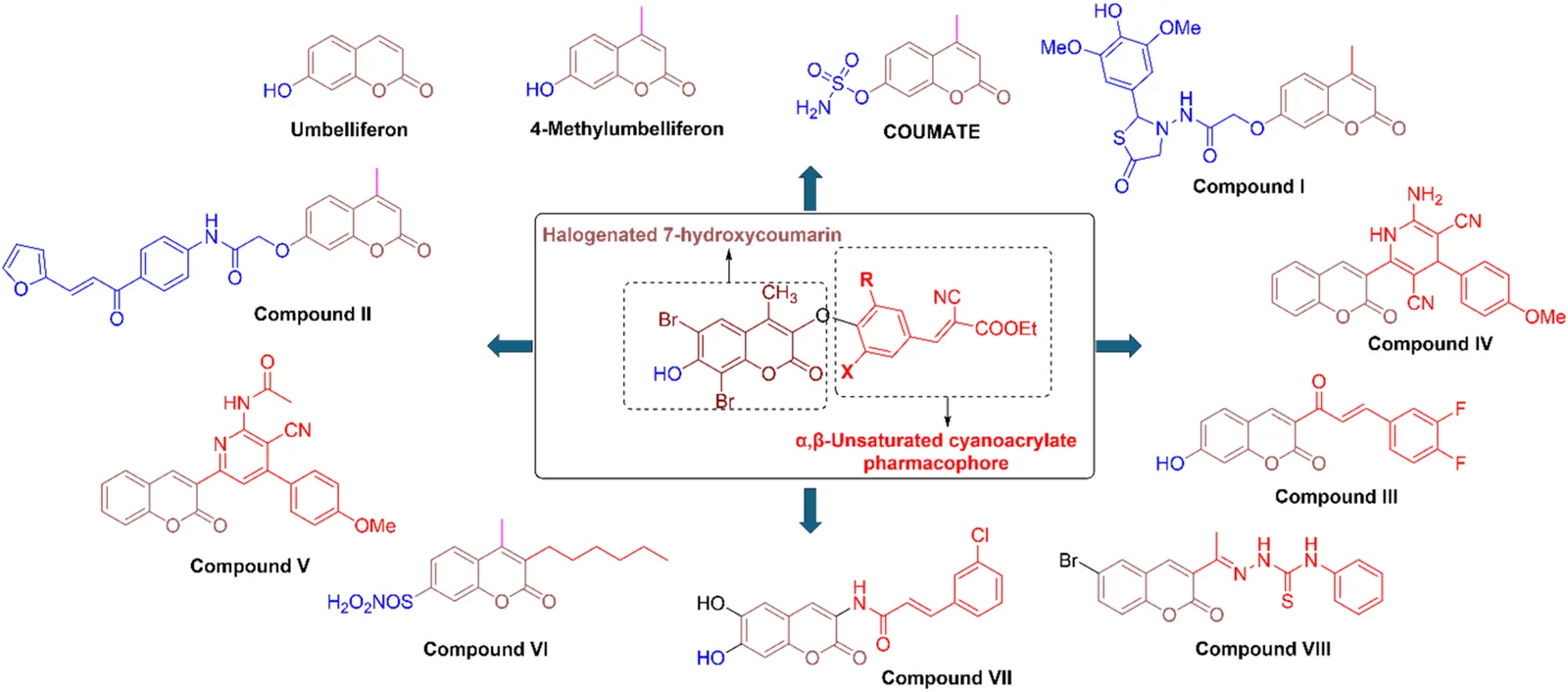
Literature-guided design rationale for the developed 6,8-dibromo-7-hydroxy-4-methylcoumarin hybrids. Representative coumarin derivatives with reported antiproliferative activity illustrate the structural and medicinal-chemistry strategies underlying the present design, including modification of the 7-hydroxy-4-methylcoumarin scaffold, strategic 6,8-dibromination, C-3/C-7 molecular hybridization, and incorporation of an α,β-unsaturated cyanoacrylate pharmacophore.

Functionalization at the C-3 position has emerged as one of the most successful medicinal chemistry strategies for coumarin optimization. Representative examples include benzopyran-2-one derivative IV, which exhibited nanomolar *in vitro* antiproliferative activity against MCF-7 cells (IC_50_ = 0.039 µM), and coumarin derivative V, which induced G2/M arrest and caspase-3 activation with an IC_50_ value of 1.11 µM.^24,25^ Likewise, bicyclic coumarin sulfamate VI displayed submicromolar *in vitro* antiproliferative activity against MCF-7 cells (IC_50_ = 0.68 µM), while the scopoletin–cinnamic hybrid VII demonstrated potent *in vitro* antiproliferative activity against MCF-7 cells (IC_50_ = 0.231 µM), emphasizing the versatility of C-3 modification for generating highly active breast cancer inhibitors.^26,27^ Another structural feature that has received increasing attention in coumarin-based drug design is halogen substitution. Brominated coumarins frequently exhibit superior *in vitro* antiproliferative activity. In support of this concept, Sarhan and co-workers reported a brominated coumarin derivative VIII bearing a thiosemicarbazone pharmacophore that displayed remarkable *in vitro* cytotoxic activity against MCF-7 cells (IC_50_ = 0.0314 µM). Structure–activity relationship studies revealed that the brominated coumarin nucleus contributed substantially to the observed potency, while the ethyl hydrazone linker was identified as the optimal spacer for activity.^28^ Collectively, these findings indicate that the 7-hydroxy-4-methylcoumarin scaffold represents a promising pharmacophore for the development of antiproliferative agents, while C-3 functionalization, molecular hybridization, and strategic bromination constitute effective approaches for optimizing biological activity.

Based on these considerations, and in continuation of our efforts toward the development of biologically active hybrids,^29–34^ a set of novel 6,8-dibromo-7-hydroxy-4-methylcoumarin–cyanoacrylate conjugates and their corresponding pyrazole derivatives were designed and synthesized. The antiproliferative activity of the synthesized compounds was initially evaluated against MCF-7 human breast cancer cells, followed by expanded profiling of the lead compound against MDA-MB-231 breast cancer cells and the non-cancerous MCF-10A and WI-38 cell lines. Mechanistic investigations of the lead analogue were subsequently performed in MCF-7 cells, including cell-cycle analysis, apoptosis assessment, tubulin polymerization inhibition, EGFR inhibitory activity, molecular docking studies, and expression analysis of apoptosis-related markers. Furthermore, the *in vivo* antitumor efficacy of the lead compound was investigated in an Ehrlich ascites carcinoma (EAC) mouse model through evaluation of tumor burden, histopathological alterations, oxidative stress biomarkers, and inflammatory mediators to elucidate its potential mechanism of action.

## 2. Results and discussion

### 2.1 Chemistry

The synthesis of the envisioned 6,8-dibromo-7-hydroxy-4-methylcoumarin hybrid derivatives was accomplished through the synthetic pathway shown in Scheme 1, starting from commercially available resorcinol (1) and substituted 4-hydroxybenzaldehydes (4a, b). Initially, condensation of resorcinol (1) with ethyl acetoacetate under acidic conditions afforded 7-hydroxy-4-methylcoumarin (2) in 89% yield.^35^ Subsequent bromination of compound 2 using *N*-bromosuccinimide (NBS) in the presence of catalytic benzoyl peroxide furnished the key intermediate 6,8-dibromo-7-hydroxy-4-methylcoumarin (3) in 76% yield.^36^ In parallel, the substituted ethyl (*E*)-2-cyano-3-(4-hydroxyphenyl)acrylate intermediates were synthesized from the corresponding 4-hydroxybenzaldehydes (4a, b). Bromination of aldehydes 4a, b with bromine in glacial acetic acid yielded the corresponding brominated derivatives 5a, b in 59–71% yields. Subsequently, Knoevenagel condensation of aldehydes 4a, b and 5a, b with ethyl cyanoacetate in the presence of thiourea and catalytic ammonium chloride afforded the corresponding α,β-unsaturated cyanoacrylate derivatives 6a–d in good yields ranging from 66% to 83%.^37^ With both key building blocks in hand, coupling of the coumarin intermediate 3 with cyanoacrylate derivatives 6a–d under basic conditions furnished the desired coumarin–cyanoacrylate hybrids 7, 8, 11, and 12 in good yields (55–79%). To investigate the influence of cyclization of the α,β-unsaturated cyanoacrylate pharmacophore on biological activity, compounds 7 and 8 were further reacted with hydrazine hydrate to generate the corresponding pyrazole-carbonitrile derivatives 9 and 10 in 82% and 76% yields, respectively. The structures of all synthesized compounds were confirmed by FTIR, elemental microanalysis, and ^1^H, ^13^C-NMR spectroscopy, and the obtained spectroscopic data were fully consistent with the proposed structures.

**Scheme 1:**
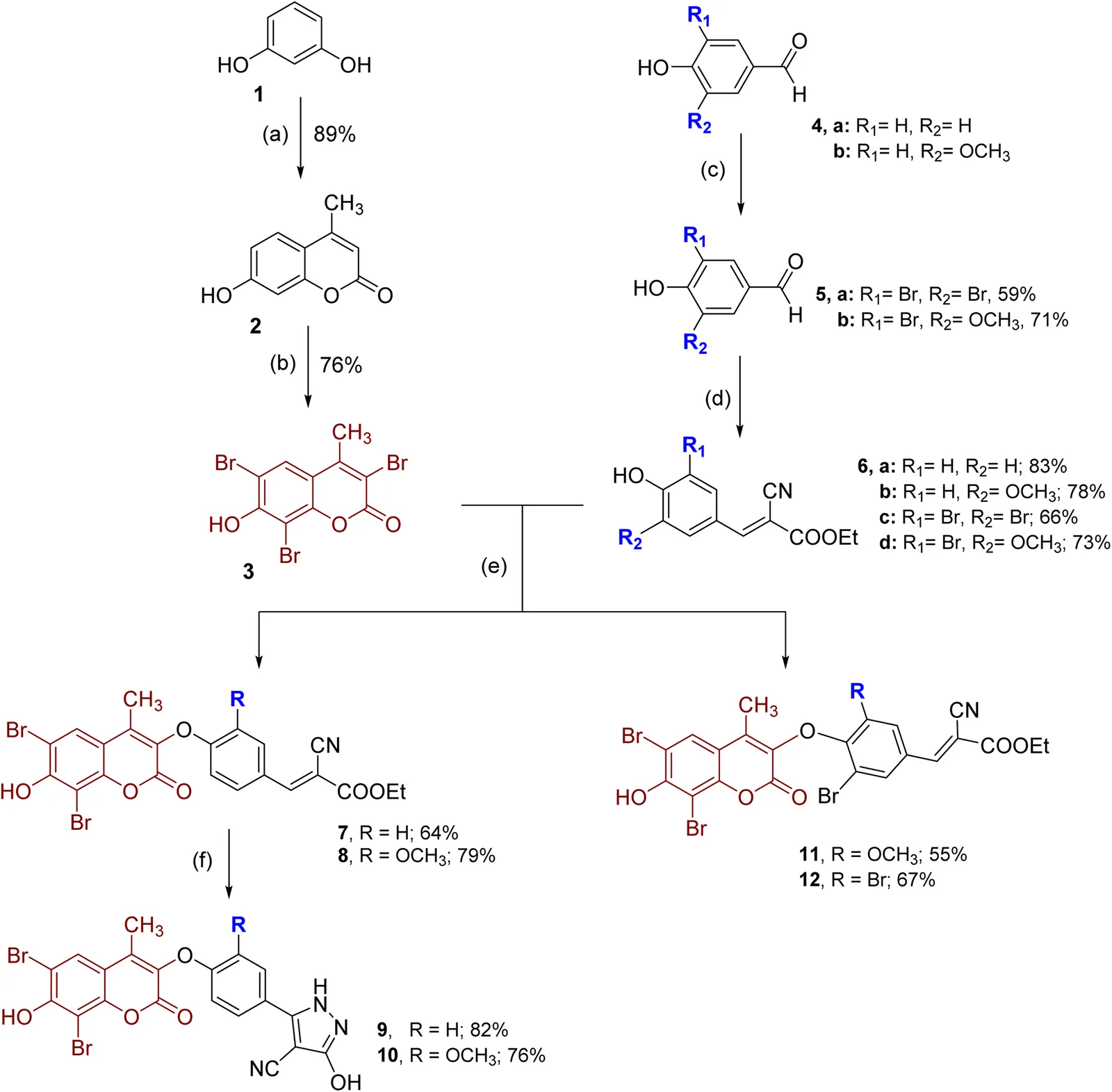
Synthesis of 6,8-dibromo-7-hydroxy-4-methylcoumarin hybrid derivatives (7–12). Reagents and conditions: (a) ethyl acetoacetate, 80% H_2_SO_4_, 85 °C, 12 h; (b) NBS, benzoyl peroxide (cat.), benzene, reflux, 16 h; (c) 4a–b, bromine, glacial AcOH, reflux. 8–12 h; (d) 4a–b/5a–b, ethyl cyanoacetate, thiourea, NH_4_Cl (cat.), THF, 70 °C, 8–12 h; (e) (i) 6a–d, Cs_2_CO_3_, EtOH, 2–4 h; (ii) 3, 78 °C, 16–24 h; (f) 7/8, NH_2_NH_2_.hyd., EtOH, 70 °C, 16 h.

### 2.2 *In vitro* biological assessments

#### 2.2.1 Antiproliferative activity against MCF-7 breast cancer cells

The antiproliferative activity of the synthesized 6,8-dibromo-7-hydroxy-4-methylcoumarin hybrids was evaluated against MCF-7 breast cancer cells, using doxorubicin as a reference drug (Fig. 2). The tested compounds displayed variable cytotoxic activity, with IC_50_ values ranging from 5.9 ± 0.4 to 50 ± 3 µM. Among the series, compound 8 emerged as the most active derivative, exhibiting an IC_50_ value of 5.9 ± 0.4 µM, which was approximately 2.4-fold more potent than doxorubicin (IC_50_ = 14.2 ± 0.6 µM). Compounds 10 and 11 also showed promising activity, with IC_50_ values of 11.6 ± 0.6 and 13.1 ± 0.8 µM, respectively, both slightly superior to doxorubicin. Compound 7 showed moderate activity (IC_50_ = 16.2 ± 1 µM), whereas compounds 9 and 12 were considerably less active, with IC_50_ values of 37 ± 2 and 50 ± 3 µM, respectively. Preliminary SAR analysis revealed that both the nature and position of substituents on the aryl-cyanoacrylate moiety markedly influenced antiproliferative activity. Compound 7, bearing an unsubstituted 4-hydroxyphenyl-cyanoacrylate fragment, displayed moderate activity against MCF-7 cells (IC_50_ = 16.2 ± 1 µM). Introduction of a methoxy group at the 3-position afforded compound 8, which emerged as the most potent derivative of the series (IC_50_ = 5.9 ± 0.4 µM), corresponding to an approximately 2.8-fold improvement relative to compound 7. Within this limited series, these findings identify the 3-methoxy-4-hydroxy substitution pattern as a potentially favorable structural feature for antiproliferative activity. However, evaluation of a broader set of analogues will be required to establish the generality of this preliminary SAR trend. To further explore the influence of halogen substitution, compound 8 was modified by introducing a bromine atom at the 3-position while retaining the 5-methoxy and 4-hydroxy substituents, yielding compound 11. Although compound 11 maintained good activity (IC_50_ = 13.1 ± 0.8 µM), its potency was approximately two-fold lower than that of compound 8. This reduction indicates that incorporation of the bulky electron-withdrawing bromine atom partially compromises the favorable contribution of the methoxy-substituted aromatic ring, possibly due to increased steric demand or altered electronic distribution. A more pronounced decline in activity was observed for compound 12 (IC_50_ = 50 ± 3 µM), in which the methoxy group of compound 11 was replaced by a second bromine atom to generate the 3,5-dibromo-4-hydroxy analogue. The dramatic loss of potency observed for compound 12 suggests that excessive halogenation of the aromatic ring is detrimental to antiproliferative activity. This may arise from increased steric bulk, reduced hydrogen-bonding capability, and unfavorable electronic effects that collectively impair productive target interactions.

**Fig. 2:**
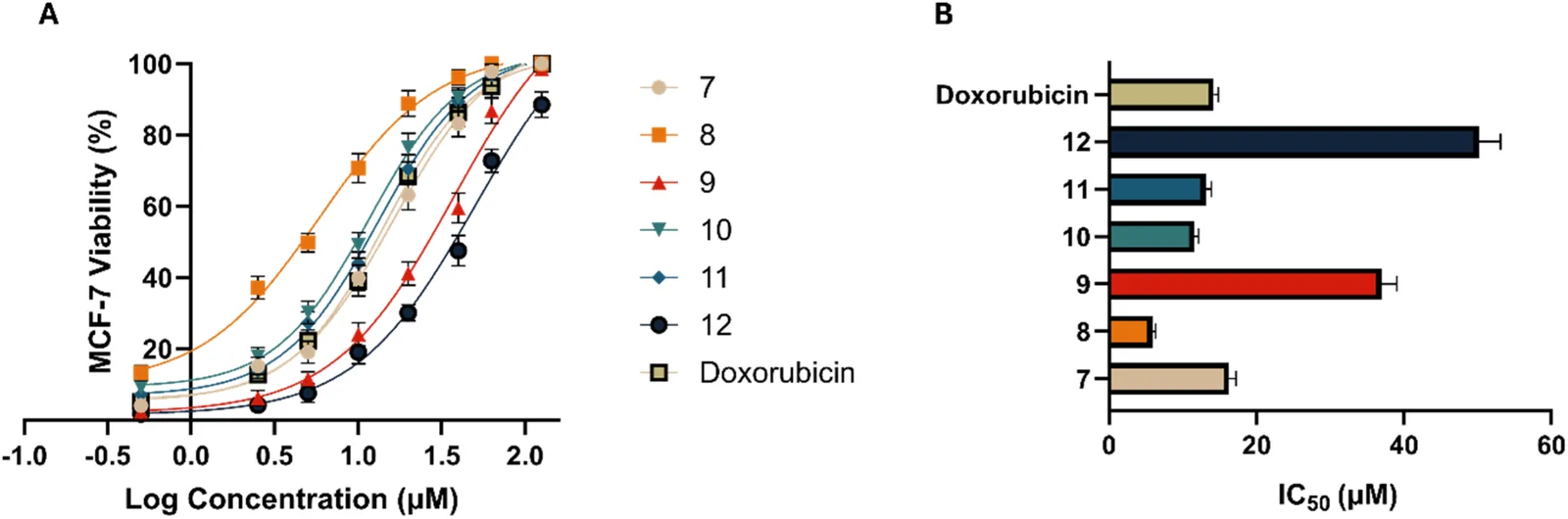
Antiproliferative activity of the synthesized coumarin-based hybrids against MCF-7 breast cancer cells. (A) Representative dose–response curves of compounds 7–12 and doxorubicin following treatment of MCF-7 cells. IC_50_ values were calculated by nonlinear regression analysis using a log(inhibitor) *versus* normalized response (variable slope) model. (B) Cellular IC_50_ values, defined as the concentrations producing a 50% reduction in MTT-derived cell viability relative to untreated controls, were calculated by nonlinear regression using a log(inhibitor) *versus* normalized response (variable slope) model. Data are presented as mean ± SD from three independent experiments (*n* = 3).

The effect of cyclization of the α,β-unsaturated cyanoacrylate linker was also investigated. Conversion of compound 7 into the corresponding pyrazole-carbonitrile derivative 9 resulted in a substantial reduction in activity (IC_50_ = 37 ± 2 µM). Likewise, cyclization of the most potent analogue 8 yielded compound 10, which retained good activity (IC_50_ = 11.6 ± 0.6 µM) but remained approximately two-fold less active than its open-chain precursor. These observations indicate that the α,β-unsaturated cyanoacrylate scaffold is generally more favorable for antiproliferative activity than the corresponding pyrazoline ring system. The superior performance of the open-chain analogues may be associated with differences in conformational flexibility and π-conjugation; however, a broader analogue series will be required to establish the structural basis of this trend. Overall, these preliminary SAR observations identify compound 8 as possessing the most favorable combination of structural features among the six derivatives examined. Nevertheless, the limited size of the present compound set precludes establishment of a comprehensive SAR, and the observed effects of aryl substitution, bromination, and linker cyclization should therefore be regarded as initial structural trends requiring validation across a broader analogue series. To broaden the biological evaluation of compound 8 and assess whether its antiproliferative activity extended beyond MCF-7 cells, the lead compound was additionally evaluated against the triple-negative breast cancer cell line MDA-MB-231. Its selectivity profile was also expanded by including the non-cancerous human lung fibroblast cell line WI-38 in addition to the non-tumorigenic breast epithelial MCF-10A cells. All cellular responses were determined after 48 h using the MTT assay, with doxorubicin evaluated under the same experimental conditions (Fig. S1). Compound 8 retained substantial antiproliferative activity against MDA-MB-231 cells, exhibiting an IC_50_ value of 11.7 ± 0.4 µM, compared with 5.9 ± 0.4 µM against MCF-7 cells. Doxorubicin was more potent against MDA-MB-231 cells, with an IC_50_ of 2 ± 0.1 µM. Importantly, compound 8 showed considerably weaker effects toward both non-cancerous cell lines, with IC_50_ values of 46 ± 3 µM in MCF-10A cells and 37.4 ± 1.3 µM in WI-38 cells. The corresponding values for doxorubicin were 24 ± 2 and 15.8 ± 0.6 µM, respectively. Assessment of the resulting selectivity indices (SI) further indicated that the selectivity profile depended on the cancer-cell background. Relative to MCF-7 cells, compound 8 displayed SI values of 7.81 and 6.34 using MCF-10A and WI-38 cells, respectively, compared with 1.70 and 1.11 for doxorubicin. Against MDA-MB-231 cells, compound 8 retained selectivity windows of 3.93 and 3.20 relative to MCF-10A and WI-38 cells, respectively. On the other hand, doxorubicin showed higher corresponding SI values of 12.12 and 7.98, reflecting its markedly greater potency toward MDA-MB-231 cells (Table S1). Thus, the expanded profiling supports that compound 8 possesses antiproliferative activity in two biologically distinct breast cancer cell lines while exhibiting lower absolute cytotoxicity toward two non-cancerous cell types. At the same time, the differential SI profiles indicate that selectivity is cell-context dependent rather than an intrinsic fixed property of the compound. These findings support the selection of compound 8 as the lead analogue for further mechanistic and *in vivo* investigation.

#### 2.2.2 Compound 8 induces G2/M phase arrest in MCF-7 cells through inhibition of tubulin polymerization

Following the potent antiproliferative activity exhibited by compound 8 against MCF-7 breast cancer cells (IC_50_ = 5.9 ± 0.4 µM), its effect on cell-cycle progression was investigated to gain mechanistic insight into the basis of growth inhibition. As shown in Fig. 3, treatment of MCF-7 cells with compound 8 markedly altered cell-cycle distribution, resulting in a substantial accumulation of cells at the G2/M phase (32.24%) compared with untreated control cells (7.57%). This effect was accompanied by a reduction in both G0/G1 and S phase populations, which decreased from 71.35% and 21.08% in control cells to 54.01% and 13.75%, respectively. The pronounced enrichment of the G2/M population indicates that compound 8 effectively interrupts mitotic progression, thereby preventing completion of cell division and suppressing cellular proliferation. Consequently, the possibility that compound 8 interferes with microtubule organization was investigated through an *in vitro* tubulin polymerization assay. Consistent with the cell-cycle findings, compound 8 exhibited considerable inhibition of tubulin polymerization with an IC_50_ value of 4.7 ± 0.2 µM, compared with 2.8 ± 0.1 µM for the reference colchicine-site inhibitor combretastatin A4 (CA4) (Fig. 3). Although compound 8 was less potent than CA4, its pronounced inhibitory activity indicates a clear ability to interfere with microtubule assembly. The close agreement between the cellular and biochemical findings suggests that the observed G2/M arrest is mechanistically linked to disruption of microtubule dynamics rather than representing a nonspecific consequence of cytotoxic stress. Similar correlations between tubulin disruption, mitotic arrest, and suppression of cancer cell proliferation have been extensively reported for clinically relevant microtubule-targeting agents.^38,39^

**Fig. 3:**
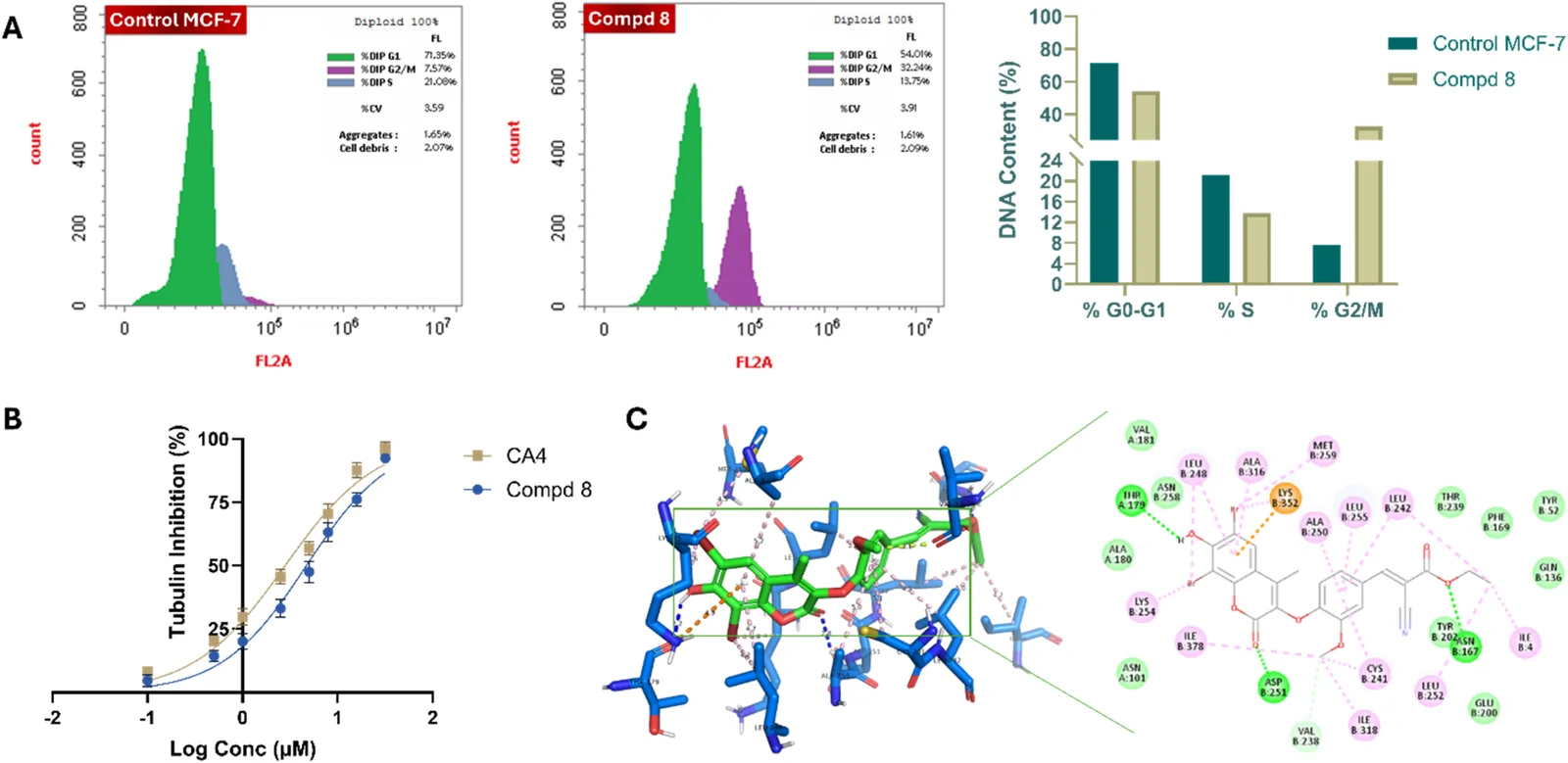
Mechanistic evaluation of compound 8 as a tubulin-targeting antiproliferative agent. (A) Representative flow cytometric analysis of cell-cycle progression in MCF-7 cells. The panel shows DNA-content histograms of untreated control cells and MCF-7 cells treated with compound 8 (5.9 µM) for 24 h, together with the quantitative distribution of cells in the G0/G1, S, and G2/M phases. (B) Concentration-dependent inhibition of tubulin polymerization by compound 8 and the reference inhibitor combretastatin A4 (CA4). Data are presented as mean ± SD from three independent experiments (*n* = 3). (C) Predicted binding mode of compound 8 within the colchicine-binding pocket of tubulin (PDB: 5LYJ), showing both the three-dimensional ligand orientation and the corresponding two-dimensional protein–ligand interaction map.

To further explore the molecular basis of tubulin inhibition by compound 8, docking studies were performed within the colchicine-binding site of tubulin 40. Prior to docking, the reliability of the protocol was validated through redocking of the co-crystallized ligand combretastatin A4, which successfully reproduced the experimental binding orientation with an RMSD value of 0.777 Å. Furthermore, the redocked pose displayed a binding affinity (−8.78 kcal mol^−1^) comparable to that of the native ligand (−8.41 kcal mol^−1^), confirming the suitability of the adopted docking methodology for subsequent analysis (Fig. S2). Docking simulations predicted that compound 8 could be accommodated within the colchicine-binding cavity and establish several contacts with residues lining the binding pocket (Fig. 3 and S4). In particular, compound 8 formed conventional hydrogen bonds with ASN167, ASP251, and THR179, together with hydrophobic interactions involving LEU242, LEU248, LEU255, MET259, ALA316, ILE318, ILE378, CYS241, and LYS352. Compound 8 also displayed a more favorable hydrogen-bond contribution (−2.17 kcal mol^−1^) than the co-crystallized ligand. However, these favorable local interactions should be interpreted in the context of the overall docking energetics. Compound 8 exhibited a substantially less favorable predicted binding affinity (−5.35 kcal mol^−1^) than redocked CA4 (−8.78 kcal mol^−1^), together with a pronounced steric repulsion penalty (+14.57 kcal mol^−1^) and increased intramolecular strain (+1.18 kcal mol^−1^). These unfavorable energetic contributions indicate suboptimal accommodation of the comparatively larger scaffold of compound 8 within the docking model, despite the predicted polar and hydrophobic contacts. Moreover, the moderate CNN score (0.645) does not provide strong evidence for a native-like binding pose (Tables S2–S4). Thus, the docking results should not be interpreted as establishing the colchicine-binding site as the definitive binding mode of compound 8, but rather as suggesting a possible interaction with this region. The predicted pose remains subject to the inherent limitations of the docking model, including the restricted treatment of protein flexibility and solvent-mediated effects, and further structural or dynamic studies would be required to establish the specific molecular binding mode. Importantly, the observed inhibition of tubulin polymerization provides independent functional evidence that compound 8 interferes with tubulin assembly, irrespective of the binding orientation predicted by docking. The docking analysis should be regarded as a tentative structural hypothesis concerning possible interaction with the colchicine-binding region rather than as a quantitative predictor of inhibitory potency. The comparatively less favorable score of compound 8 relative to CA4 is therefore interpreted as reflecting suboptimal accommodation of its larger molecular scaffold within the modeled colchicine-binding cavity, consistent with the observed steric-repulsion and conformational-strain penalties, rather than as evidence against the experimentally measured inhibition of tubulin polymerization. Collectively, the cellular and biochemical findings support disruption of tubulin polymerization as a contributor to the G2/M arrest induced by compound 8, while the docking analysis provides a tentative hypothesis regarding its possible interaction with the colchicine-binding region.

#### 2.2.3 Compound 8 triggers programmed cell death through suppression of EGFR signaling and activation of the intrinsic mitochondrial apoptotic pathway

Since prolonged mitotic arrest induced by microtubule-disrupting agents frequently culminates in apoptotic cell death,^41^ the ability of compound 8 to induce apoptosis in MCF-7 cells was subsequently investigated. As shown in Fig. 4, treatment with compound 8, at its IC_50_ concentration, resulted in a pronounced increase in apoptotic cell death compared with untreated cells. The total apoptotic population increased from 2.71% in the control group to 33.19% following treatment. This effect was primarily attributed to substantial elevations in both early apoptotic cells (0.37% *vs.* 18.44%) and late apoptotic cells (0.12% *vs.* 10.72%). A modest increase in necrotic cells was also observed (2.22% *vs.* 4.03%). The marked predominance of early and late apoptotic populations over the necrotic fraction indicates that apoptosis represents a major mode of cell death induced by compound 8. The observed induction of apoptosis is consistent with the preceding cell-cycle findings, where compound 8 caused pronounced G2/M phase arrest through inhibition of tubulin polymerization. To further elucidate the molecular mechanisms underlying its pro-apoptotic activity, the effect of compound 8 on epidermal growth factor receptor (EGFR) signaling was investigated. EGFR is a critical regulator of tumor cell proliferation and survival, and aberrant activation of this receptor promotes oncogenesis through stimulation of downstream PI3K/AKT and MAPK signaling cascades.^42,43^ As illustrated in Fig. 4B, compound 8 exhibited potent dose-dependent inhibition of EGFR kinase activity with an IC_50_ value of 50 ± 3 nM, compared with 19 ± 1 nM for the reference inhibitor erlotinib. Although less potent than erlotinib in the biochemical kinase assay, compound 8 demonstrated substantial EGFR inhibitory activity in the nanomolar range, indicating that EGFR inhibition may contribute to, but should not be considered solely responsible for, its cellular antiproliferative and pro-apoptotic effects. It is noteworthy that the biochemical EGFR inhibitory potency of compound 8 (IC_50_ = 50 ± 3 nM) was substantially greater than its cellular antiproliferative potency against MCF-7 cells (IC_50_ = 5.9 ± 0.4 µM). This difference is not unexpected because the two assays measure distinct biological endpoints under substantially different experimental conditions. The EGFR kinase assay directly exposes the purified enzyme to the test compound under a defined biochemical environment, whereas the cellular assay requires the compound to remain available in culture medium, traverse the cell membrane, achieve an adequate intracellular free concentration, and act within the complex cellular environment over the treatment period. Factors such as limited cellular permeability, nonspecific binding to serum or cellular components, intracellular sequestration, active efflux, or metabolic instability could therefore reduce the effective intracellular concentration relative to that present in the isolated kinase assay. These possibilities were not directly measured in the present study and should consequently be regarded as potential explanations rather than established mechanisms.

**Fig. 4:**
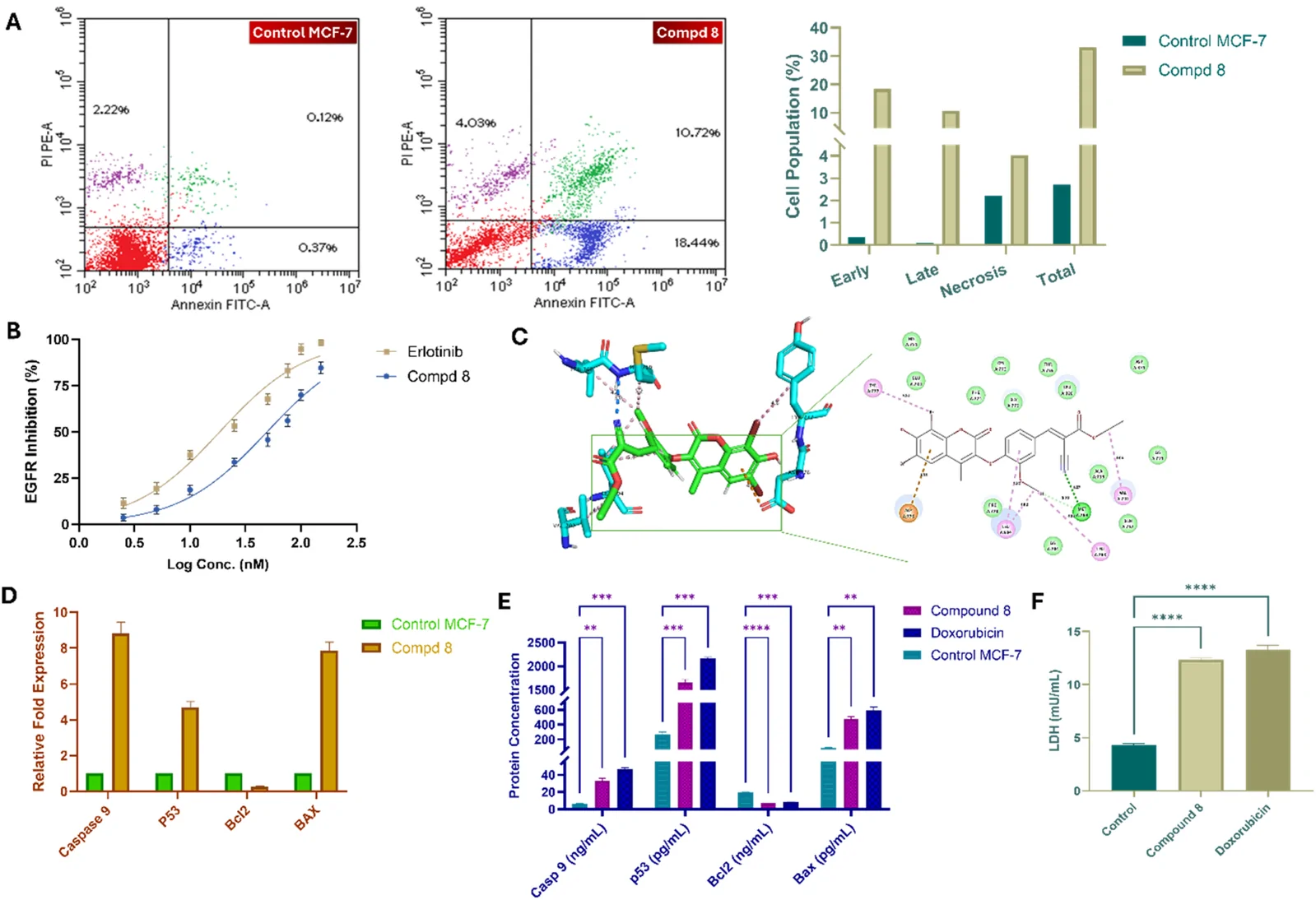
Compound 8 induces apoptosis by inhibiting EGFR signaling and activating the intrinsic apoptotic pathway in MCF-7 cells. (A) Representative Annexin V-FITC/PI flow cytometric analysis of untreated and compound 8-treated MCF-7 cells (5.9 µM, 24 h), together with the quantitative distribution of viable, early apoptotic, late apoptotic, and necrotic cell populations. (B) Dose-dependent inhibition of EGFR kinase activity by compound 8 compared with the reference inhibitor erlotinib. (C) Predicted binding mode of compound 8 within the ATP-binding site of EGFR, showing the three-dimensional ligand orientation and the corresponding two-dimensional protein–ligand interaction map (PDB: 1M17). (D) Relative mRNA expression levels of p53, BAX, Bcl-2, and caspase-9 in MCF-7 cells following treatment with compound 8 at its IC_50_ concentration. (E) Protein levels of p53, BAX, BCL-2, and caspase-9 determined by ELISA in MCF-7 cells following treatment with compound 8 or doxorubicin at their respective cellular IC_50_ concentrations, compared with vehicle-control cells. (F) Extracellular LDH activity in MCF-7 cell-culture supernatants following treatment with compound 8 and doxorubicin at their respective cellular IC_50_ concentrations, compared with vehicle-treated control cells. Data are presented as mean ± SD of three independent experiments (*n* = 3). Statistical analysis was performed using one-way ANOVA; **p ≤ 0.01, ***p ≤ 0.001, and ****p ≤ 0.0001.

Having experimentally established the EGFR kinase inhibitory activity of compound 8, molecular docking studies were subsequently performed within the ATP-binding site of EGFR kinase to investigate the potential structural basis of this observed inhibitory activity.^44^ The docking protocol was first validated by redocking the co-crystallized ligand erlotinib, which successfully reproduced the experimental binding orientation with an RMSD value of 1.56 Å (Fig. S4). Considering the high conformational flexibility of erlotinib, this result confirms the reliability of the adopted docking procedure. Compound 8 was predicted to occupy the ATP-binding pocket and establish several interactions with key active-site residues (Fig. 4C and S5). In particular, the predicted pose formed a conventional hydrogen bond with MET769, a residue involved in ligand recognition within the EGFR catalytic domain, together with hydrophobic interactions involving LEU694, VAL702, LEU768, and TYR777. Compound 8 exhibited a more favorable classical docking score (−7.88 kcal mol^−1^) than redocked erlotinib (−5.88 kcal mol^−1^), accompanied by reduced steric repulsion, lower conformational strain, and a more favorable hydrogen-bond contribution (Tables S5–S7). However, this apparent energetic advantage was not accompanied by a superior CNN score; rather, compound 8 showed a lower CNN score than erlotinib. Moreover, the experimental kinase assay demonstrated that compound 8 (IC_50_ = 50 ± 3 nM) was less potent than erlotinib (IC_50_ = 19 ± 1 nM). Accordingly, the more favorable classical docking score should not be interpreted as evidence that compound 8 binds EGFR more strongly than erlotinib. The discrepancy between the classical docking and CNN-based scoring metrics highlights the limitations of using individual computational scores for quantitative affinity ranking and introduces uncertainty regarding the predicted binding geometry. Thus, while the predicted interactions with MET769 and surrounding hydrophobic residues suggest a plausible orientation of compound 8 within the ATP-binding pocket, the docking analysis should be regarded as a structural hypothesis rather than independent evidence of binding affinity. Further structural and/or dynamic studies would be required to validate the specific binding mode. Importantly, the experimentally measured EGFR kinase inhibition provides the primary evidence that compound 8 interferes with EGFR activity.

Because EGFR signaling exerts a major anti-apoptotic influence through regulation of p53, BCL-2 family proteins, and caspase activation,^45^ we next investigated key components of the intrinsic mitochondrial apoptotic pathway at both the transcriptional and protein levels. qRT-PCR analysis revealed that treatment of MCF-7 cells with compound 8 at its IC_50_ concentration significantly upregulated BAX, p53, and caspase-9 mRNA expression by 7.9-, 4.7-, and 8.8-fold, respectively, while BCL-2 expression was reduced to 0.27-fold relative to the vehicle control (Fig. 4D). These coordinated transcriptional changes, particularly the concomitant increase in BAX and suppression of BCL-2, are consistent with a shift toward mitochondrial apoptotic signaling.^46,47^ To determine whether these transcriptional changes were accompanied by corresponding alterations at the protein level, BAX, BCL-2, caspase-9, and p53 proteins were subsequently quantified by ELISA (Fig. 4E). Consistent with the qRT-PCR findings, compound 8 significantly increased BAX protein levels to 481.2 ± 27.0 pg mL^−1^ compared with 84.3 ± 9.3 pg mL^−1^ in vehicle-control cells, corresponding to a 5.71-fold increase. Caspase-9 was similarly elevated from 6.20 ± 0.34 to 33.33 ± 2.64 ng mL^−1^ (5.37-fold), while p53 increased from 263.3 ± 40.5 to 1654.3 ± 65.5 pg mL^−1^ (6.28-fold). In contrast, the anti-apoptotic BCL-2 protein level decreased from 19.62 ± 0.19 to 6.84 ± 0.17 ng mL^−1^, corresponding to 0.35-fold of the control level. Doxorubicin produced the same directional protein changes, increasing BAX, caspase-9, and p53 to 588.1 ± 50.3 pg mL^−1^, 46.44 ± 1.64 ng mL^−1^, and 2167 ± 22 pg mL^−1^, respectively, while reducing BCL-2 to 8.29 ± 0.15 ng mL^−1^. These protein-level findings closely parallel the transcriptional responses and provide complementary evidence that compound 8 modulates the p53/BAX/BCL-2/caspase-9 axis toward activation of the intrinsic apoptotic pathway. The simultaneous inhibition of EGFR signaling and activation of p53-dependent mitochondrial apoptosis suggest a mechanistic connection between upstream suppression of survival pathways and downstream execution of programmed cell death. Similar relationships between EGFR inhibition, modulation of the BAX/Bcl-2 axis, and activation of caspase-dependent apoptosis have been reported in numerous studies investigating targeted anticancer therapies.^48,49^ Therefore, the ability of compound 8 to simultaneously interfere with proliferative signaling and activate mitochondrial apoptosis may explain its pronounced antiproliferative activity against MCF-7 cells.

To further substantiate that the reduction in MCF-7 cell viability was accompanied by actual cellular injury and loss of plasma-membrane integrity, extracellular lactate dehydrogenase (LDH) activity was determined as an independent cytotoxicity endpoint. MCF-7 cells were treated with compound 8 and doxorubicin at their respective cellular IC_50_ concentrations (5.9 and 14.2 µM, respectively), under the same conditions used for the mechanistic cell-death studies. Treatment with compound 8 significantly increased extracellular LDH activity to 12.35 ± 0.35 mU mL^−1^ compared with 4.28 ± 0.15 mU mL^−1^ in vehicle-treated control cells, corresponding to an approximately 2.88-fold increase. Doxorubicin similarly increased LDH activity to 13.27 ± 0.45 mU mL^−1^, representing an approximately 3.10-fold increase relative to control. LDH is a cytosolic enzyme that is released into the extracellular medium following loss of plasma-membrane integrity; accordingly, increased extracellular LDH provides an independent indication that the reduction in cell viability observed following treatment with compound 8 reflects genuine treatment-associated cytotoxicity rather than merely suppression of cellular metabolic activity. The observed increase in LDH following doxorubicin treatment is consistent with previous studies demonstrating enhanced LDH release and membrane permeability in doxorubicin-treated MCF-7 breast cancer cells.^50,51^ When considered together with the Annexin V/PI analysis, which demonstrated a pronounced increase in apoptotic populations but only a modest necrotic fraction, and the coordinated upregulation of p53, BAX, and caspase-9 together with downregulation of Bcl-2, the present findings indicate that compound 8 induces a predominantly apoptotic response that ultimately becomes associated with loss of membrane integrity.

Collectively, the cellular, biochemical, molecular, and computational findings indicate that compound 8 exerts its antiproliferative effect through a multifaceted mechanism involving disruption of microtubule dynamics and G2/M cell-cycle progression, suppression of EGFR-mediated survival signaling, and activation of the intrinsic mitochondrial apoptotic pathway. The marked Annexin V-positive apoptotic population, together with the concordant mRNA and protein-level increases in p53, BAX, and caspase-9 and suppression of BCL-2, provides convergent evidence for activation of the intrinsic apoptotic pathway. The accompanying increase in extracellular LDH activity further confirms that these molecular and cellular alterations culminate in loss of cell integrity and cytotoxicity. Thus, the antiproliferative effect of compound 8 is associated with coordinated mitotic disruption, pro-apoptotic signaling, and subsequent cellular damage, supporting its selection as the principal lead for subsequent *in vivo* evaluation.

### 2.3 *In vivo* biological evaluation

#### 2.3.1 Acute toxicity assessment and antitumor efficacy of compound 8 in EAC-bearing mice

Prior to evaluating its antitumor efficacy, the safety profile of compound 8 was investigated through an acute toxicity study in mice to establish a suitable therapeutic dose range. Compound 8 was administered intraperitoneally at escalating doses ranging from 1 to 300 mg kg^−1^, and animals were monitored for behavioral abnormalities, signs of systemic toxicity, and mortality over a 48 h observation period. Mice receiving doses up to 200 mg kg^−1^ remained healthy and exhibited normal locomotor activity, feeding behavior, grooming, and excretion patterns, with no detectable signs of toxicity. In contrast, administration of 300 mg kg^−1^ produced pronounced lethargy, reduced locomotor activity, exhaustion, and eventual mortality. These findings indicate preliminary acute tolerability of compound 8 over the tested dose range and supported its subsequent *in vivo* evaluation at substantially lower doses.

The Ehrlich ascites carcinoma (EAC) model is widely employed for evaluating antitumor agents because of its rapid proliferation, high transplantability, and ability to mimic several hallmarks of aggressive tumor growth.^52^ In this model, tumor burden is commonly reflected by both viable tumor cell count and ascites accumulation, which correlate with disease progression and survival outcomes.^53,54^ To investigate the antitumor activity of compound 8, viable EAC cell counts were determined following treatment with 5, 10, and 20 mg kg^−1^ doses. As shown in Fig. 5A, untreated EAC-bearing mice exhibited extensive tumor proliferation, reaching a mean cell count of 295.5 ± 16.36 × 10^6^ cells. Treatment with compound 8 significantly reduced tumor cell burden at all tested doses. Administration of 5 mg kg^−1^ reduced the viable cell count to 96.5 ± 27.0 × 10^6^ cells, corresponding to a 67.3% reduction relative to the untreated EAC group (one-way ANOVA, ****p < 0.0001 compared to EAC control). Comparable suppression was observed at 10 mg kg^−1^ (95.83 ± 8.42 × 10^6^ cells) (one-way ANOVA, ****p < 0.0001 compared to EAC control), while the greatest reduction was achieved at 20 mg kg^−1^, which decreased the viable cell count to 81.66 ± 4.03 × 10^6^ cells, representing a 72.4% reduction compared with the EAC control (one-way ANOVA, ****p < 0.0001 compared to EAC control). These findings are in excellent agreement with the *in vitro* data, where compound 8 inhibited MCF-7 cell proliferation through induction of G2/M arrest and apoptosis. Therefore, the *in vivo* suppression of tumor burden may be attributed, at least in part, to the ability of compound 8 to interfere with cancer cell proliferation and survival pathways.

**Fig. 5:**
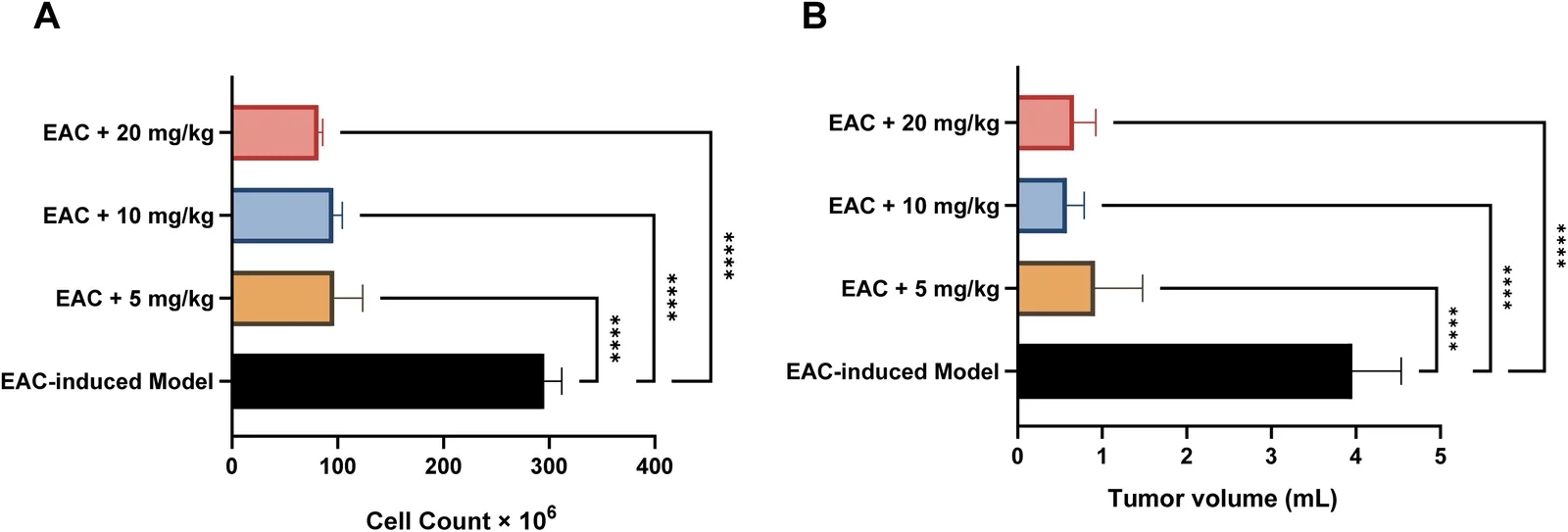
Effect of compound 8 on tumor burden in EAC-bearing mice. (A) Viable EAC cell count following treatment with compound 8 at 5, 10, and 20 mg kg^−1^. (B) Ascitic tumor volume in untreated and compound 8-treated EAC-bearing mice. Data are expressed as mean ± SD (*n* = 6). Statistical analysis was performed using one-way ANOVA; significance was considered at *p* ≤ 0.05 (****p ≤ 0.0001, ***p ≤ 0.001, **p ≤ 0.01, and *p ≤ 0.05).

Ascites volume was subsequently evaluated as an additional indicator of tumor progression. Accumulation of ascitic fluid in EAC-bearing mice reflects enhanced tumor growth, increased vascular permeability, inflammatory responses, and metabolic activity of malignant cells. As shown in Fig. 5B, untreated EAC-bearing mice accumulated a mean ascites volume of 3.95 ± 0.57 mL. Administration of 5 mg kg^−1^ reduced the ascites volume to 0.91 ± 0.56 mL (one-way ANOVA, ****p < 0.0001 compared to EAC control), corresponding to a 76.8% reduction relative to the EAC control. Further suppression was observed at 10 mg kg^−1^, which reduced ascites accumulation to 0.58 ± 0.20 mL (85.3% reduction, one-way ANOVA, ****p < 0.0001 compared to EAC control). Similarly, treatment with 20 mg kg^−1^ resulted in an ascites volume of 0.66 ± 0.25 mL, representing an 83.2% reduction compared with untreated animals (one-way ANOVA, ****p < 0.0001 compared to EAC control). The substantial reduction in ascites accumulation further supports the potent antitumor activity of compound 8*in vivo*. The highest effective dose of compound 8 (20 mg kg^−1^) was approximately 15-fold lower than the first dose associated with overt toxicity (300 mg kg^−1^), suggesting a favorable preliminary safety margin. Although all three doses of compound 8 significantly reduced viable EAC cell count and ascitic tumor volume compared with the untreated EAC group, pairwise comparisons among the 5, 10, and 20 mg kg^−1^ treatment groups revealed no statistically significant differences for either endpoint. Collectively, these findings reveal that compound 8 possesses a favorable safety profile and exerts pronounced antitumor activity in EAC-bearing mice, as evidenced by significant reductions in both viable tumor cell counts and ascites volume. The observed *in vivo* efficacy is consistent with the antiproliferative and pro-apoptotic mechanisms identified *in vitro* and supports the continued investigation of compound 8 as a promising anticancer lead candidate. Despite the absence of significant differences among the three doses in the gross tumor-burden endpoints, all treatment groups were retained for subsequent biochemical and molecular analyses to determine whether the mechanisms associated with compound 8 exhibited dose-related modulation.

#### 2.3.2 Compound 8 attenuates EAC-induced hepatic injury and restores liver function

Since liver dysfunction frequently accompanies tumor progression and systemic cancer-associated inflammation,^55^ the hepatoprotective potential of compound 8 was evaluated through determination of serum aminotransferase activities and histopathological examination of liver tissues. Serum alanine aminotransferase (ALT) and aspartate aminotransferase (AST) are among the most widely used biomarkers of hepatocellular injury, as their elevation reflects membrane damage and leakage of intracellular enzymes into the circulation.^56^ As shown in Fig. 6, EAC induction resulted in marked hepatic dysfunction, evidenced by significant elevations in both AST and ALT levels compared with normal animals. Serum AST increased from 48.5 ± 2.8 U L^−1^ in the untreated control group to 158 ± 7.59 U L^−1^ in EAC-bearing mice, corresponding to a 226% increase above the physiological baseline. Similarly, ALT levels increased from 30.66 ± 3.01 U L^−1^ to 132.33 ± 5.27 U L^−1^, representing a 331% elevation. These findings support substantial hepatocellular damage associated with EAC progression and are consistent with previous reports demonstrating that EAC growth induces oxidative stress, inflammatory responses, and hepatic tissue injury. Treatment with compound 8 significantly ameliorated these biochemical alterations in a dose-dependent manner. AST levels were reduced to 109.3 ± 3.7, 84.16 ± 5.34, and 60.17 ± 3.55 U L^−1^ following administration of 5, 10, and 20 mg kg^−1^, respectively (one-way ANOVA, ****p < 0.0001 compared to EAC control). Likewise, ALT levels decreased to 87.16 ± 7.88, 55.66 ± 4.17, and 39.0 ± 3.16 U L^−1^ at the corresponding doses (one-way ANOVA, ****p < 0.0001 compared to EAC control) (Fig. 6). Notably, the highest dose restored both enzymes to values approaching the physiological range, indicating substantial protection against EAC-induced hepatic injury. The observed normalization of serum transaminases suggests preservation of hepatocyte membrane integrity and attenuation of liver damage in treated animals.

**Fig. 6:**
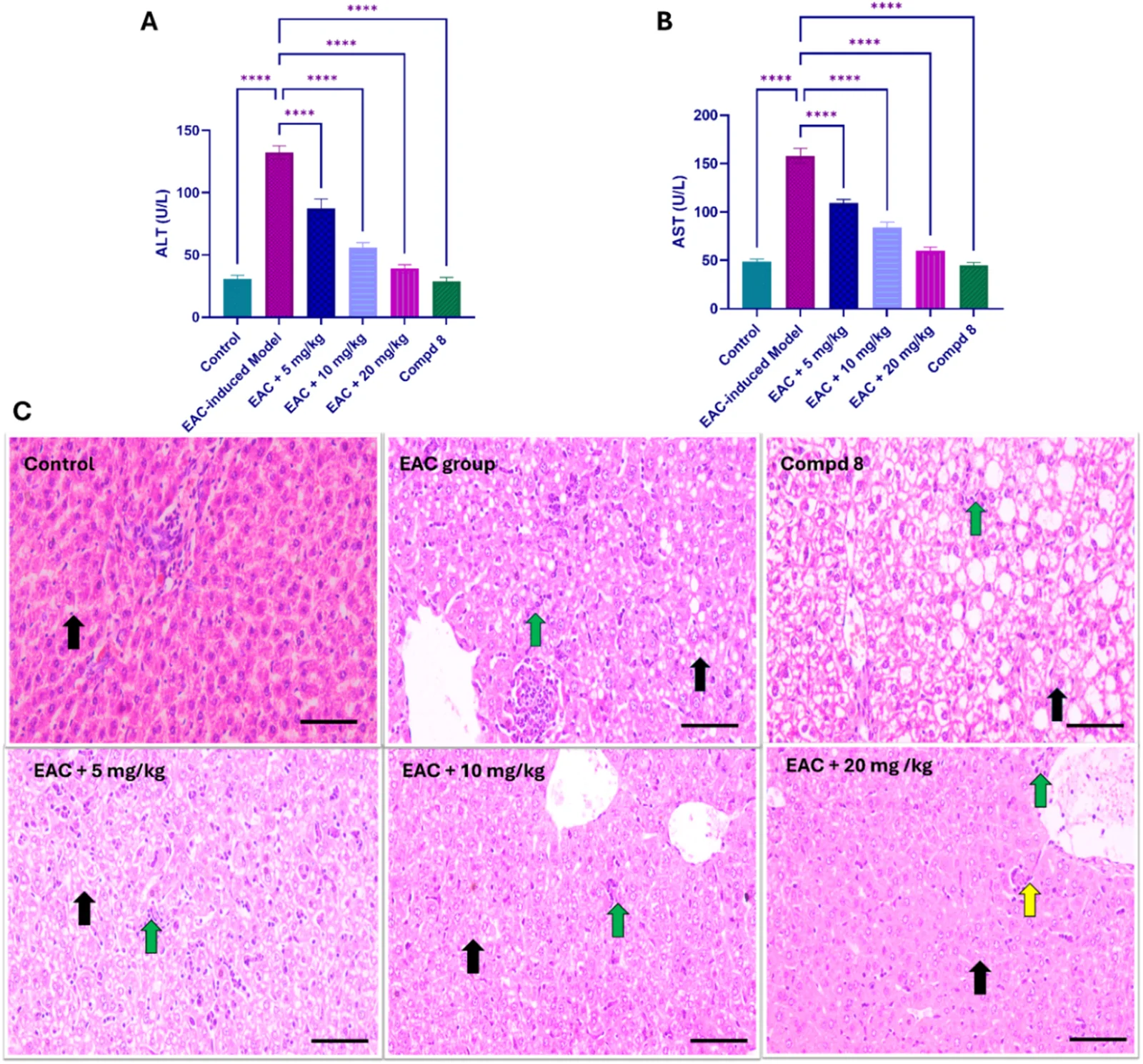
Effect of compound 8 on liver function and hepatic histopathology in EAC-bearing mice. (A) Expression of serum alanine aminotransferase (ALT). (B) Expression of serum aspartate aminotransferase (AST). Data are presented as mean ± SD (*n* = 6). Statistical analysis was performed using one-way ANOVA, and significance was considered significant at *p* ≤ 0.05 (****p ≤ 0.0001, ***p ≤ 0.001, **p ≤ 0.01, and *p ≤ 0.05). (C) Representative hematoxylin and eosin (H&E)-stained liver sections from the experimental groups (40× magnification). The untreated control group exhibited normal hepatic architecture with preserved hepatocyte cords and intact sinusoids. The EAC group showed mild steatosis, marked hepatocellular ballooning, and lobular inflammatory infiltration. Treatment with compound 8 ameliorated these histopathological alterations in a dose-dependent manner, resulting in reduced hepatocellular injury and inflammatory changes.

Histopathological findings further supported the biochemical data. Liver sections obtained from untreated control mice displayed normal hepatic architecture, characterized by preserved hepatocellular cords, intact sinusoids, and the absence of steatosis, inflammation, ballooning degeneration, or fibrosis (steatosis = 0, ballooning = 0, inflammation = 0, fibrosis = 0). In contrast, EAC-bearing mice exhibited pronounced pathological alterations, including mild steatosis (score 1), marked hepatocellular ballooning (score 2), and moderate lobular inflammation (score 2), indicating substantial hepatic injury induced by tumor burden, while fibrosis remained absent (score 0) (Fig. 6). Administration of compound 8 markedly improved liver histology. The 5 mg kg^−1^-treated group displayed complete resolution of steatosis (score 0), accompanied by reduced hepatocellular ballooning (score 1) and mild inflammatory infiltration (score 1), indicating partial restoration of hepatic architecture. Similarly, the 10 mg kg^−1^ group exhibited no evidence of steatosis (score 0), persistent ballooning changes (score 2), and only mild inflammation (score 1), reflecting attenuation of EAC-induced hepatic injury. The 20 mg kg^−1^ group showed complete absence of steatosis (score 0) and only mild inflammation (score 1), while hepatocellular ballooning remained evident (score 2). In addition, mild sinusoidal fibrosis (score 1a) was observed despite the marked improvement in serum liver enzymes and reduction of inflammatory lesions (Fig. 6). Although the fibrotic changes were limited in severity, this observation warrants further investigation to determine whether prolonged administration at higher doses may influence extracellular matrix remodeling. Nevertheless, the overall histopathological profile remained markedly improved compared with untreated EAC-bearing mice. Notably, liver sections from the compound 8-only group revealed the absence of steatosis or fibrosis (scores 0 and 0, respectively). These findings suggest that compound 8 did not induce overt structural liver damage or fibrotic remodeling under normal physiological conditions (Fig. 6). The biochemical and histological findings are highly consistent and collectively indicate that compound 8 effectively protects against EAC-associated hepatic damage. Restoration of liver function may contribute to the overall antitumor efficacy observed *in vivo*, as preservation of hepatic integrity is essential for maintaining systemic metabolic homeostasis during cancer progression. Together with the observed reductions in tumor burden and ascites accumulation, these findings further support the therapeutic potential of compound 8 as a promising anticancer candidate.

#### 2.3.3 Compound 8 ameliorates EAC-induced renal dysfunction without causing structural kidney damage

Since renal impairment frequently accompanies tumor progression and systemic metabolic disturbances associated with advanced cancer,^57^ the effect of compound 8 on kidney function was evaluated by measuring serum creatinine and urea levels and correlating these findings with renal histopathology. As shown in Fig. 7, EAC induction resulted in marked renal dysfunction, evidenced by significant elevations in serum creatinine and urea levels compared with untreated animals. Serum creatinine increased from 0.52 ± 0.08 mg dL^−1^ in the untreated control group to 1.88 ± 0.12 mg dL^−1^ in EAC-bearing mice, corresponding to a 3.6-fold increase. Likewise, serum urea increased from 24.50 ± 1.87 mg dL^−1^ to 73.83 ± 6.05 mg dL^−1^, representing a 3.0-fold elevation. These findings indicate substantial impairment of renal function following EAC progression and are consistent with previous reports demonstrating that tumor burden can induce systemic inflammation, oxidative stress, and subsequent renal dysfunction.^58^ Treatment with compound 8 significantly restored kidney function in a dose-dependent manner. Serum creatinine levels decreased to 1.15 ± 0.05, 0.93 ± 0.05, and 0.65 ± 0.05 mg dL^−1^ following administration of 5, 10, and 20 mg kg^−1^ (one-way ANOVA, ****p < 0.0001 compared to EAC control), respectively, corresponding to reductions of 38.8%, 50.5%, and 65.4% relative to the EAC control group. Similarly, serum urea levels were reduced to 54.33 ± 4.23, 40.83 ± 2.32, and 30.83 ± 2.32 mg dL^−1^ at the respective doses (one-way ANOVA, ****p < 0.0001 compared to EAC control), representing reductions of 26.4%, 44.7%, and 58.2% compared with untreated EAC-bearing animals (Fig. 7). Notably, the highest dose restored both biomarkers to values approaching those of the untreated control group, while the compound 8-only group exhibited near-complete normalization of creatinine (0.55 ± 0.05 mg dL^−1^) and urea (26.17 ± 2.64 mg dL^−1^). Importantly, the close approximation of creatinine and urea values to normal physiological levels at 20 mg kg^−1^ indicates that compound 8 effectively counteracts the renal dysfunction associated with tumor progression. These findings complement the previously observed reductions in tumor burden and ascites volume, suggesting that attenuation of systemic tumor-associated toxicity may contribute to preservation of renal function.

**Fig. 7:**
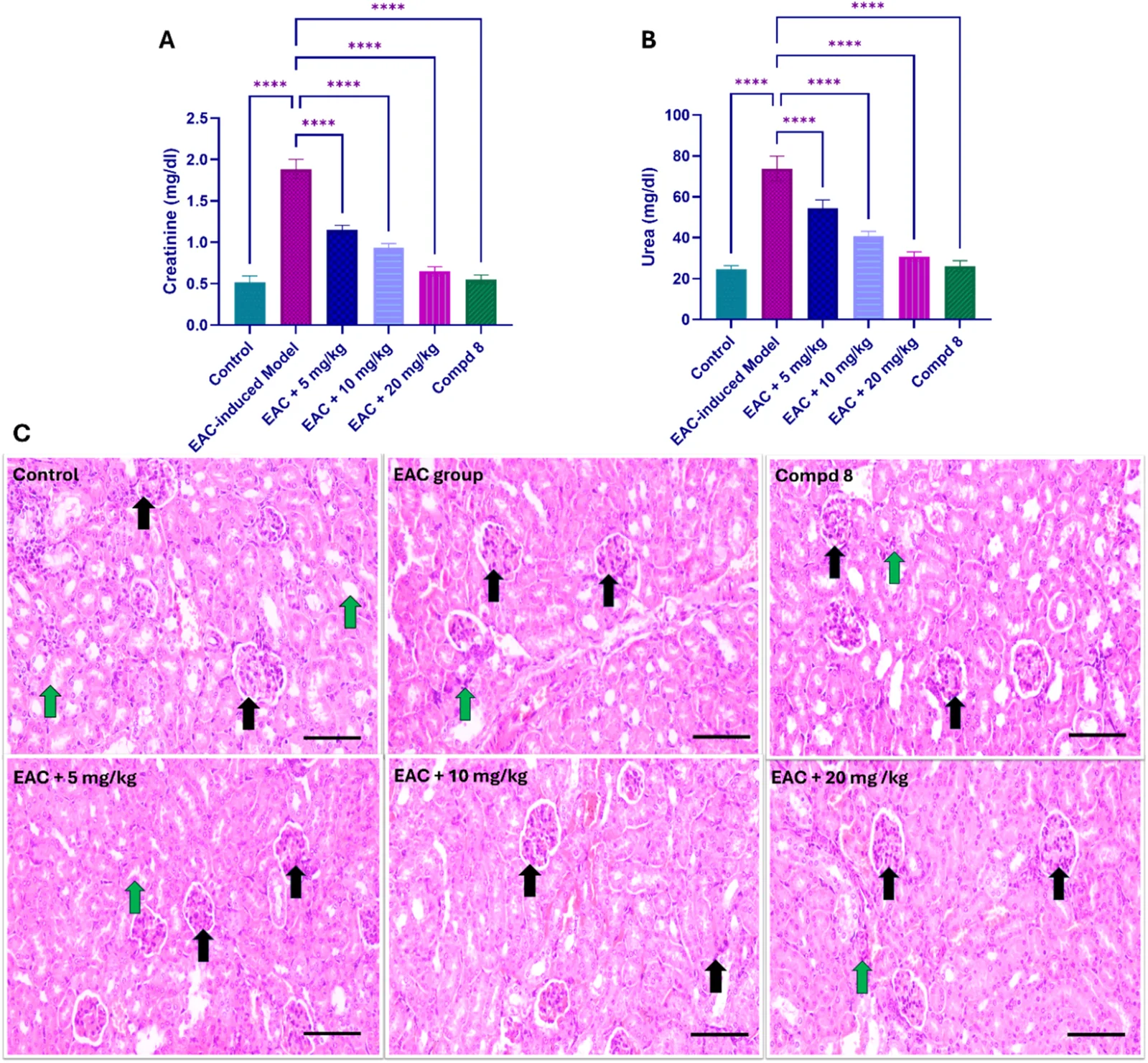
Effect of compound 8 on renal function and kidney histopathology in EAC-bearing mice. (A) Serum creatinine levels in the different experimental groups. (B) Serum urea levels in the different experimental groups. Data are presented as mean ± SD (*n* = 6). Statistical significance was determined using one-way ANOVA, with significance defined as *p* ≤ 0.05 (****p ≤ 0.0001, ***p ≤ 0.001, **p ≤ 0.01, and *p ≤ 0.05). (C) Representative H&E-stained kidney sections from the experimental groups (40× magnification). The untreated control group exhibited normal renal architecture with preserved glomeruli and renal tubules. Mild interstitial inflammatory cell infiltration was observed in EAC-bearing mice without apparent glomerular or tubular degeneration. Treatment with compound 8 preserved normal renal histological architecture across all dose levels.

Histopathological examination of H&E-stained kidney sections further supported the biochemical findings. Renal tissues from untreated control mice displayed normal cortical architecture, characterized by intact glomeruli with preserved basement membranes and well-organized renal tubules lined by normal cuboidal epithelium (Fig. 7). Although EAC-bearing mice exhibited mild interstitial inflammatory cell infiltration, no significant glomerular or tubular degeneration was observed. Importantly, treatment with compound 8 preserved normal renal architecture across all dose levels. Kidney sections from the 5 mg kg^−1^-treated group were virtually indistinguishable from the untreated control group, whereas the 10 mg kg^−1^ and 20 mg kg^−1^ groups exhibited no evidence of glomerulosclerosis, tubular atrophy, or fibrotic remodeling. The absence of significant degenerative or fibrotic changes in compound 8-treated control animals indicates that the compound does not exert detectable nephrotoxic effects under the experimental conditions employed. Collectively, the biochemical and histopathological findings indicate that compound 8 effectively ameliorates EAC-induced renal dysfunction while preserving normal kidney architecture. The restoration of renal biomarkers, together with the absence of significant structural damage, further supports the favorable safety profile of compound 8 and reinforces its therapeutic potential as an anticancer agent.

#### 2.3.4 Compound 8 attenuates oxidative stress and inflammatory responses in EAC-bearing mice

Oxidative stress is a hallmark of tumor progression and contributes significantly to cancer-associated tissue injury through excessive generation of reactive oxygen species (ROS).^59,60^ Therefore, the effect of compound 8 on hepatic oxidative stress status was investigated by determining lipid peroxidation levels together with enzymatic and non-enzymatic antioxidant defenses. As shown in Fig. 8, EAC induction resulted in profound oxidative stress, evidenced by a marked increase in malondialdehyde (MDA) levels accompanied by substantial depletion of endogenous antioxidant systems. Hepatic MDA levels increased from 8.13 ± 0.93 nmol g^−1^ tissue in untreated control animals to 40.67 ± 2.99 nmol g^−1^ tissue in EAC-bearing mice, representing an approximately five-fold increase. Since MDA is a major end-product of lipid peroxidation, this finding indicates extensive ROS-mediated membrane damage associated with tumor progression. Treatment with compound 8 significantly reduced MDA levels in a dose-dependent manner. MDA decreased to 26.90 ± 1.89, 18.45 ± 1.24, and 11.85 ± 0.81 nmol g^−1^ tissue following administration of 5, 10, and 20 mg kg^−1^, respectively (one-way ANOVA, ****p < 0.0001 compared to EAC control). At the highest dose, MDA levels approached those observed in untreated animals, indicating substantial suppression of lipid peroxidation.

**Fig. 8:**
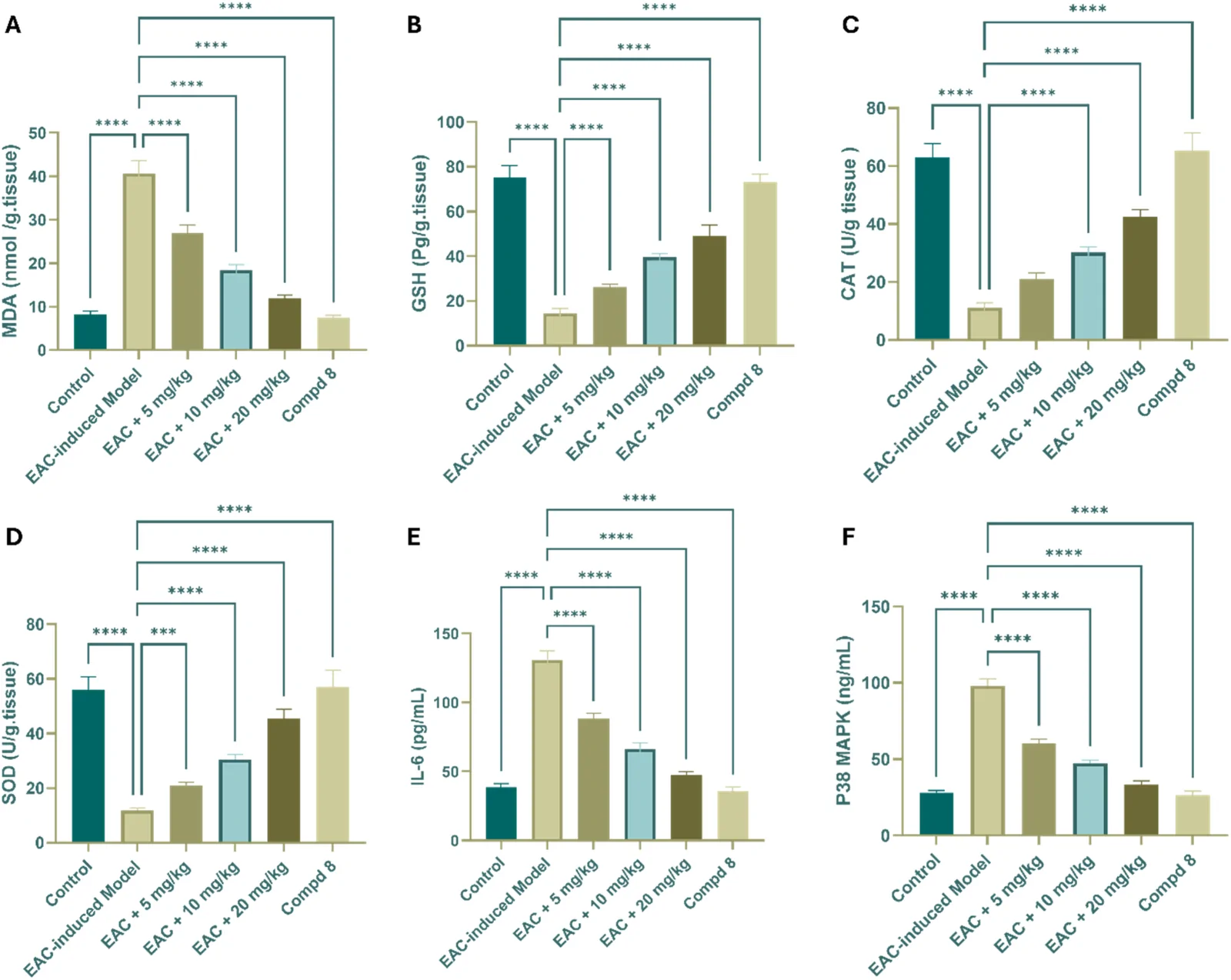
Effect of compound 8 on hepatic oxidative stress and inflammatory markers in EAC-bearing mice. (A)–(D) Hepatic oxidative stress parameters, including malondialdehyde (MDA, A), reduced glutathione (GSH, B), catalase (CAT, C), and superoxide dismutase (SOD, D). (E)–(F) Inflammatory markers, including interleukin-6 (IL-6, E) and p38 MAPK (F). Data are presented as mean ± SD (*n* = 6). Statistical significance was determined using one-way ANOVA, with significance defined as *p* ≤ 0.05 (****p ≤ 0.0001, ***p ≤ 0.001, **p ≤ 0.01, and *p ≤ 0.05).

The increase in lipid peroxidation observed in EAC-bearing mice was accompanied by marked depletion of the endogenous antioxidant defense system. Reduced glutathione (GSH), the principal intracellular non-enzymatic antioxidant, declined from 75.10 ± 5.33 pg g^−1^ tissue in untreated animals to 14.57 ± 2.08 pg g^−1^ tissue following EAC induction, corresponding to an approximately 81% reduction. Likewise, catalase (CAT) activity decreased from 63.08 ± 4.62 to 11.17 ± 1.64 U g^−1^ tissue, while superoxide dismutase (SOD) activity declined from 55.95 ± 4.87 to 11.76 ± 0.96 U g^−1^ tissue, representing reductions of approximately 82% and 79%, respectively (Fig. 8). These findings indicate severe impairment of both enzymatic and non-enzymatic antioxidant defenses in response to tumor-associated oxidative stress. Administration of compound 8 significantly restored antioxidant capacity in a dose-dependent manner. GSH levels increased to 26.20 ± 1.43, 39.58 ± 1.60, and 49.13 ± 4.71 pg g^−1^ tissue following treatment with 5, 10, and 20 mg kg^−1^, respectively (one-way ANOVA, ****p < 0.0001 compared to EAC control). Similarly, CAT activity improved to 20.97 ± 2.12, 30.27 ± 1.92, and 42.63 ± 2.34 U g^−1^ tissue (one-way ANOVA, ****p < 0.0001 compared to EAC control), while SOD activity increased to 21.12 ± 0.98, 30.48 ± 1.82, and 45.45 ± 3.53 U g^−1^ tissue at the corresponding doses (one-way ANOVA, ****p < 0.0001 compared to EAC control). Notably, the highest dose restored SOD activity to approximately 81% of the untreated control value, representing the most pronounced recovery among the antioxidant parameters evaluated. The observed restoration of antioxidant defenses, together with the marked reduction in MDA levels, suggests that compound 8 effectively mitigates EAC-induced oxidative stress. These findings reveal that the ability of compound 8 to restore redox homeostasis may contribute significantly to its overall antitumor and tissue-protective activities. Similar relationships between restoration of antioxidant defenses, suppression of oxidative damage, and attenuation of cancer-associated inflammation have been extensively reported in experimental tumor models.^61,62^

Because oxidative stress is a potent activator of p38 MAPK signaling,^63,64^ we next investigated whether the restoration of redox homeostasis observed following treatment with compound 8 was accompanied by suppression of this stress-responsive inflammatory pathway. As shown in Fig. 8, EAC induction resulted in marked activation of p38 MAPK from 28.07 ± 1.44 ng mL^−1^ in untreated animals to 98.22 ± 4.31 ng mL^−1^ in EAC-bearing mice, representing an approximately 3.5-fold elevation. This finding is consistent with the severe oxidative stress observed in the EAC group and supports previous reports demonstrating activation of p38 MAPK in response to oxidative injury and inflammatory stimuli within the liver.^63,64^ Treatment with compound 8 significantly suppressed p38 MAPK activation in a dose-dependent manner. p38 MAPK levels declined to 60.15 ± 2.92, 47.38 ± 2.03, and 33.37 ± 2.44 ng mL^−1^ following administration of 5, 10, and 20 mg kg^−1^, respectively (one-way ANOVA, ****p < 0.0001 compared to EAC control). The highest dose reduced p38 MAPK by approximately 66% relative to the EAC control group and restored the marker to values approaching the physiological baseline. Similarly, the compound 8-treated control group exhibited near-complete normalization of p38 MAPK levels (26.65 ± 2.43 ng mL^−1^). The progressive reduction in p38 MAPK closely paralleled the restoration of antioxidant defenses and the decline in lipid peroxidation observed, suggesting that attenuation of oxidative stress contributes substantially to suppression of p38 MAPK signaling.

Immunohistochemical analysis further corroborated the biochemical findings (Fig. 9). Liver sections from untreated control animals exhibited no detectable p38 MAPK immunoreactivity (total score = 0), indicating minimal basal activation of this pathway under physiological conditions. In contrast, the EAC group demonstrated strong cytoplasmic p38 MAPK expression in 56.3 ± 6.7% of hepatocytes (percentage score = 4, intensity score = 3, total score = 7), confirming pronounced activation of this stress-responsive kinase in tumor-bearing animals. Administration of compound 8 markedly reduced hepatic p38 MAPK immunostaining. The 5 mg kg^−1^-treated group exhibited only weak staining in 2.3 ± 2.5% of hepatocytes (percentage score = 1, intensity score = 1, total score = 2), whereas the 10 mg kg^−1^ group displayed moderate residual expression in 15.3 ± 4.6% of hepatocytes (percentage score = 2, intensity score = 2, total score = 4). Notably, complete absence of detectable p38 MAPK immunoreactivity was observed in the 20 mg kg^−1^-treated group (total score = 0), which closely resembled both the untreated control and reference drug groups. The marked suppression of p38 MAPK observed at both the biochemical and tissue levels provides strong evidence that compound 8 effectively attenuates activation of this stress-responsive signaling pathway. Since p38 MAPK is activated by oxidative stress and contributes to the production of pro-inflammatory mediators, including IL-6, the observed reduction in p38 MAPK signaling may represent a key mechanistic link between restoration of antioxidant defenses and suppression of hepatic inflammation.

**Fig. 9:**
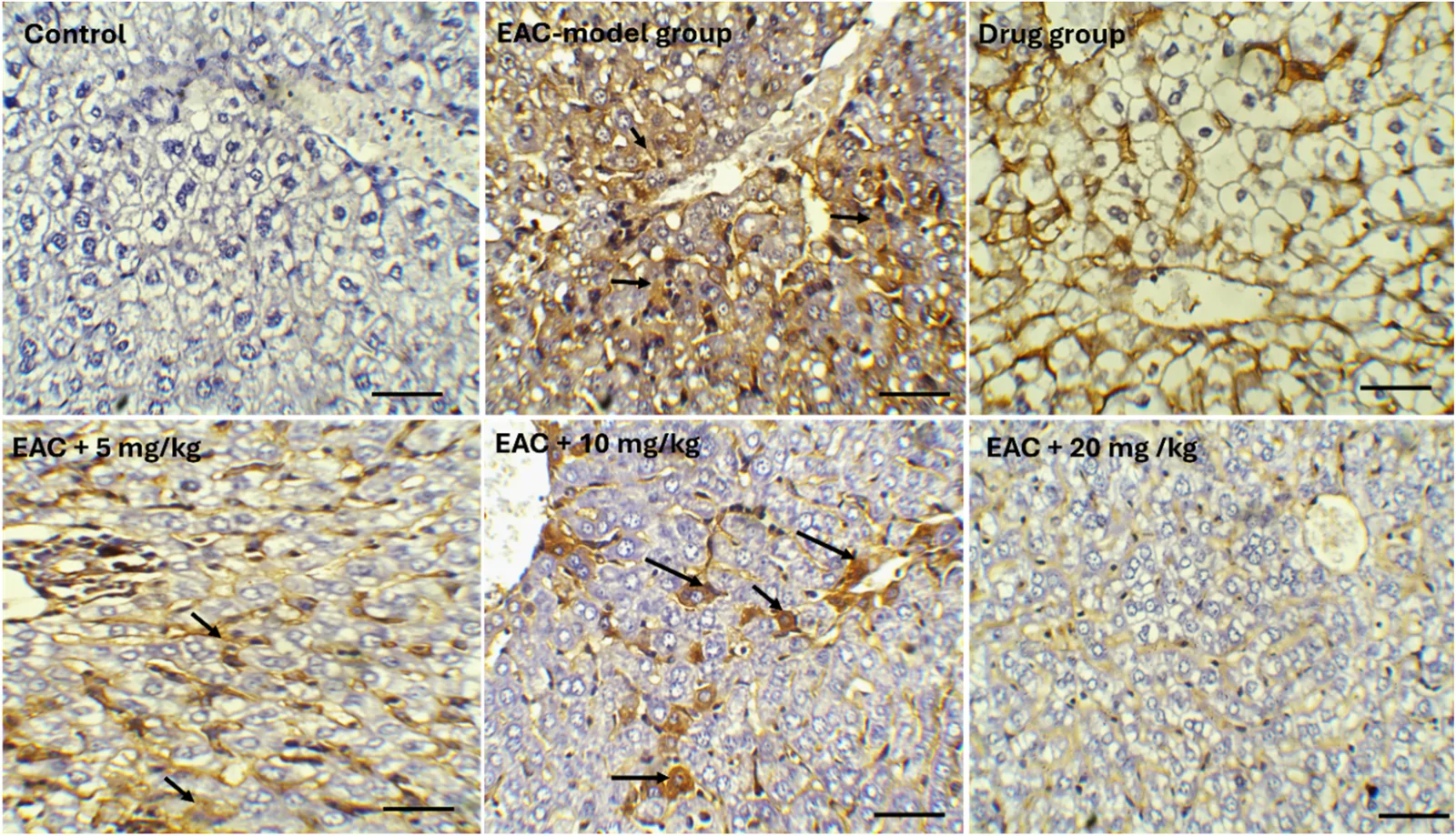
Representative immunohistochemical (IHC) staining of hepatic p38 MAPK expression in the experimental groups (40× magnification; scale bar = 100 µm). No p38 MAPK immunoreactivity was detected in the untreated control group. Strong cytoplasmic p38 MAPK staining was observed in hepatocytes from EAC-bearing mice (black arrows). Treatment with compound 8 reduced p38 MAPK expression in a dose-dependent manner, with weak staining observed at 5 mg kg^−1^, moderate residual staining at 10 mg kg^−1^, and complete absence of detectable immunoreactivity at 20 mg kg^−1^. The compound 8-treated control group exhibited a staining pattern comparable to the untreated control, with only occasional non-specific sinusoidal staining.

IL-6 is a pleiotropic cytokine that contributes to tumor progression, inflammatory responses, and tissue injury and is known to be regulated, at least in part, through stress-responsive signaling pathways including p38 MAPK.^65^ Therefore, suppression of IL-6 would provide further evidence for the anti-inflammatory activity of compound 8. Given the marked suppression of p38 MAPK signaling observed following treatment with compound 8, we next investigated whether this effect was accompanied by attenuation of IL-6 production. As shown in Fig. 8, EAC induction resulted in a marked elevation of serum IL-6 levels. The untreated control group exhibited a basal IL-6 concentration of 38.95 ± 2.40 pg mL^−1^, whereas EAC-bearing mice showed a significant increase to 130.95 ± 6.76 pg mL^−1^, corresponding to an approximately 3.4-fold elevation. This pronounced increase is consistent with the inflammatory state associated with tumor progression and further supports the involvement of inflammatory signaling pathways in EAC-induced hepatic injury. Treatment with compound 8 significantly attenuated the EAC-induced increase in IL-6 in a dose-dependent manner. Serum IL-6 levels declined to 88.28 ± 3.87, 66.33 ± 4.36, and 47.52 ± 2.53 pg mL^−1^ following administration of 5, 10, and 20 mg kg^−1^, respectively (one-way ANOVA, ****p < 0.0001 compared to EAC control). Notably, the highest dose reduced IL-6 by approximately 64% relative to the EAC control group and restored cytokine levels to values approaching the physiological baseline. Similarly, the compound 8-treated control group exhibited near-complete normalization of IL-6 concentrations (35.95 ± 3.08 pg mL^−1^). The progressive decline in IL-6 closely paralleled the reductions observed in p38 MAPK activation, suggesting that suppression of inflammatory cytokine production may be linked to inhibition of upstream stress-responsive signaling pathways.

To further validate the biochemical findings, hepatic IL-6 expression was assessed immunohistochemically using the modified Allred scoring system (Fig. 10). Liver sections from untreated control animals exhibited complete absence of IL-6 immunoreactivity (total score = 0), indicating minimal basal cytokine expression under physiological conditions. In contrast, the EAC group displayed strong cytoplasmic IL-6 staining in hepatocytes, with a mean positive area of 72.3 ± 11.2% (percentage score = 5) and strong staining intensity (intensity score = 3), resulting in the highest total score (8). These findings confirm substantial local hepatic activation of inflammatory signaling in tumor-bearing animals. Administration of compound 8 markedly reduced hepatic IL-6 expression. The 5 mg kg^−1^-treated group exhibited moderate residual immunoreactivity, with a mean positive area of 37.7 ± 9.0% (percentage score = 3) and moderate staining intensity (intensity score = 2), yielding a total score of 5. Remarkably, complete absence of detectable IL-6 staining was observed in both the 10 mg kg^−1^ and 20 mg kg^−1^ groups (total score = 0), closely resembling the untreated control and reference drug groups. The complete loss of IL-6 immunoreactivity at these doses provides strong histological evidence for suppression of local inflammatory responses within hepatic tissue. The biochemical and immunohistochemical findings are highly consistent and collectively support that compound 8 effectively attenuates EAC-associated inflammation. Activation of p38 MAPK is known to promote the production of pro-inflammatory cytokines, including IL-6; therefore, the simultaneous suppression of both markers suggests that compound 8 modulates redox-sensitive inflammatory pathways. This interpretation is further supported by the substantial improvement in liver function parameters and histopathological architecture observed in treated animals, indicating that attenuation of oxidative stress and inflammation contributes significantly to the hepatoprotective effects of compound 8.

**Fig. 10:**
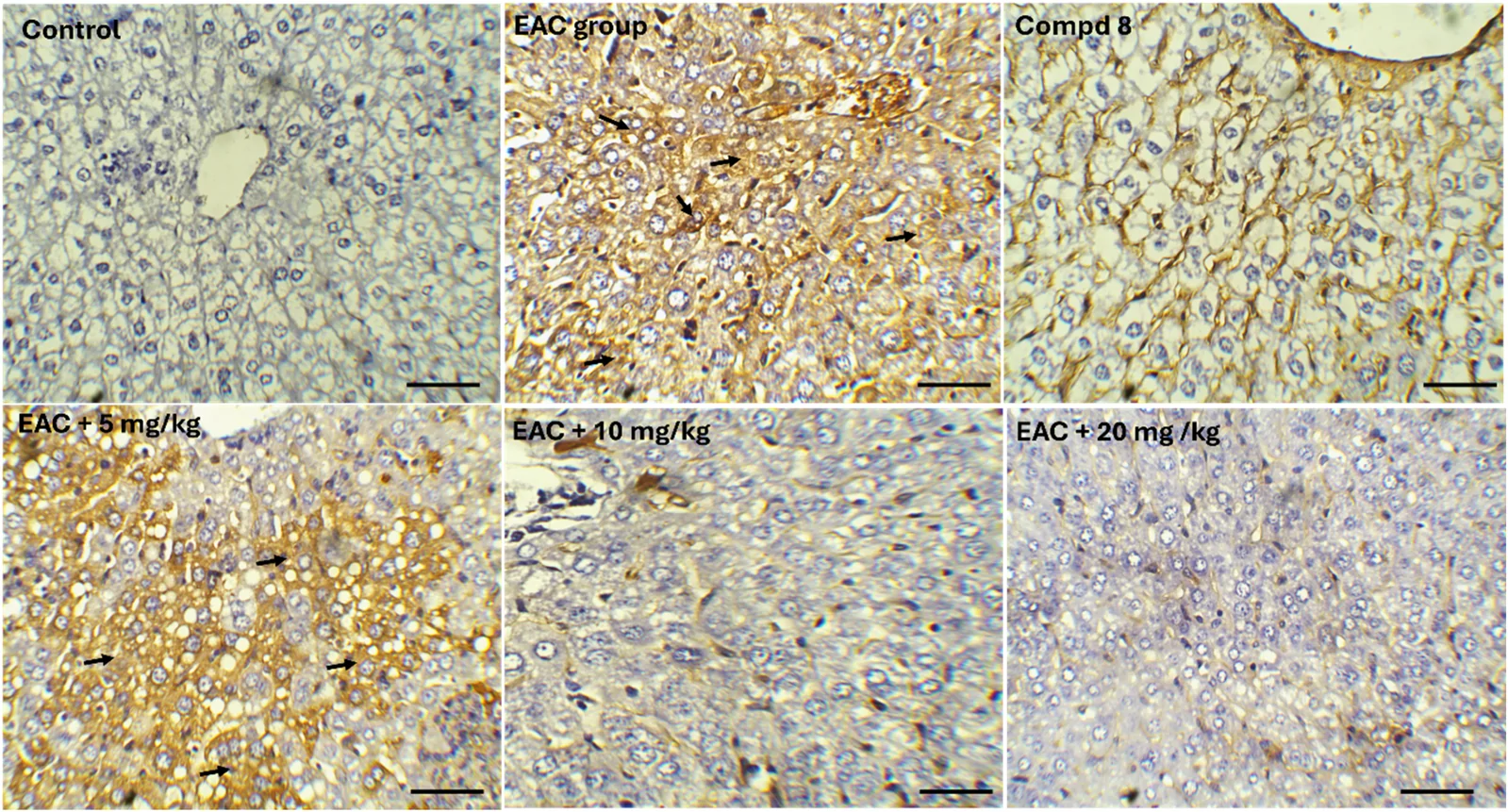
Representative IHC staining of IL-6 expression in liver tissues from the different experimental groups (40× magnification; scale bar = 100 µm). The untreated control group exhibited no detectable IL-6 immunoreactivity in hepatocytes. In contrast, the EAC group showed strong cytoplasmic IL-6 expression in hepatocytes (black arrows). The compound 8-treated control group displayed no detectable IL-6 immunoreactivity, comparable to the untreated control group. Treatment with compound 8 resulted in a dose-dependent reduction in hepatic IL-6 expression. The 5 mg kg^−1^-treated group exhibited moderate residual cytoplasmic staining in hepatocytes (black arrows), whereas complete absence of detectable IL-6 immunoreactivity was observed in both the 10 mg kg^−1^ and 20 mg kg^−1^-treated groups.

Collectively, the present findings suggest that compound 8 exerts its anticancer activity through a multifaceted mechanism involving both direct cytotoxic effects on tumor cells and modulation of the tumor-associated oxidative and inflammatory microenvironment (Fig. 11). *In vitro*, compound 8 inhibited tubulin polymerization, resulting in a pronounced G2/M phase cell cycle arrest and subsequent induction of apoptosis. This pro-apoptotic effect was associated with suppression of EGFR signaling and activation of the intrinsic mitochondrial apoptotic pathway, as evidenced by concordant transcriptional and protein-level increases in p53, BAX, and caspase-9 together with suppression of BCL-2, ultimately promoting programmed cell death in MCF-7 cells. *In vivo*, compound 8 significantly reduced EAC tumor burden and ascites formation while simultaneously restoring redox homeostasis through suppression of lipid peroxidation (MDA) and enhancement of endogenous antioxidant defenses (GSH, CAT, and SOD). Restoration of the antioxidant status was accompanied by marked inhibition of the stress-responsive p38 MAPK pathway and subsequent suppression of IL-6-mediated inflammatory signaling, findings that were further confirmed by immunohistochemical analyses. These molecular alterations were associated with substantial improvements in liver and kidney function, as demonstrated by normalization of serum ALT, AST, creatinine, and urea levels together with marked amelioration of tissue histopathological abnormalities. Therefore, the observed biological activity of compound 8 is likely mediated through the convergence of antiproliferative, pro-apoptotic, antioxidant, and anti-inflammatory mechanisms, supporting its further investigation as a promising anticancer lead compound. Despite the significant antitumor effects observed in EAC-bearing mice, the translational scope of the present *in vivo* model should be considered. Although EAC is a widely used transplantable murine tumor model of mammary origin, the ascitic form employed herein does not reproduce the anatomical localization, solid-tumor architecture, stromal interactions, or tumor microenvironment of an orthotopic breast tumor. Accordingly, the present *in vivo* findings support the antitumor activity of compound 8 in the EAC ascites model but should not be interpreted as direct evidence of efficacy against orthotopic breast cancer. Further evaluation in orthotopic and other clinically relevant solid breast-tumor models will therefore be required to establish the breast-cancer-specific translational potential of compound 8.

**Fig. 11:**
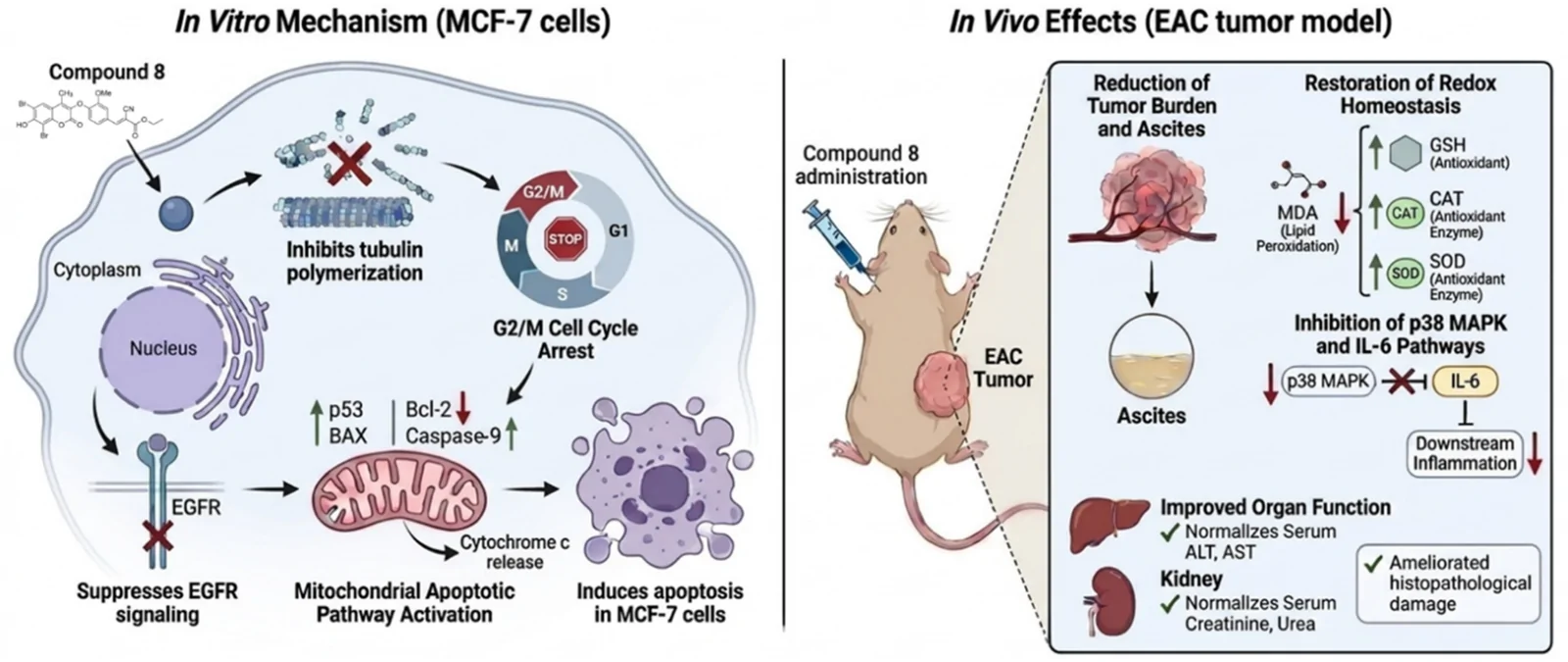
Summary of the proposed mechanism of *in vitro* and *in vivo* antiproliferative activity of compound 8.

## 3. Materials and methods

### 3.1 Chemistry

#### 3.1.1 General information

Unless otherwise stated, all chemicals and solvents were purchased from commercial suppliers (Sigma-Aldrich, Acros Organics, and TCI Chemicals) and used without further purification. Nuclear magnetic resonance (NMR) spectra were recorded on a Bruker Avance DRX 400 spectrometer operating at 400 MHz for ^1^H-NMR and 100 MHz for ^13^C-NMR using DMSO-d_6_ or CDCl_3_ as deuterated solvents. Chemical shifts (*δ*) are reported in parts per million (ppm) relative to the corresponding residual solvent signals. Melting points were determined using a Reichert hot-stage melting point apparatus and are reported without correction. Infrared (IR) spectra were recorded on a Shimadzu FTIR spectrophotometer using KBr discs. Elemental microanalyses (C, H, and N) were performed using a PerkinElmer 2400 Series II elemental analyzer, and the obtained values were within ±0.4% of the calculated values. The synthetic intermediates 7-hydroxy-4-methylcoumarin (2), 6,8-dibromo-7-hydroxy-4-methylcoumarin (3), substituted benzaldehydes 5a–b, and cyanoacrylate derivatives 6a–d were prepared according to previously reported procedures.^35,36,66,67^

#### 3.1.2 Synthetic procedures and compound characterization

##### General procedure for the synthesis of novel chromen-2-yl acrylate derivatives (7, 8, 11, 12)

A solution of α,β-unsaturated cyanoacrylates 6a–d (1.8 mmol, 1 equiv.) in dry ethanol (18 mL) was treated with Cs_2_CO_3_ (5.4 mole, 3 equiv.) and the resulting mixture was refluxed for 2-4 h, before it was treated with a solution of compound 3 (2.25 mmol, 1.25 equiv.). The obtained reaction mixture was allowed to stir under reflux for additional 16–24 h, as judged by TLC analysis for a complete reaction. The reaction was cooled to 25 °C, and subsequently quenched with water. The mixture was extracted with diethyl-ether (3 × 100 mL), and the combined organic layers were washed with brine, dried over magnesium sulphate, and filtered. Concentration of collected layers under reduced pressure provided a crude solid, which was finally purified by recrystallization using ethanol to afford the desired products 7, 8, 11, and 12.

###### Ethyl-2-cyano-3-(4-((6,8-dibromo-7-hydroxy-4-methyl-2-oxo-2*H*-chromen-3-yl)-oxy)-phenyl)acrylate (7)

Obtained as pale-yellow powder, yield 64%, m.p. 202–204 °C. IR_(KBr)_*λ*_max_ (cm^−1^) = 3317 (OH), 2226 (CN), 1711 (C=O), 1584, 1516 (C=C), 1015 (C–O). ^1^H-NMR (DMSO-d_6_, 400 MHz, ppm) *δ*: 1.28–1.32 (t, 3H, CH_3_), 2.92 (s, 3H, CH_3_), 4.26–4.29 (q, 2H, CH_2_), 6.80–7.90 (m, 5H, aromatic-H), 8.10 (s, 1H, CH=C), 10.83 (s, 1H, OH). ^13^C-NMR (DMSO-d_6_, 100 MHz, ppm) *δ*: 150.3, 149.3 (C=O), 134.4, 134.3, 129.9, 128.5 (C–O), 127.6 (C=C), 126.1, 122.9, 116.8, 116.5, 115.8, 114.6, 114.0, 113.5, 113.4, 113.2 (C-aromatics), 112.7 (CN), 62.4 (CH_2_), 29.5 (CH_3_), 14.4 (CH_3_). Anal. calcd. For C_22_H_15_Br_2_NO_6_: C, 48.12; H, 2.75; N, 2.55. Found: C, 48.07; H, 2.55; N, 2.35.

###### Ethyl-2-cyano-3-(4-((6,8-dibromo-7-hydroxy-4-methyl-2-oxo-2*H*-chromen-3-yl)oxy)-3-methoxyphenyl)acrylate (8)

Obtained as yellow crystals, yield 79%, m.p. 230–232 °C. IR_(KBr)_*λ*_max_ (cm^−1^) = 3363 (OH), 2222 (CN), 1709 (C=O), 1580, 1507 (C=C), 1020 (C–O). ^1^H-NMR (DMSO-d_6_, 400 MHz, ppm) *δ*: 1.31–1.133 (t, 3H, CH_3_), 2.90 (s, 3H, CH_3_), 3.80 (s, 3H, OCH_3_), 4.28–4.30 (q, 2H, CH_2_), 6.70–8.02 (m, 4H, aromatic-H), 8.22 (s, 1H, CH=C), 10.50 (s, 1H, OH). ^13^C-NMR (DMSO-d_6_, 100 MHz, ppm) *δ*: 151.5, 151.2 (C=O), 150.3, 149.4, 148.2, 130.0, 128.7 (C–O), 127.8 (C=C), 127.7, 127.6, 126.3, 123.4, 117.0, 116.4, 114.6, 114.4, 114.3, 114.0 (C-aromatics), 113.5 (CN), 62.4 (CH_2_), 56.0 (OCH_3_), 31.2 (CH_3_), 14.5 (CH_3_). Anal. calcd. For C_23_H_17_Br_2_NO_7_: C, 47.70; H, 2.96; N, 2.42. Found: C, 47.60; H, 2.76; N, 2.22.

###### Ethyl-3-(3-bromo-4-((6,8-dibromo-7-hydroxy-4-methyl-2-oxo-2*H*-chromen-3-yl)oxy)-5-methoxyphenyl)-2-cyanoacrylate (11)

Obtained as brown crystals, yield 55%, m.p. 220–222 °C. IR_(KBr)_*λ*_max_ (cm^−1^) = 3291 (OH), 2227 (CN), 1729 (C=O), 1578, 1502 (C=C), 1048 (C–O). ^1^H-NMR (DMSO-d_6_, 400 MHz, ppm) *δ*: 1.20–1.29 (t, 3H, CH_3_), 2.26 (s, 3H, CH_3_), 3.78 (s, 3H, OCH_3_), 3.82–3.99 (q, 2H, CH_2_), 7.79–8.09 (m, 3H, aromatic-H), 8.20 (s, 1H, CH=C), 11.22 (s, 1H, OH). ^13^C-NMR (DMSO-d_6_, 100 MHz, ppm) *δ*: 152.4, 151.3 (C=O), 150.4, 149.6, 149.4, 148.5, 143.1 (C–O), 129.5 (C=C), 129.1, 128.7, 126.3, 123.8, 122.2, 116.1, 114.7, 113.4, 113.3, 110.2 (C-aromatics), 110.1 (CN), 56.7 (CH_2_), 53.1 (OCH_3_), 20.1 (CH_3_), 8.1 (CH_3_). Anal. calcd. For C_23_H_16_Br_3_NO_7_: C, 41.98; H, 2.45; N, 2.13. Found: C, 41.78; H, 2.35; N, 2.03.

###### Ethyl-2-cyano-3-(3,5-dibromo-4-((6,8-dibromo-7-hydroxy-4-methyl-2-oxo-2*H*-chromen-3-yl)oxy)phenyl)acrylate (12)

Obtained as pale-brown powder, yield 67%, m.p. 240–242 °C. IR_(KBr)_*λ*_max_ (cm^−1^) = 3292 (OH), 2228 (CN), 1720 (C=O), 1581, 1533 (C=C), 1089 (C–O). ^1^H-NMR (DMSO-d_6_, 400 MHz, ppm) *δ*: 1.10–1.13 (t, 3H, CH_3_), 2.10 (s, 3H, CH_3_), 3.80–3.83 (q, 2H, CH_2_), 7.90–8.10 (m, 3H, aromatic-H), 8.20 (s, 1H, CH=C), 9.90 (s, 1H, OH). ^13^C-NMR (DMSO-d_6_, 100 MHz, ppm) *δ*: 151.1, 149.3 (C=O), 148.4, 142.9, 136.6, 135.4 (C–O), 135.2 (C=C), 134.4, 134.1, 133.0, 130.8, 128.6, 126.1, 126.0, 125.7, 124.2, 123.6, 122.8 (C-aromatics), 122.3 (CN), 53.7 (CH_2_), 19.1 (CH_3_), 8.1 (CH_3_). Anal. calcd. For C_22_H_13_Br_4_NO_6_: C, 37.38; H, 1.85; N, 1.98. Found: C, 37.18; H, 1.75; N, 1.88.

##### General procedure for synthesis of novel chromen-2-yl pyrazole-carbonitrile derivatives (9 and 10)

A solution of 7 or 8 (1.2 mmol, 1 equiv.) in absolute EtOH (12 mL) was treated NH_2_NH_2_·H_2_O (3 mmol, 3 equiv.), and the resulting mixture was refluxed for 16 h. The reaction was assessed by TLC analysis, until the starting material was fully consumed. Subsequently, the reaction was cooled to room temperature and carefully quenched by ice-H_2_O. The obtained mixture was acidified with diluted HCl (1%), and the obtained crude product was isolated by filtration. The collected solid was washed with H_2_O, dried, and recrystallized using ethanol to provide the desired compounds 9 and 10.

###### 5-(4-((6,8-Dibromo-7-hydroxy-4-methyl-2-oxo-2*H*-chromen-3-yl)oxy)phenyl)-3-hydroxy-1*H*-pyrazole-4-carbonitrile (9)

Obtained as colorless crystals, yield 82%, m.p. 160–162 °C. IR_(KBr)_*λ*_max_ (cm^−1^) = 3559–3467 (br.OH), 2210 (CN), 1733 (C=O), 1542, 1508 (C=C), 1021 (C–O). ^1^H-NMR (DMSO-d_6_, 400 MHz, ppm) *δ*: 1.90 (s, 3H, CH_3_), 6.80–7.90 (m, 5H, aromatic-H), 8.50 (s, 1H, NH), 10.10 (br.s, 1H, OH), 11.60 (br.s, 1H, OH). ^13^C-NMR (DMSO-d_6_, 100 MHz, ppm) *δ*: 150.30 (C=O), 149.40 (C=N), 130.50, 129.90, 129.33, 129.10, 128.50 (C–O), 127.62, 126.10, 125.53, 125.20, 116.10, 116.03, 114.23, 113.96, 113.43, 113.33, 113.22 (C-aromatics), 112.11 (CN), 21.55 (CH_3_). Anal. calcd. For C_20_H_11_Br_2_N_3_O_5_: C, 45.06; H, 2.08; N, 7.88. Found: C, 45.02; H, 2.01; N, 7.68.

###### 5-(4-((6,8-Dibromo-7-hydroxy-4-methyl-2-oxo-2*H*-chromen-3-yl)oxy)-3-methoxyphenyl)-3-hydroxy-1*H*-pyrazole-4-carbonitrile (10)

Obtained as white crystals, yield 76%, m.p. 190–192 °C. IR_(KBr)_*λ*_max_ (cm^−1^) = 3420–3340 (br.OH), 2214 (CN), 1726 (C=O), 1592, 1548 (C=C), 1024 (C–O). ^1^H-NMR (DMSO-d_6_, 400 MHz, ppm) *δ*: 2.45 (s, 3H, CH_3_), 3.80 (s, 3H, OCH_3_), 7.02–7.92 (m, 4H, aromatic-H), 8.52 (s, 1H, NH), 9.55 (s, 2H, 2OH). ^13^C-NMR (DMSO-d_6_, 100 MHz, ppm) *δ*: 150.4 (C=O), 150.2 (C=N), 148.4, 148.2, 147.0, 140.1, 130.1, 128.4 (C–O), 127.8, 127.1, 126.3, 125.9, 123.9, 119.6, 115.9, 115.7, 114.0, 113.5 (C-aromatics), 113.1 (CN), 56.0 (CH_3_), 19.7 (CH_3_). Anal. calcd. For C_21_H_13_Br_2_N_3_O_6_: C, 44.79; H, 2.33; N, 7.46. Found: C, 44.59; H, 2.13; N, 7.26.

### 3.2 *In vitro* biological evaluations

#### 3.2.1 *In vitro* antiproliferative activity and selectivity assessment

The antiproliferative activity of the synthesized compounds was initially evaluated against the human breast adenocarcinoma cell line MCF-7 using the MTT colorimetric assay with minor modifications of a previously reported protocol.^68^ The biological profile of the lead compound 8 was subsequently expanded by evaluating its activity against the MDA-MB-231 breast cancer cell line and its cytotoxicity toward two non-cancerous cell lines, the non-tumorigenic human breast epithelial cell line MCF-10A and human lung fibroblast WI-38 cells. Cell lines were obtained from the VACSERA Cell Culture Laboratory (Giza, Egypt), originally sourced from ATCC. Cells were maintained at 37 °C in a humidified atmosphere containing 5% CO_2_ in DMEM supplemented with 10% fetal bovine serum, 1% penicillin–streptomycin, and 1% l-glutamine. For cell-viability assessment, cells were seeded into 96-well plates at a density of 5 × 10^3^ cells per well and allowed to attach overnight. Test compounds were dissolved in DMSO and diluted with complete culture medium to obtain the desired concentrations while maintaining the final DMSO concentration below 1% (v/v). Vehicle-control wells received the corresponding concentration of DMSO in complete medium without test compound, and the DMSO concentration was kept identical across all treatment and vehicle-control wells within each experiment. Doxorubicin was used as the reference drug under identical experimental conditions. Following 48 h of treatment, cell viability was determined using the MTT assay. Briefly, cells were incubated with MTT solution (20 µL, 5 mg mL^−1^ in PBS) for 3 h at 37 °C. The resulting formazan crystals were dissolved in DMSO (150 µL per well), and absorbance was measured at 570 nm using a reference wavelength of 630 nm with a BioTek ELx800 microplate reader. Cell viability was calculated relative to untreated control cells according to the following equation:Cell viability (%) = [(OD_treated_ − OD_βlank_)/(OD_control_ − OD_βlank_)] × 100

Concentration–response curves were generated using nonlinear regression analysis [log(inhibitor) *versus* normalized response, variable slope] in GraphPad Prism 10 software. The cellular IC_50_ was defined as the concentration of test compound producing a 50% reduction in MTT-derived cell viability relative to untreated control cells under the specified treatment conditions. Selectivity indices (SI) were calculated as the ratio of the cellular IC_50_ value obtained in a non-cancerous cell line (MCF-10A or WI-38) to that obtained in the corresponding breast cancer cell line (MCF-7 or MDA-MB-231). All experiments were performed in duplicate and independently repeated three times. Results are presented as mean ± SD.

#### 3.2.2 Cell cycle analysis

The effect of compound 8 on cell cycle progression was investigated by flow cytometry, performed according to the previously reported protocol with minor modifications.^69^ MCF-7 cells were seeded in 6-well plates at a density of 3 × 10^5^ cells per well and cultured overnight under standard conditions. Cells were subsequently treated with compound 8 at its IC_50_ concentration (5.9 µM) for 24 h, while vehicle-control cells received an equivalent volume of DMSO, resulting in the same final DMSO concentration (≤1%, v/v) in treated and control samples. Following treatment, cells were harvested, washed twice with cold PBS, and fixed in 70% ice-cold ethanol at −20 °C for at least 4 h. Fixed cells were washed with PBS and stained with a propidium iodide (PI)/RNase staining solution containing 50 µg mL^−1^ PI and 100 µg mL^−1^ RNase A. After incubation for 30 min at 37 °C in the dark, cellular DNA content was analyzed using a BD FACSCalibur™ flow cytometer (BD Biosciences, USA). The percentages of cells in the G_0_/G_1_, S, and G_2_/M phases were determined using ModFit LT software (version 5.0) based on DNA content distribution.

#### 3.2.3 Annexin V-FITC/PI apoptosis assay

The apoptotic effect of compound 8 on MCF-7 cells was evaluated using an Annexin V-FITC/PI apoptosis detection kit (BioVision, USA; Cat. No. K101-25) according to the previously reported protocol with minor modifications.^70^ Briefly, MCF-7 cells were seeded in 6-well plates at a density of 3 × 10^5^ cells per well and allowed to attach overnight. Cells were subsequently treated with compound 8 at its IC_50_ concentration (5.9 µM) for 24 h, while vehicle-control cells received an equivalent volume of DMSO, maintaining the same final DMSO concentration (≤1%, v/v) in both groups. Following treatment, both floating and adherent cells were collected, washed twice with cold PBS, and resuspended in Annexin V binding buffer. Cell suspensions were stained with Annexin V-FITC and PI and incubated for 15 min at room temperature in the dark. Samples were immediately analyzed using a BD FACSCalibur™ flow cytometer (BD Biosciences, USA). Fluorescence signals were detected through the FL1 channel for Annexin V-FITC and the FL2 channel for PI. Cell populations were classified as viable (Annexin V^−^/PI^−^), early apoptotic (Annexin V^+^/PI^−^), late apoptotic (Annexin V^+^/PI^+^), or necrotic (Annexin V^−^/PI^+^). Data acquisition and analysis were performed using CellQuest™ software.

#### 3.2.4 Quantitative real-time PCR analysis

The effect of compound 8 on the expression of apoptosis-related genes was investigated by quantitative real-time PCR (qRT-PCR).^71^ Briefly, MCF-7 cells were treated with compound 8 at its IC_50_ concentration (5.9 µM) for 24 h, while vehicle-control cells received an equivalent volume of DMSO, maintaining the same final DMSO concentration (≤1%, v/v). Total RNA was isolated using the RNeasy Mini Kit (Qiagen, USA) according to the manufacturer's protocol. RNA concentration and purity were assessed spectrophotometrically, and samples exhibiting *A*_260_/*A*_280_ ratios between 1.8 and 2.0 were used for subsequent analyses. Complementary DNA (cDNA) was synthesized from the purified RNA using the RT^2^ First Strand Kit (Qiagen, USA). Quantitative PCR amplification was subsequently performed on a Rotor-Gene 6000 real-time PCR system (Qiagen, USA) using RT^2^ SYBR® Green ROX™ FAST Master Mix (Qiagen, USA) and gene-specific primers targeting BAX, BCL-2, caspase-9, and p53. GAPDH was employed as the internal reference gene. Primer sequences are listed in Table S8. PCR amplification was carried out under the following cycling conditions: initial enzyme activation at 95 °C for 10 min, followed by 40 cycles of denaturation at 95 °C for 15 s and annealing/extension at 60 °C for 30 s. Melting curve analysis was performed at the end of each run to verify amplification specificity. Relative gene expression levels were calculated using the comparative 2^−ΔΔCt^ method after normalization to GAPDH expression.^72^ All experiments were performed in triplicate and independently repeated three times. Data are presented as mean ± SD, and statistical significance was considered at *p* < 0.05.

#### 3.2.5 Determination of apoptosis-related protein levels by ELISA

Protein levels of BAX, BCL-2, caspase-9, and p53 in MCF-7 cells were quantified using commercially available sandwich ELISA kits according to the respective manufacturers' instructions. MCF-7 cells were treated with compound 8 or doxorubicin at their respective cellular IC_50_ concentrations (5.9 and 14.2 µM, respectively), while vehicle-control cells received the corresponding DMSO concentration (≤1%, v/v). Following treatment, cells were harvested and lysed, and clarified cell extracts were obtained by centrifugation. BAX, BCL-2, caspase-9, and p53 were determined using the DRG® Bax ELISA Kit (EIA-4487, DRG International, USA), Zymed® Bcl-2 ELISA Kit (Cat. No. 99-0042, Invitrogen/Zymed Laboratories), DRG® Caspase-9 ELISA Kit (EIA-4860, DRG International, USA), and p53 ELISA Kit (Product No. CS0070, Sigma-Aldrich, USA), respectively.^73^ Briefly, standards and appropriately diluted cell lysates were added to antibody-coated microplate wells and incubated according to the incubation conditions specified for each kit. Following the appropriate washing steps, the respective detection antibodies and HRP-based conjugates were sequentially applied. Bound analytes were visualized using TMB substrate, and the reactions were terminated with the corresponding stop solutions. Absorbance was measured at 450 nm, with a 620 nm reference wavelength. Protein concentrations were determined by interpolation from the respective standard curves and corrected for sample dilution where applicable. BAX and p53 concentrations were expressed as pg mL^−1^, whereas BCL-2 and caspase-9 were expressed as ng mL^−1^. Experiments were independently performed three times, and data are presented as mean ± SD. Statistical differences among compound 8, doxorubicin, and vehicle-control groups were analyzed using one-way ANOVA, with *p* < 0.05 considered statistically significant.

#### 3.2.6 Determination of extracellular LDH activity

Loss of plasma-membrane integrity following treatment with compound 8 was evaluated by measuring extracellular lactate dehydrogenase (LDH) activity, as previously reported.^74^ MCF-7 cells were treated for 24 h with compound 8 or doxorubicin at their respective cellular IC_50_ concentrations (5.9 and 14.2 µM, respectively). Vehicle-control cells received an equivalent concentration of DMSO to that present in the compound 8 treatment group, and the final DMSO concentration was maintained at ≤1% (v/v). Following treatment, culture supernatants were collected and centrifuged to remove residual cells and cellular debris. Aliquots of the cleared culture supernatants and NADH standards were transferred to a clear flat-bottom 96-well plate and adjusted to a final volume of 50 µL with LDH assay buffer where necessary. Subsequently, 50 µL of freshly prepared reaction mix containing LDH assay buffer and LDH substrate mix was added to each well. Absorbance was measured at 450 nm immediately after addition of the reaction mix and subsequently monitored kinetically at 37 °C while protected from light. The change in absorbance was determined, and the amount of NADH generated was calculated from the NADH standard curve. Experiments were independently performed three times, and results are expressed as mean ± SD. Statistical differences among compound 8, doxorubicin, and vehicle-control groups were analyzed using one-way ANOVA, with *p* < 0.05 considered statistically significant.

#### 3.2.7 Assessment of microtubule polymerization inhibition

The effect of compound 8 on tubulin assembly was evaluated using a Tubulin Polymerization Assay according to the previously reported procedures.^75,76^ Briefly, purified tubulin was prepared according to the manufacturer's instructions and diluted immediately before the assay to a final concentration of 3 mg mL^−1^ in tubulin polymerization buffer (80 mM PIPES, pH 6.9, 2 mM MgCl_2_, and 0.5 mM EGTA) supplemented with 1 mM GTP and 10% glycerol and maintained on ice until use. Compound 8 was dissolved in DMSO and diluted with reaction buffer immediately before the assay. The final DMSO concentration was maintained below 1% (v/v) and was identical across all compound-treated and vehicle-control reactions. Vehicle controls contained the corresponding concentration of DMSO without compound 8. Combretastatin A4 (CA4), a colchicine-site tubulin polymerization inhibitor, was used as the reference inhibitor. Polymerization reactions were initiated by adding ice-cold tubulin reaction mixture to a pre-warmed half-area 96-well plate with a final reaction volume of 100 µL per well and immediately incubated at 37 °C. Tubulin assembly was monitored kinetically by measuring absorbance at 340 nm using a temperature-controlled microplate reader. The extent of polymerization inhibition was determined relative to vehicle-treated controls, and concentration–response curves were generated by nonlinear regression using GraphPad Prism software to determine the corresponding IC_50_ values. All experiments were performed in triplicate, and the results are presented as mean ± SD.

#### 3.2.8 Evaluation of EGFR tyrosine kinase inhibitory activity

The inhibitory activity of compound 8 against epidermal growth factor receptor (EGFR) tyrosine kinase was determined using a commercially available ELISA-based kinase assay according to a previously reported protocol with minor modifications.^69^ Briefly, purified EGFR kinase and poly(Glu,Tyr) substrate were incubated in the presence of various concentrations of compound 8 in kinase reaction buffer containing ATP. Compound 8 was introduced from a DMSO stock solution, and the final DMSO concentration was kept constant across all treatment concentrations and the corresponding vehicle-control reactions (≤1%, v/v); vehicle controls contained the same concentration of DMSO without compound 8. Erlotinib was used as the reference EGFR inhibitor under identical experimental conditions. Following incubation at 37 °C, substrate phosphorylation was quantified using an anti-phosphotyrosine monoclonal antibody conjugated to horseradish peroxidase (HRP). Color development was achieved by addition of the chromogenic substrate, and absorbance was measured using a Tecan Spark microplate reader. The percentage inhibition of EGFR activity was calculated relative to untreated controls, and concentration–response curves were generated by nonlinear regression analysis to determine the corresponding IC_50_ values. All measurements were performed in triplicate, and results are expressed as mean ± SD.

### 3.3 *In silico* molecular modelling analysis

Molecular docking studies were performed to investigate the binding modes of compound 8 within the colchicine-binding site of tubulin and the ATP-binding pocket of EGFR. The crystal structures of the tubulin αβ-heterodimer co-crystallized with combretastatin A4 (CA4) (PDB ID: 5LYJ) and the EGFR tyrosine kinase domain co-crystallized with Erlotinib (AQ4) (PDB ID: 1M17) were retrieved from the Protein Data Bank. For the tubulin model (5LYJ), co-crystallized accessory proteins (TTL and stathmin-like domain), redundant tubulin chains, water molecules, and non-essential heteroatoms were removed. In the EGFR structure (1M17), water molecules were removed while the co-crystallized ligand was retained for docking protocol validation. Polar hydrogens were added to all protein structures using PyMOL. Docking scores and CNN-derived metrics were interpreted comparatively as computational descriptors of the generated poses and were not considered direct measurements of experimental binding affinity. The predicted binding modes were therefore used to generate structural hypotheses rather than to establish definitive binding geometries or quantitative affinity relationships. Compound 8 was prepared from its SDF structure using Open Babel. Ligand preparation included geometry optimization, addition of polar hydrogens at physiological pH (7.4), and assignment of Marsili–Gasteiger partial charges. The co-crystallized ligands, CA4 and Erlotinib, were prepared using the same protocol and subsequently employed for docking validation. Docking calculations were performed using Smina, an enhanced implementation of the AutoDock Vina scoring function. The docking protocol was first validated by redocking the native ligands into their corresponding binding sites using grids automatically generated around the crystallographic ligands through the autobox_ligand option, in line with standard preparation workflows reported in earlier studies.^77–79^ Docking accuracy was assessed by calculating the root-mean-square deviation (RMSD) between the crystallographic and redocked poses and by evaluating the conservation of key protein–ligand interactions. Following protocol validation, compound 8 was docked into the colchicine-binding pocket of tubulin and the ATP-binding site of EGFR using the validated Smina docking workflow. For the tubulin system, a semi-flexible receptor protocol was additionally employed, as the comparatively large molecular scaffold of compound 8 required accommodation within the spatially constrained colchicine-binding cavity. Receptor flexibility was introduced according to the implemented semi-flexible protocol. In contrast, the EGFR ATP-binding site was treated using the standard rigid-receptor protocol because the crystallographic binding cavity provided sufficient space for initial placement of compound 8 without introducing additional receptor side-chain flexibility. For both targets, docking searches were performed using the default Smina exhaustiveness setting. No positional constraints, pharmacophore restraints, hydrogen-bond constraints, or other user-defined spatial constraints were imposed. No additional enhanced-sampling procedures were applied beyond the receptor flexibility explicitly introduced for the tubulin model. Docking poses were rescored using Gnina, which combines the AutoDock Vina empirical scoring function with a convolutional neural network (CNN)-based scoring algorithm for pose evaluation and affinity prediction. Binding affinities, CNN scores, intermolecular and intramolecular energy contributions, hydrophobic interactions, hydrogen-bonding terms, and steric repulsion penalties were extracted using the score_only mode. Two-dimensional protein–ligand interaction diagrams were generated using BIOVIA Discovery Studio Visualizer, while three-dimensional structural representations were prepared using PyMOL.

### 3.4 *In vivo* antitumor evaluation and mechanistic investigations

#### 3.4.1 Animals and ethical statement

Adult female Swiss albino mice (20–25 g) were obtained from the Animal House of the National Research Centre (NRC), Dokki, Giza, Egypt. Animals were housed in stainless-steel cages under standard laboratory conditions, including a controlled temperature of 22 ± 2 °C and a 12 h light/dark cycle. Standard laboratory chow and water were provided *ad libitum* throughout the experimental period. Prior to the initiation of the study, animals were acclimatized for seven days under the same environmental conditions. Animals were monitored daily for signs of distress, illness, or abnormal behavior throughout the study. All experimental procedures were approved by the Animal Ethics Committee of the Faculty of Science, Port Said University, Egypt (Approval No. ERN: PSU.Sci.8) and were conducted in accordance with the guidelines for the care and use of laboratory animals and the relevant institutional regulations.

#### 3.4.2 Assessment of tumor burden in EAC-bearing mice

EAC cells were obtained from the National Cancer Institute (Cairo, Egypt) and maintained by serial intraperitoneal transplantation in female Swiss albino mice at 8–10 days intervals. At the end of the experimental period, ascitic fluid was collected aseptically from the peritoneal cavity of each animal using a graduated syringe, and the total ascites volume was recorded. To determine viable tumor cell counts, aliquots of the collected ascitic fluid were diluted with heparinized saline and stained with trypan blue solution. Cell viability and density were assessed using the trypan blue exclusion method and counted with a Neubauer hemocytometer under light microscopy. Viable cells excluded the dye and remained unstained, whereas non-viable cells exhibited blue staining. The total number of viable EAC cells was calculated and expressed as ×10^6^ cells per mL.^67^ All measurements were performed in triplicate, and the results are presented as mean ± SD.

#### 3.4.3 Acute toxicity evaluation and dose selection

The acute toxicity profile of compound 8 was evaluated in female Swiss albino mice according to the method of Meier and Theakston, with minor modifications.^80^ Animals were randomly assigned to dose groups (*n* = 3 per group) and administered compound 8 intraperitoneally at progressively increasing doses ranging from 1 to 300 mg kg^−1^. Control animals received an equivalent volume of sterile saline. Animals were monitored for up to 48 h for mortality and observable signs of acute toxicity, including alterations in locomotor activity, grooming behavior, food consumption, and general appearance. During the initial range-finding phase, doses of 1–10 mg kg^−1^ were evaluated, followed by escalation to 25, 50, 100, 200, and 300 mg kg^−1^ using separate groups of animals. No mortality or observable signs of acute toxicity were detected at doses up to 200 mg kg^−1^. In contrast, administration of 300 mg kg^−1^ resulted in pronounced lethargy, reduced locomotor activity, exhaustion, and mortality (Table S9). Accordingly, 200 mg kg^−1^ was considered the highest tested dose without observed acute toxicity under the conditions of this preliminary range-finding study. Based on these findings, doses of 5, 10, and 20 mg kg^−1^ were selected for the subsequent antitumor efficacy study, corresponding to 2.5%, 5%, and 10%, respectively, of the highest tested non-toxic dose.

#### 3.4.4 Experimental design and treatment regimen in EAC-bearing mice

The *in vivo* antitumor activity of compound 8 was evaluated using an EAC-mouse model over an 14 days experimental period. Thirty-six female Swiss albino mice were randomly assigned to six groups (*n* = 6/group). Group I served as the normal control and received sterile 0.9% saline intraperitoneally on alternate days throughout the study. Group II received compound 8 (20 mg kg^−1^, i.p.) on alternate days for five administrations in the absence of EAC inoculation to evaluate any treatment-related effects in healthy animals. Group III served as the untreated tumor-bearing control and was inoculated intraperitoneally with 2.5 × 10^6^ viable EAC cells suspended in 200 µL sterile saline on day 1. Groups IV–VI were similarly inoculated with EAC cells and subsequently treated with compound 8 at doses of 5, 10, and 20 mg kg^−1^, respectively. Treatment commenced 24 h after tumor inoculation (day 2) and was administered intraperitoneally on alternate days for a total of five doses. At the end of the experimental period, animals were anesthetized using a ketamine/xylazine regimen and euthanized in accordance with institutional ethical guidelines.^81^ Ascitic fluid was collected from the peritoneal cavity for the determination of tumor cell viability and count. Blood samples were obtained and centrifuged at 3000 rpm for 10 min to separate serum for biochemical analyses. Liver and kidney tissues were excised immediately after sacrifice; a portion of each organ was stored at −20 °C for preparation of tissue homogenates, while the remaining portion was fixed in 10% neutral buffered formalin for histopathological and immunohistochemical examinations.

#### 3.4.5 Assessment of serum biochemical markers of hepatic and renal function

Serum biochemical parameters were determined to evaluate the effects of compound 8 on hepatic and renal functions in EAC-bearing mice. Hepatic injury was assessed by measuring serum ALT and AST activities, as previously reported.^82,83^ Briefly, both assays are based on enzymatic transamination reactions coupled to NADH oxidation, whereby the decrease in absorbance at 340 nm is directly proportional to enzyme activity. Serum samples were incubated with the corresponding reaction reagents, and absorbance was measured spectrophotometrically using a microplate reader. Enzyme activities were calculated from calibration standards and expressed as U L^−1^. Renal function was evaluated by determining serum creatinine and urea concentrations, as previously reported.^81,84^ Creatinine levels were quantified through an enzymatic colorimetric reaction based on the generation of hydrogen peroxide, which subsequently reacts with a chromogenic substrate to produce a colored product measurable at 510 nm. Urea concentration was determined using an enzymatic assay in which the generated chromophore was measured at 340 nm. Concentrations were calculated from the corresponding standard curves and expressed as mg dL^−1^. All measurements were performed in triplicate, and the results are presented as mean ± SD and were subjected to statistical analysis as described under Statistical analysis.

#### 3.4.6 Assessment of hepatic oxidative stress and antioxidant biomarkers

To evaluate the impact of compound 8 on hepatic redox homeostasis, liver homogenates were analyzed for malondialdehyde (MDA), reduced glutathione (GSH), catalase (CAT), and superoxide dismutase (SOD), as previously reported.^85–88^ Briefly, liver tissues were homogenized in ice-cold buffer and centrifuged to obtain clear supernatants. Aliquots of the resulting homogenates were added to antibody-coated microplates. Following sequential incubation with the corresponding detection reagents and horseradish peroxidase (HRP)-conjugated antibodies, color development was achieved using tetramethylbenzidine (TMB) substrate. The enzymatic reaction was terminated with stop solution, and absorbance was measured at 450 nm using a microplate reader. Biomarker concentrations or enzymatic activities were calculated from the corresponding standard curves and normalized to tissue weight where appropriate. All measurements were performed in triplicate, and results are presented as mean ± SD, and were subjected to statistical analysis as described under Statistical analysis.

#### 3.4.7 Assessment of serum inflammatory markers

To investigate the anti-inflammatory effect of compound 8 in EAC-bearing mice, levels of serum interleukin-6 (IL-6) and p38 mitogen-activated protein kinase (p38 MAPK) were quantified, as previously reported.^89,90^ Briefly, appropriate dilutions of serum and standard solutions were added to antibody-precoated microplates. Following sequential incubation with biotinylated detection antibodies and horseradish peroxidase (HRP)-conjugated reagents, color development was achieved using tetramethylbenzidine (TMB) substrate. The enzymatic reaction was terminated using stop solution, and absorbance was measured at 450 nm using a microplate reader. The concentrations of IL-6 and p38 MAPK were calculated from the corresponding standard curves and expressed as pg mL^−1^ and ng mL^−1^, respectively. All samples were analyzed in triplicate, and results are presented as mean ± SD, and were subjected to statistical analysis as described under Statistical analysis.

#### 3.4.8 Histopathological examination

Liver and kidney specimens were collected immediately after sacrifice and fixed in 10% neutral buffered formalin. Fixed tissues were processed through routine dehydration, cleared, embedded in paraffin wax, and sectioned at 5 µm thickness using a rotary microtome. Sections were mounted on glass slides, deparaffinized, rehydrated, and stained with hematoxylin and eosin (H&E) for routine histopathological assessment. Liver sections were examined for preservation of hepatic architecture and the presence of steatosis, hepatocellular ballooning, lobular inflammation, necrotic changes, and fibrosis. Hepatic lesions were semi-quantitatively scored using a modified Brunt scoring system. Kidney sections were evaluated for glomerular integrity, tubular epithelial changes, interstitial inflammation, tubular atrophy, and interstitial fibrosis. Renal injury was assessed semi-quantitatively, with particular attention to features of acute tubular injury, including tubular epithelial flattening, cytoplasmic vacuolization, loss of brush border, and tubular necrosis. Microscopic examination and image acquisition were performed using a Nikon Eclipse E800 microscope equipped with a digital camera.^91,92^

#### 3.4.9 Immunohistochemical analysis of IL-6 and p38 MAPK

Immunohistochemical staining was performed on selected paraffin-embedded liver sections to evaluate hepatic expression of IL-6 and p38 MAPK. Sections of 4 µm thickness were cut, mounted on positively charged slides, deparaffinized, and rehydrated through descending grades of ethanol. Antigen retrieval was performed using citrate buffer, followed by blocking of endogenous peroxidase activity and non-specific binding sites. Sections were incubated with primary antibodies against IL-6 and p38 MAPK. After washing, slides were incubated with the appropriate HRP-conjugated secondary antibody. Immunoreactivity was visualized using 3,3′-diaminobenzidine as the chromogenic substrate, and sections were counterstained lightly with hematoxylin, dehydrated, cleared, and mounted. Stained sections were examined under light microscopy, and cytoplasmic immunoreactivity in hepatocytes was semi-quantitatively evaluated using a modified Allred scoring system based on the percentage of positively stained cells and staining intensity. The final immunoreactivity score was calculated by combining the percentage and intensity scores.^71,78^

#### 3.4.10 Statistical analysis

Statistical analyses were performed using GraphPad Prism software (version 10.4.1; GraphPad Software, San Diego, CA, USA). Data were initially assessed for normality using the Shapiro–Wilk test. Comparisons among multiple groups were conducted using one-way analysis of variance (ANOVA) followed by Tukey's multiple-comparison post hoc test. Data are presented as mean ± SD. All experiments were performed in triplicate. Statistical significance was considered at *p* < 0.05 and represented as follows *p ≤ 0.05, **p ≤ 0.01, ***p ≤ 0.001, ****p ≤ 0.0001.

#### 3.4.11 Ethics approval

All experimental procedures were conducted in accordance with the guidelines of the Institutional Animal Care and Use Committee (IACUC) of Port Said University, Egypt. The study protocol was reviewed and approved by the Research Ethics Committee for Experimental and Clinical Studies, Faculty of Science, Port Said University (Approval No. ERN: PSU.Sci.8). Efforts were made to minimize animal suffering and to reduce the number of animals used.

## 4. Conclusion

In summary, the present study highlighted the utility of the 6,8-dibromo-7-hydroxy-4-methylcoumarin scaffold as a versatile platform for the development of biologically active compounds with promising antiproliferative properties against breast cancer cells. Strategic incorporation of cyanoacrylate pharmacophore generated a series of structurally diverse hybrids with distinct biological profiles, revealing important SAR that guided the identification of compound 8 as the most promising lead candidate. Extended cellular profiling indicated that compound 8 retained antiproliferative activity in both MCF-7 and MDA-MB-231 breast cancer cells while exhibiting lower antiproliferative activity toward the non-cancerous MCF-10A and WI-38 cell lines. Comprehensive mechanistic investigations revealed that the *in vitro* antiproliferative activity of compound 8 is mediated through the simultaneous modulation of multiple cancer-associated pathways. The compound effectively disrupted cell-cycle progression, inhibited tubulin polymerization, and suppressed EGFR activity, indicating interference with both mitotic machinery and proliferative signaling networks. These effects were accompanied by significant activation of the intrinsic apoptotic pathway, supported by enhanced Annexin V-positive apoptotic cell death and concordant transcriptional and protein-level modulation of key apoptotic regulators, including increased p53, BAX, and caspase-9 and suppression of the anti-apoptotic protein BCL-2. Importantly, the promising *in vitro* profile of compound 8 translated into significant *in vivo* antitumor efficacy in the EAC model. Treatment markedly reduced tumor burden and viable tumor cell counts while simultaneously ameliorating EAC-associated hepatic and renal dysfunction. Furthermore, compound 8 effectively restored redox homeostasis through attenuation of lipid peroxidation and enhancement of endogenous antioxidant defenses. The observed suppression of IL-6 and p38 MAPK signaling, together with the substantial reduction in hepatic p38 immunoreactivity, highlights the ability of the compound to interfere with inflammation-driven tumor progression. These biochemical findings were further corroborated by histopathological examination, which demonstrated considerable restoration of normal liver and kidney architecture and attenuation of tumor-associated tissue injury. Taken together, these findings identify compound 8 as a promising anticancer lead and support the 6,8-dibromo-7-hydroxy-4-methylcoumarin scaffold as a basis for further medicinal-chemistry optimization. Nevertheless, the relatively small number of analogues examined in the present study limits the establishment of a comprehensive SAR, and the identified structural trends require validation through expansion of the chemical series. In addition, the use of the EAC ascites model represents a limitation with respect to breast-cancer-specific translation, as this model does not reproduce the anatomical and microenvironmental characteristics of an orthotopic solid breast tumor. Future studies should focus on further optimization of this scaffold, detailed pharmacokinetic and safety investigations, validation in other clinically relevant breast cancer models, and target validation studies to facilitate the translation of these findings toward the development of novel multifunctional anticancer therapeutics.

## Author contributions

Conceptualization, M. A. E., I. M. E., Z. A., and E. M. S.; methodology, M. A. E., I. M. E., Z. A., I. M. A., S. Z. A., and E. M. S.; software, M. A. E., Z. A., I. M. A., S. Z. A., and E. M. S.; validation, M. A. E., I. M. E., Z. A., I. M. A., and E. M. S.; formal analysis, M. A. E., I. M. E., Z. A., and E. M. S.; investigation, M. A. E., I. M. E., I. M. A., S. Z. A., and E. M. S.; resources, M. A. E., I. M. E., Z. A., I. M. A., S. Z. A., and E. M. S.; data curation, M. A. E., I. M. E., Z. A., and E. M. S.; writing—original draft preparation M. A. E., I. M. E., Z. A., I. M. A., S. Z. A., and E. M. S.; writing—review and editing, M. A. E., I. M. E., Z. A., I. M. A., S. Z. A., and E. M. S.; visualization, M. A. E., I. M. E., Z. A., and E. M. S.; supervision, M. A. E., I. M. E., and E. M. S.; project administration, M. A. E., I. M. E., Z. A., S. Z. A., and E. M. S.; funding acquisition, M. A. E., I. M. E., Z. A., I. M. A., S. Z. A., and E. M. S. All authors have read and agreed to the published version of the manuscript.

## Conflicts of interest

The authors affirm that they are not aware of any personal or financial conflicts that would have seemed to affect the findings of this study's research.

## Supplementary Material

RA-016-D6RA06101J-s001

## Data Availability

Data supporting the results reported in this manuscript are included in this article and as part of the supplementary information (SI). The raw data supporting the conclusions of this article will be made available by the authors without any undue reservation. Samples of final compounds are available from the authors upon request. Supplementary information is available. See DOI: https://doi.org/10.1039/d6ra06101j.
