# Supplementary material for: Development of 7-hydroxy-4-methylcoumarin–cyanoacrylate hybrids with antiproliferative activity against breast cancer cells: from molecular design to in vivo validation
Source: RSC Adv. 2026 Oct 27. Online ahead of print. doi: 10.1039/d6ra06101j (PMC13712327; doi:10.1039/d6ra06101j)
Supplement: Supplementary file 1 [file RA-OLF-D6RA06101J-s001.pdf]

Supplementary Information (SI) for RSC Advances.
This journal is © The Royal Society of Chemistry

Supporting Information

Table S1: Selectivity indices of compound **8** and DOXO across breast cancer and non-cancerous cell-line pairs

Cancer cell line	Non-cancerous cell line	Compound 8 SI	DOXO SI
**MCF-7**	MCF-10A	7.81	1.70
**MCF-7**	WI-38	6.34	1.11
**MDA-MB-231**	MCF-10A	3.93	12.12
**MDA-MB-231**	WI-38	3.20	7.98

Table S2. Protein–ligand interactions observed for the redocked poses of combretastatin A4 and compound **8** within the colchicine-binding site of tubulin (PDB ID: 5LYJ)

Interaction type	Comb A4	Comp8
**Conventional hydrogen bond**	-	B:ASN167 (2.35) B:ASP251 (2.84) A:THR179 (2.22)
**Carbon Hydrogen Bond**	B:ASN258 (3.45) B:ALA317 (3.48) B:LYS352(3.35)	B:VAL238 (3.31)
**Pi-Sigma**	B:LEU255(3.88)	-
**Pi-Sulfur**	B:MET259(5.1)	-
**Alkyl**	B:ALA316 (3.79) B:MET259 (4.71) B:LYS352 (5.01) B:ILE318 (4.39) B:ILE378 (4.53) B:LEU242 (4.75) B:LEU255 (3.94)	B:LEU248(4.45) B:LYS254(4.15) B:MET259(5.30) B:LYS352(3.64) B:CYS241(4.07) B:ILE318(4.78) B:ILE378(5.14) B:ILE4(3.86) B:LEU242(4.81) B:LEU252(3.76)
**Pi-Alkyl**	B:CYS241 (4.15) B:ALA250 (4.25) B:ALA316 (4.60) B:ALA316 (5.10) B:LYS352 (4.51)	B:LEU248(4.87) B:ALA316(5.21) B:LYS352(4.55) B:CYS241(5.49) B:LEU242(4.81) B:ALA250(5.00) B:LEU255(3.48)
**Amide-Pi Stacked**	B:ASN258;MET259:N (5.08)	-
**Pi-Cation**	-	B:LYS352 (4.87)

*Interaction distances are expressed in Å and correspond to the lowest-energy docking pose

Table S3: Comparative docking scores and energetic contributions for the redocked CA4, and compound **8** within the colchicine-binding site of tubulin (PDB ID: 5LYJ)

Metric / Ligand	Comb A4 (Redocked)	Comp8 (Flexible Pose)
**Gnina Affinity (ΔG)**	-8.78 kcal/mol	-5.35 kcal/mol
**CNNscore**	0.666	0.645
**Intramolecular Energy**	-0.839 kcal/mol	+1.180 kcal/mol
**Repulsion Penalty**	+1.66 kcal/mol	+14.57 kcal/mol
**Gauss 1 (kcal/mol)**	-3.68	-5.55
**Gauss 2 (kcal/mol)**	-8	-11.81
**Hydrophobic (kcal/mol)**	-1.48	-2.9
**H-Bonds (kcal/mol)**	-0.36	-2.17
**Intermolecular Energy**	-11.86	-7.86
**Flex Divisor**	~1.351	~1.467

Table S4. Comparative docking metrics and interaction fingerprints of three selected CNN-ranked poses of compound **8** within the colchicine-binding region of tubulin (PDB ID: 5LYJ).

Rank pose	Smina affinity (kcal/mol)	CNNscore	CNNaffinity	CNN variance	Intramolecular energy (kcal/mol)	Principal H-bond interactions	Principal hydrophobic / other interactions
**1**	−5.207	0.318	6.758	0.399	+1.422	C–H···O contacts with VAL238 and ASP251	LEU248, MET259, LYS352, CYS241, ALA250, LEU255, ALA316, VAL181; halogen contact with ASN349; π-sulfur interaction with MET259
**2**	−5.510	0.149	5.633	0.513	+1.226	LYS352 (2.98 Å), THR353 (2.45 Å), GLN176/Br contact (1.98 Å); additional C–H···O contacts with ALA354, PRO175 and GLN176	LEU333, MET325, ALA354, PRO175, CYS241, LEU248; π-sulfur with MET325 and π-lone-pair interaction with THR353
**3**	−5.673	0.122	5.261	0.549	+1.467	GLN176 (2.43 Å), GLN247 (2.06 Å), LEU248 (2.38 Å), ASP329 (2.66 Å)	CYS241, CYS356, TYR210, LEU248, ALA354; π-anion interaction with ASP329

The generated poses showed substantial variability in both classical docking scores and CNN-based pose-confidence metrics. Several residues, including CYS241, LEU248, LYS352, and residues within the MET325–VAL355 region, participated recurrently in interactions across alternative poses. Nevertheless, the variation in pose geometry and interaction patterns emphasizes that the docking results should be interpreted as alternative structural hypotheses rather than evidence for a uniquely defined binding orientation.

Table S5. Protein–ligand interactions observed for the redocked AQ4, and compound **8** within the ATP-binding site of EGFR (PDB ID: 1M17)

Interaction type	Erlotinib redocked	Comp8
**Hydrogen bond**	A:CYS773(3.07)	A:MET769(2.05)
**Carbon hydrogen bond**	A:GLN767(3.54)	A:MET769(2.47)
**Pi-cation**	A:LYS721(4.35)	
**Pi-anion**		A:ASP776(4.36)
**Pi-sigma**	A:LEU694(3.76)<br>A:LEU820(3.78)	
**Pi-alkyl**	A:ALA719(3.89)<br>A:VAL702(5.29)<br>A:LEU820(5.38)<br>A:LYS721(4.53)	A:TYR777(5.37)<br>A:LEU694(4.66)
**Alkyl**	A:LYS721(4.33)<br>A:MET742(5.22)<br>A:LEU764(3.72)	A:LEU694(4.21)<br>A:LEU768(4.56)<br>A:VAL702(3.82)

*Interaction distances are expressed in Å and correspond to the lowest-energy docking pose

Table S6. Comparative docking scores and energetic contributions for the redocked AQ4, and compound **8** within the ATP-binding site of EGFR (PDB ID: 1M17)

Metric / Ligand	Erlotinib	Comp8
**Gnina Affinity (ΔG)**	-7.14 kcal/mol	-7.88 kcal/mol
**CNNscore**	0.898	0.416
**Intramolecular Energy**	-1.080 kcal/mol	-0.745 kcal/mol
**Repulsion Penalty**	+1.32 kcal/mol	+1.29 kcal/mol
**Gauss 1 (kcal/mol)**	-3.12	-3.23
**Gauss 2 (kcal/mol)**	-7.84	-7.48
**Hydrophobic (kcal/mol)**	-1.45	-1.02
**H-Bonds (kcal/mol)**	-0.63	-1.13
**Intermolecular Energy**	-11.73	-11.57
**Flex Divisor**	~1.643	~1.468

Table S7. Comparative docking metrics and interaction fingerprints of three selected CNN-ranked poses of compound **8** within the ATP-binding site of EGFR (PDB ID: 1M17).

Rank pose	Smina affinity (kcal/mol)	CNNscore	CNNaffinity	CNN variance	Intramolecular energy (kcal/mol)	Principal H-bond/polar interactions	Principal hydrophobic/other interactions
**2**	−7.829	0.158	6.067	0.201	+0.510	CYS773 (2 contacts), THR766; C–H···O contacts with GLY772 and PHE771	VAL702, LEU694, LEU820, LYS721, MET742, CYS773, PHE699; halogen contact with GLU738; π-anion interaction with ASP831
**3**	−7.834	0.124	6.047	0.309	−0.638	C–H···O contact with PHE771	LEU694, VAL702, LEU820, PHE699; π-sulfur interaction with CYS773
**4**	−7.864	0.097	6.113	0.591	−0.402	LYS721 (2.38 Å), CYS773 (2.26 Å), THR830 (2.04 Å); C–H···O contacts with ARG817 and THR766	LEU694, CYS773, LYS721, VAL702, LEU820

Alternative poses displayed partially overlapping interaction fingerprints, with recurrent contacts involving LEU694 and VAL702 and, in several orientations, CYS773 and LEU820. The variability in hydrogen-bonding patterns among the alternative solutions indicates that multiple orientations can be accommodated within the modeled ATP-binding cavity and further supports interpretation of the docking results as structural hypotheses rather than a uniquely established binding mode.

Table S8. List of primer sequences utilized in this study.

Gene	Primer sequence
P53	F 5'- CCTCAGCATCTTATCCGAGTGG -3',
	R 5'- TGGATGGTGGTACAGTCAGAGC -3'.
BAX	F 5'- TCAGGATGCGTCCACCAAGAAG -3',
	R 5'- TGTGTCCACGGCGGCAATCATC -3'.
Bcl-2	F 5'- ATCGCCCTGTGGATGACTGAGT-3',
	R 5'- GCCAGGAGAAATCAAACAGAGGC-3'
Caspase 9	F 5'- GTTTGAGGACCTTCGACCAGCT -3'
	R 5'- CAACGTACCAGGAGCCACTCTT -3'
GAPDH	F 5'- GTCTCCTCTGACTTCAACAGCG-3'
	R 5'- ACCACCCTGTTGCTGTAGCCAA-3'

Table S9. Acute toxicity range-finding profile of compound **8** following intraperitoneal administration in female Swiss albino mice.

Dose tested (mg/kg)	n per dose	Observation period	Observed findings	Mortality	Interpretation
1–10	3	Up to 48 h	No observable behavioral or systemic signs of acute toxicity	0/3 per dose	Tolerated under the study conditions
25	3	48 h	No observable signs of acute toxicity	0/3	Tolerated
50	3	48 h	No observable signs of acute toxicity	0/3	Tolerated
100	3	48 h	No observable signs of acute toxicity	0/3	Tolerated
200	3	48 h	No observable signs of acute toxicity	0/3	Highest tested dose without observed acute toxicity
300	3	48 h	Pronounced lethargy, reduced locomotor activity, exhaustion, followed by mortality	2/3	Toxic/lethal dose under the study conditions

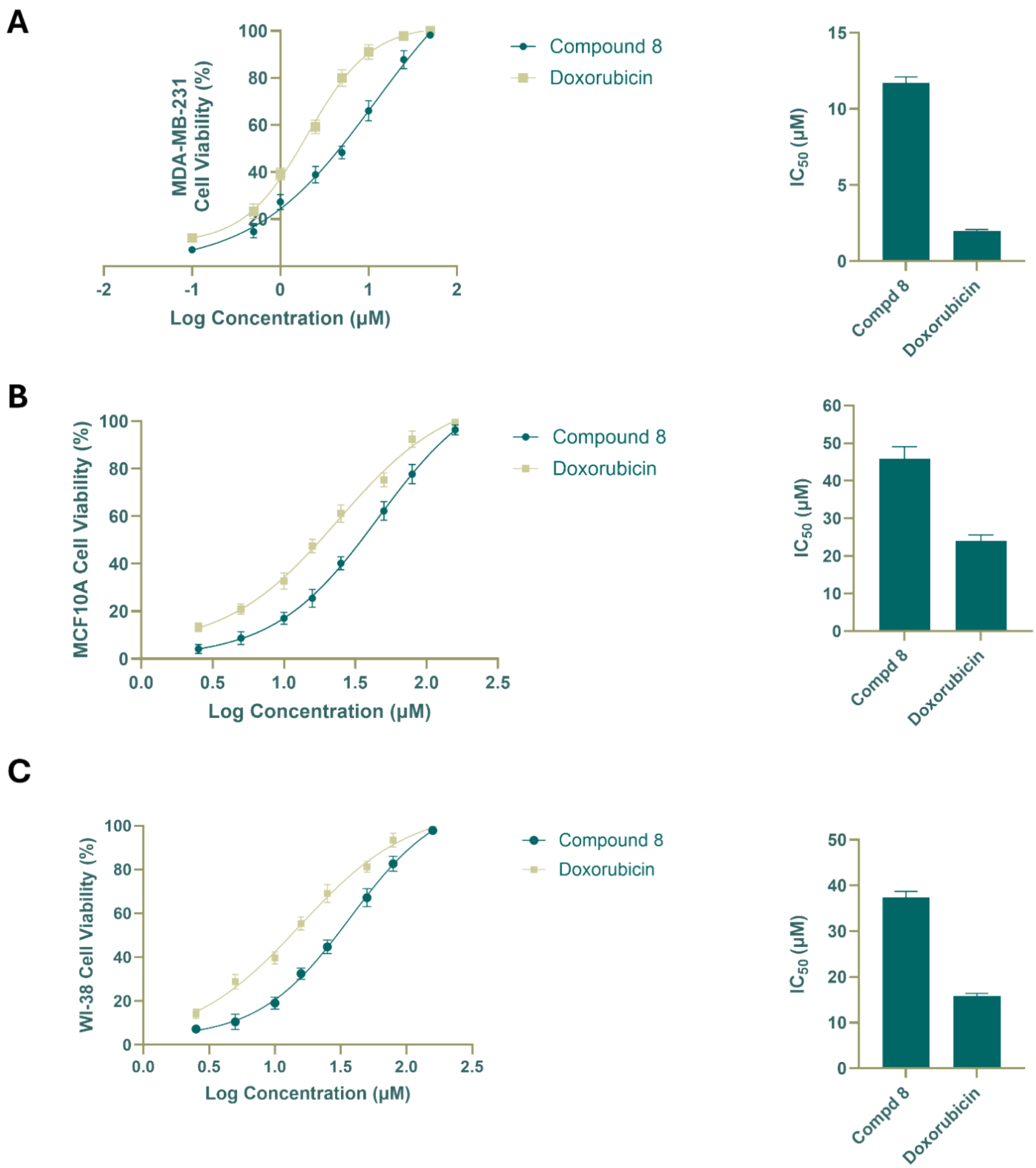

Figure S1: *In-vitro* antiproliferative and selectivity profile of compound **8**. Concentration-response curves and corresponding cellular IC₅₀ values for compound **8** and doxorubicin following 48 h treatment of (**A**) MDA-MB-231 breast cancer cells, (**B**) non-tumorigenic MCF-10A breast epithelial cells, and (**C**) non-cancerous WI-38 fibroblasts. Cell viability was determined by MTT assay, and IC₅₀ values were calculated by nonlinear regression using a log(inhibitor) versus normalized response model. Data are presented as mean ± SD from three independent experiments (n = 3).

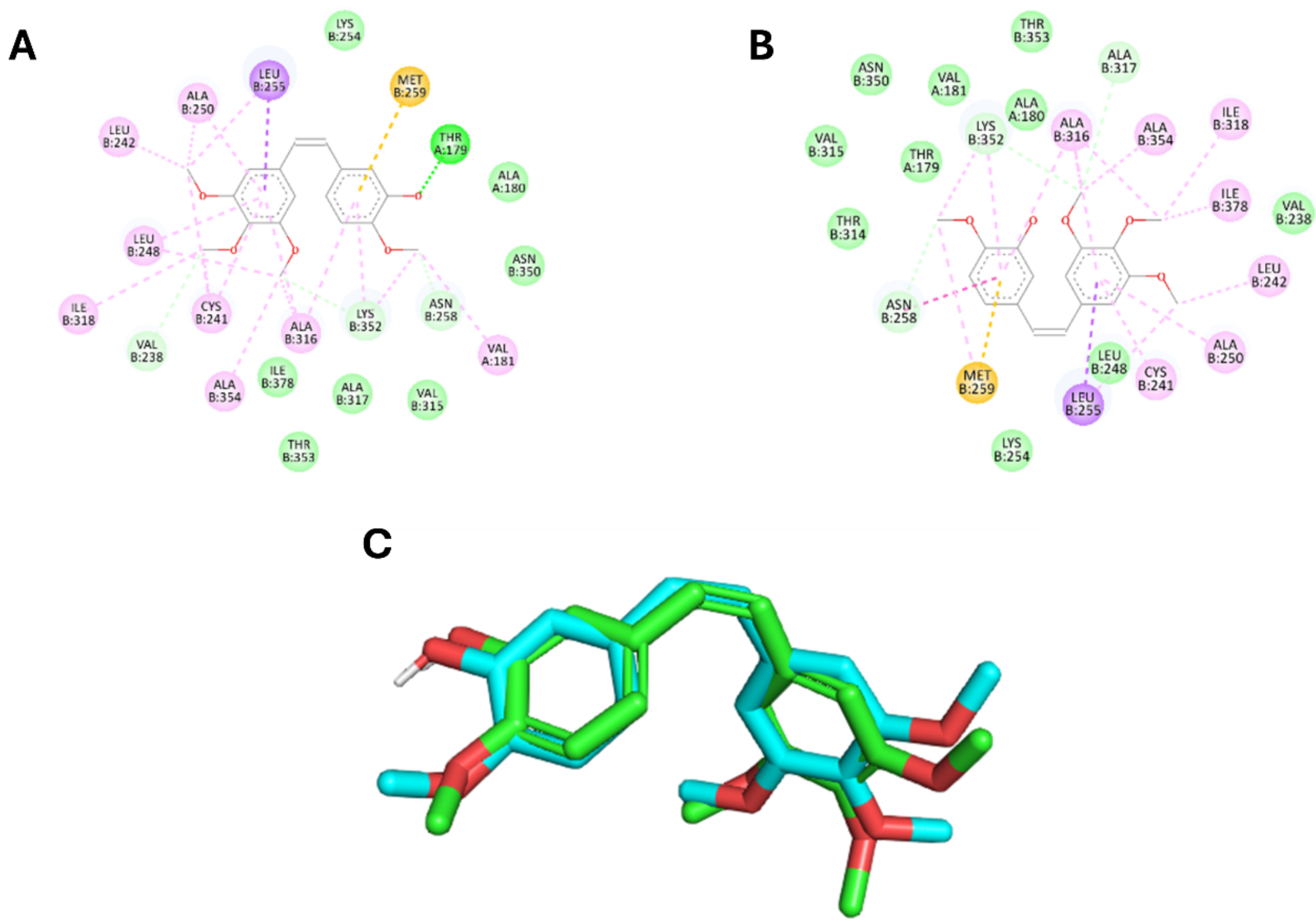

Figure S2. Validation of the molecular docking protocol for the colchicine-binding site of tubulin (PDB ID: 5LYJ). (A) Two-dimensional interaction map of the co-crystallized ligand, combretastatin A4 (CA4), within the colchicine-binding pocket of tubulin. **(B)** Two-dimensional interaction map of the redocked CA4 pose showing the reproduced protein–ligand interactions within the binding site. **(C)** Superposition of the co-crystallized CA4 pose (cyan) and the redocked pose (green) within the colchicine-binding pocket, demonstrating successful reproduction of the experimental binding mode (RMSD = 0.777 Å).

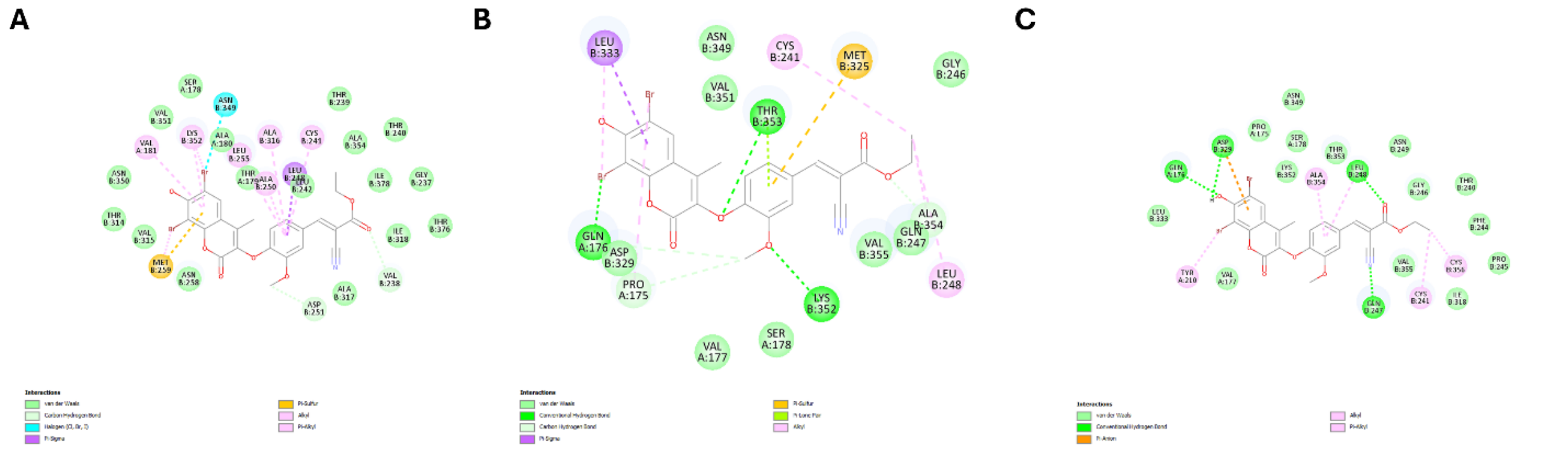

Figure S3. Two-dimensional interaction maps of alternative docking poses of compound 8 within the colchicine-binding region of tubulin (PDB ID: 5LYJ). (**A**) Pose 2, (**B**) Pose 3, and (**C**) Pose 4, corresponding to the second-, third-, and fourth-highest CNN-ranked docking solutions, respectively. The diagrams depict the predicted hydrogen-bonding, hydrophobic, π-mediated, halogen, and other non-covalent interactions between compound **8** and residues lining the colchicine-binding cavity. The highest CNN-ranked pose (Pose 8) is presented in Figure 3C of the main manuscript. Detailed docking scores, CNN metrics, and interaction fingerprints for all four selected poses are summarized in **Table S4**.

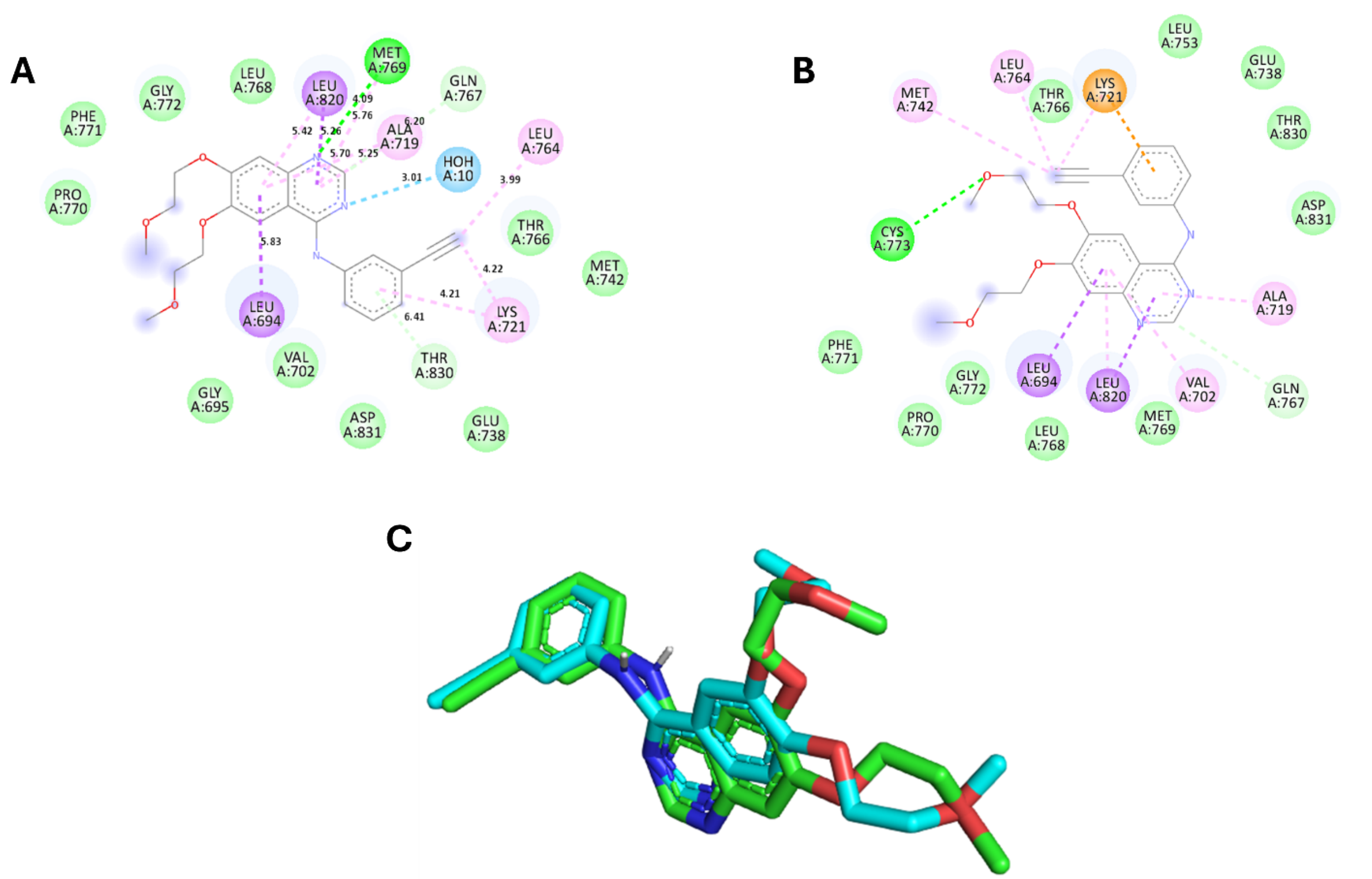

Figure S4. Validation of the molecular docking protocol through redocking of AQ4 into the ATP-binding site of EGFR (PDB ID: 1M17). (A) Two-dimensional protein–ligand interaction map of the crystallographic AQ4 pose within the EGFR active site. **(B)** Two-dimensional interaction map of the redocked AQ4 pose showing the reproduced binding interactions. **(C)** Three-dimensional superposition of the crystallographic AQ4 conformation (cyan) and the redocked pose (green) within the EGFR ATP-binding pocket, demonstrating successful reproduction of the native binding orientation (RMSD = 1.56 Å).

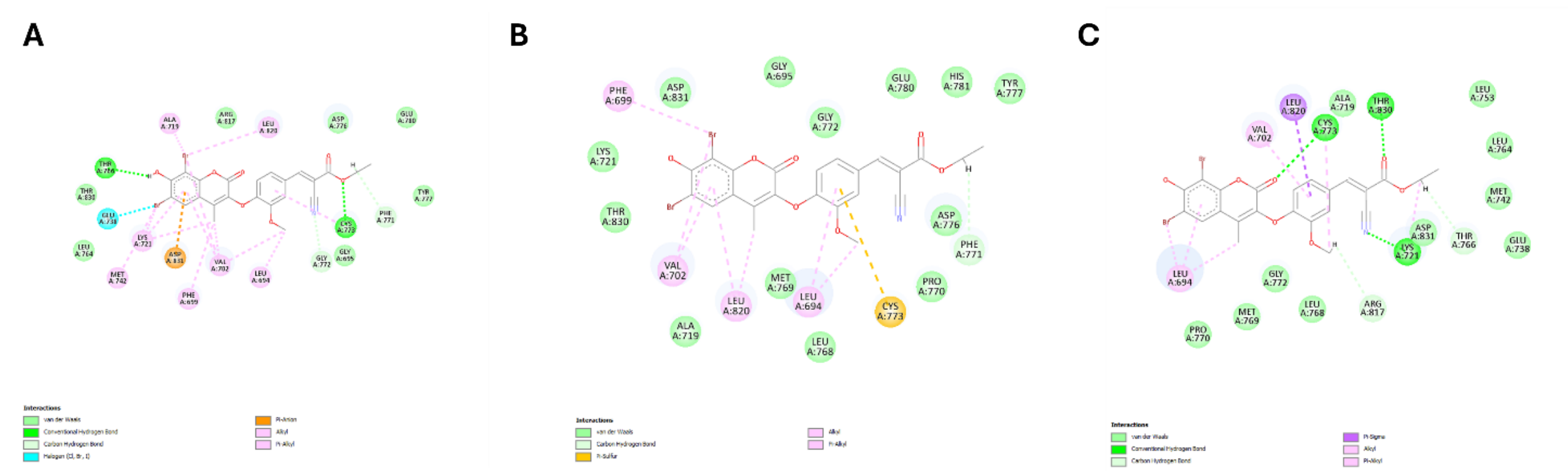

Figure S5. Two-dimensional interaction maps of alternative docking poses of compound 8 within the ATP-binding site of EGFR (PDB ID: 1M17). (**A**) Pose 2, (**B**) Pose 3, and (**C**) Pose 4, representing three alternative CNN-ranked docking solutions to the highest-ranked Pose 2 presented in Figure 4C of the main manuscript. The diagrams illustrate the predicted hydrogen-bonding, hydrophobic, electrostatic, π-mediated, halogen, and other non-covalent contacts associated with the alternative ligand orientations. Despite differences in orientation and hydrogen-bonding patterns, several contacts within the ATP-binding region recur across the poses, particularly those involving LEU694 and VAL702. Detailed docking metrics and interaction fingerprints for the four retained poses are summarized in **Table S7**.

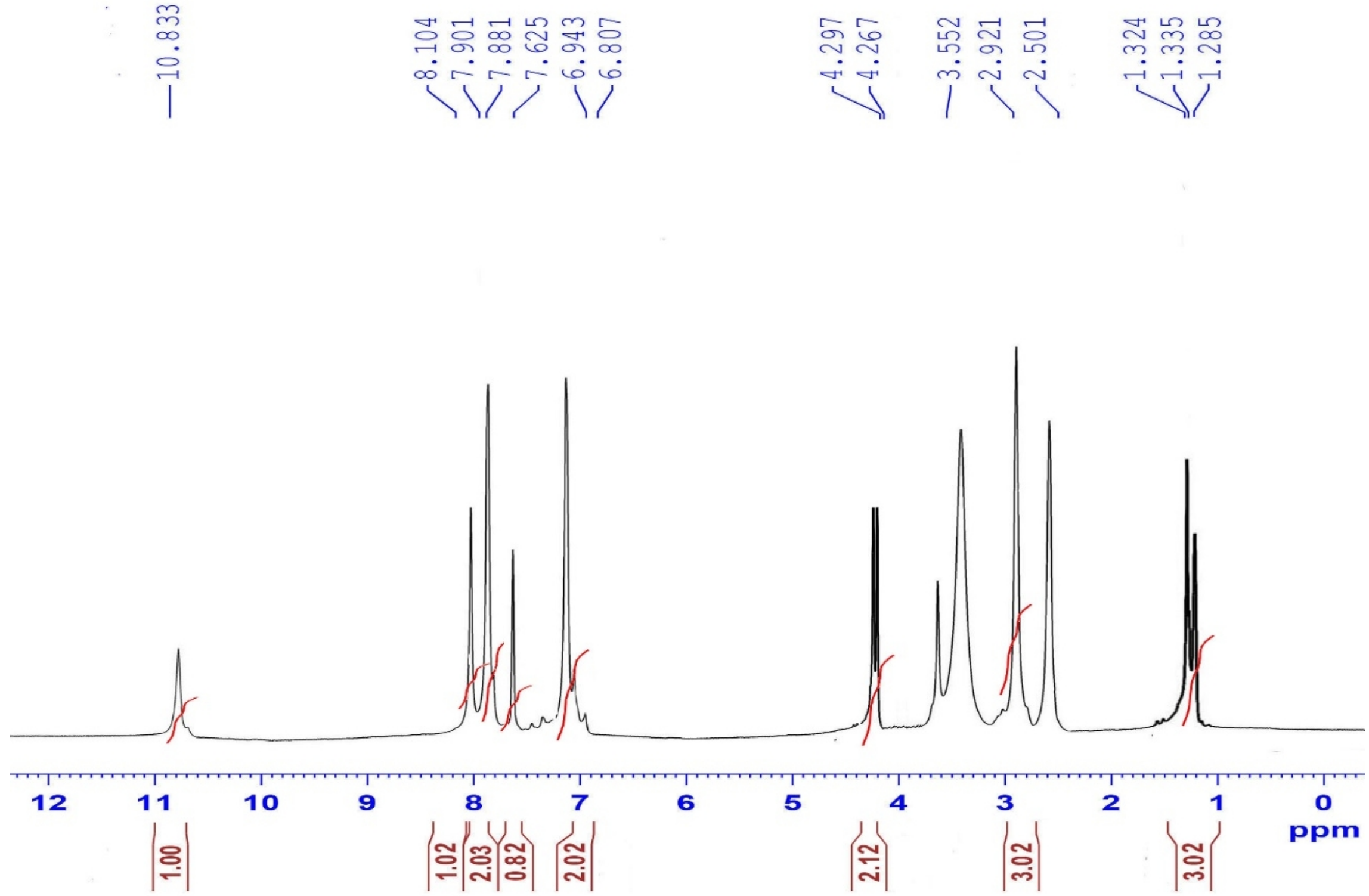

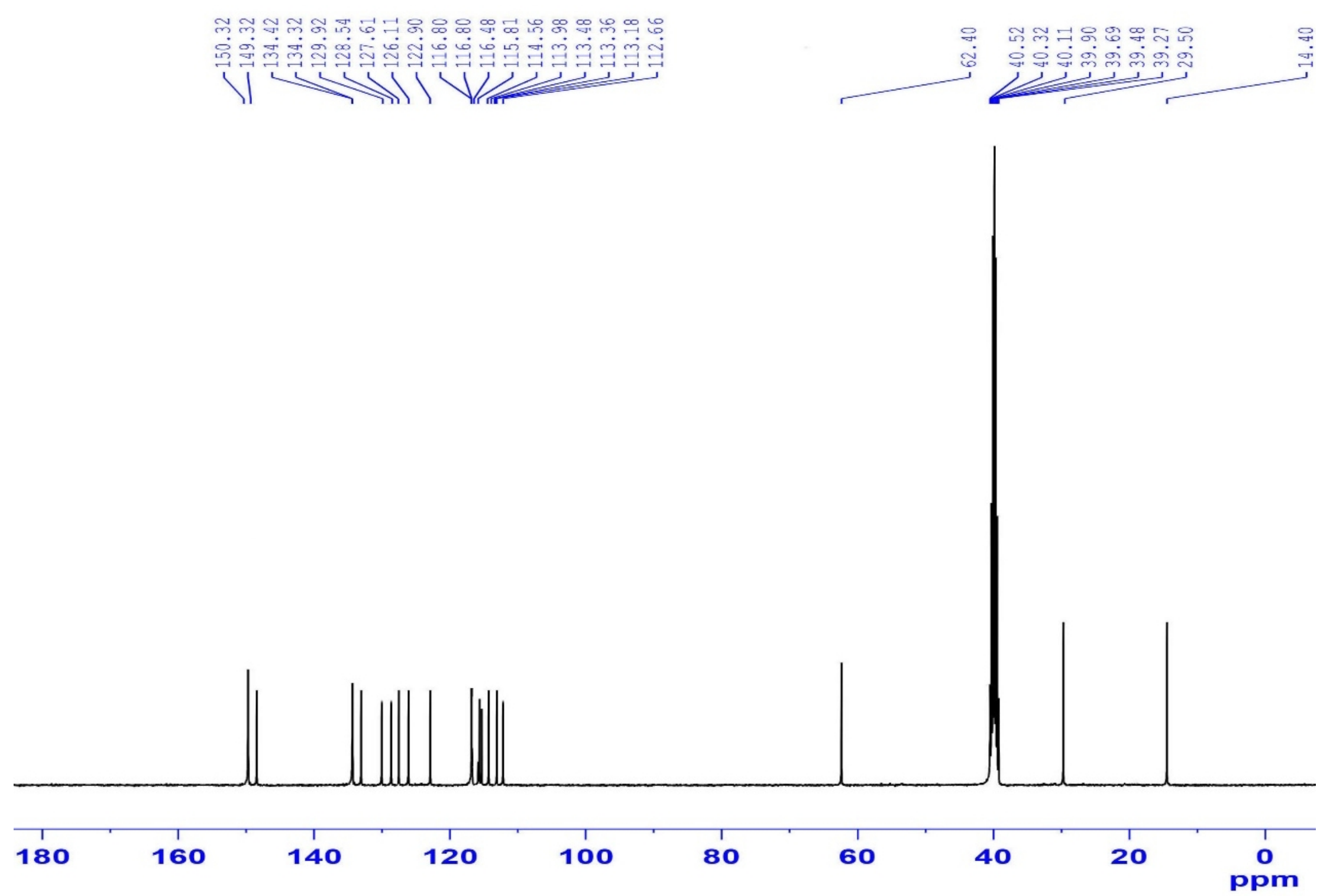

Figure S6: ^{1}H-NMR and ^{13}C-NMR spectra of compound (7)

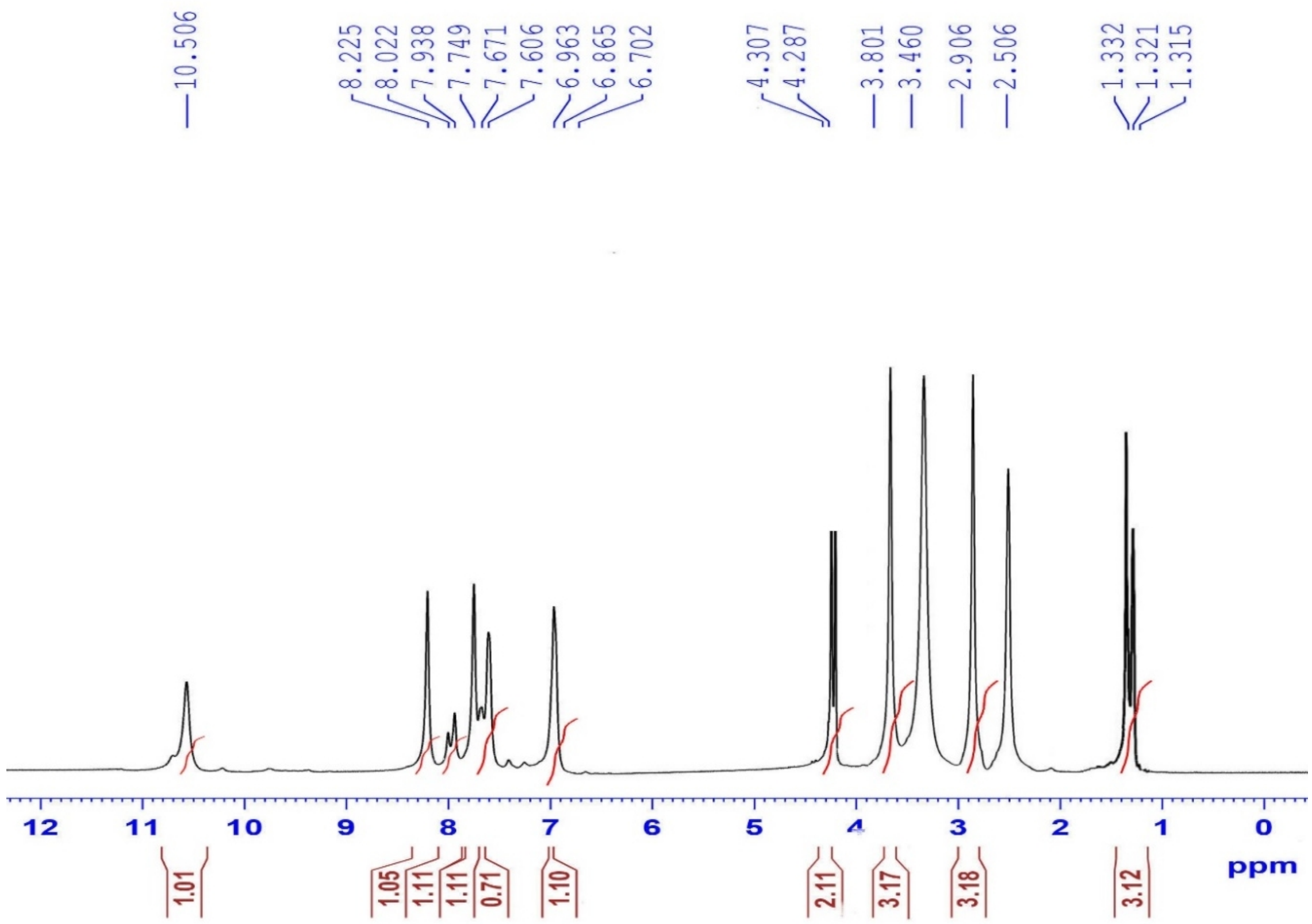

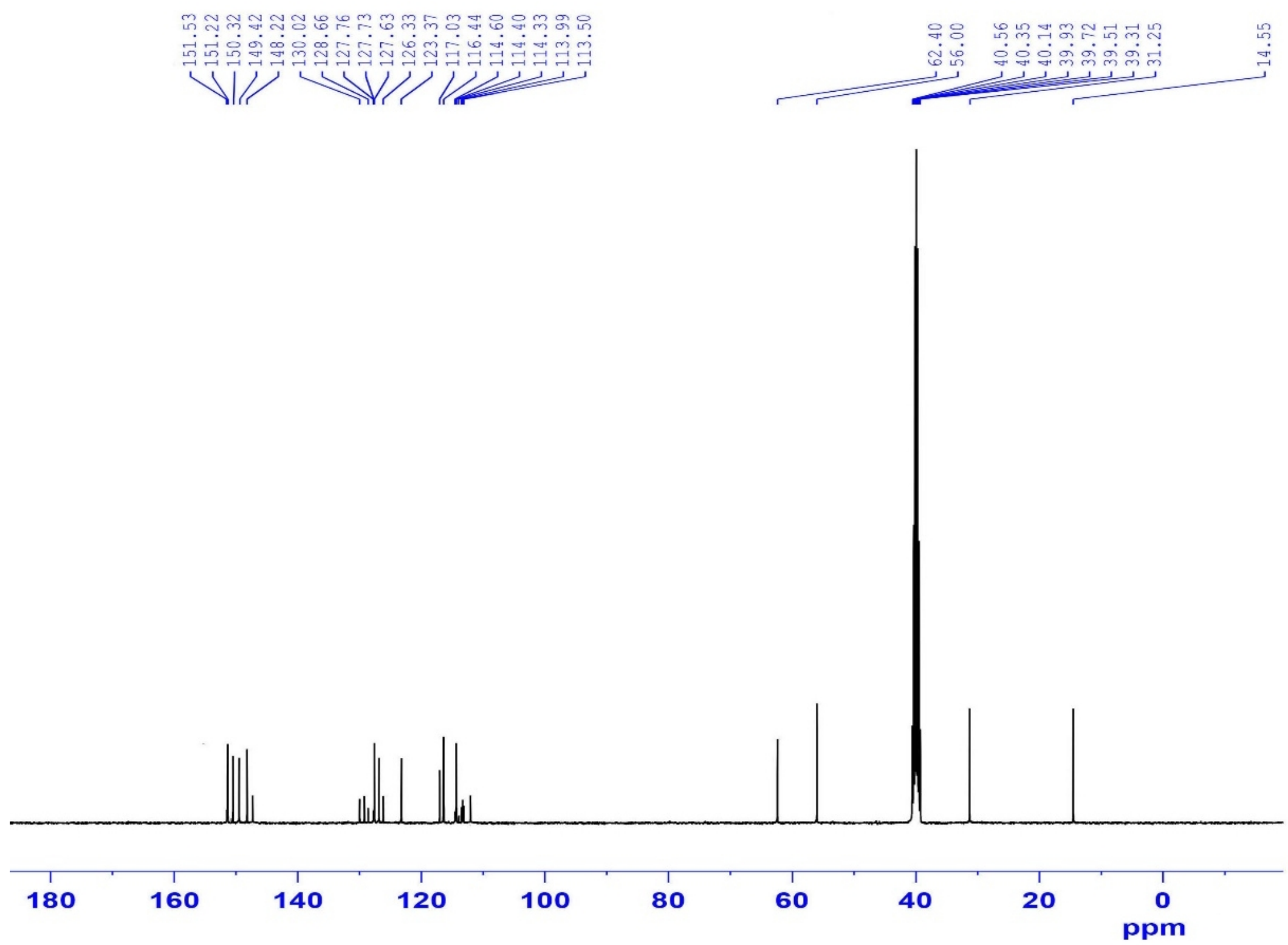

Figure S7: ^{1}H-NMR and ^{13}C-NMR spectra of compound **(8)**

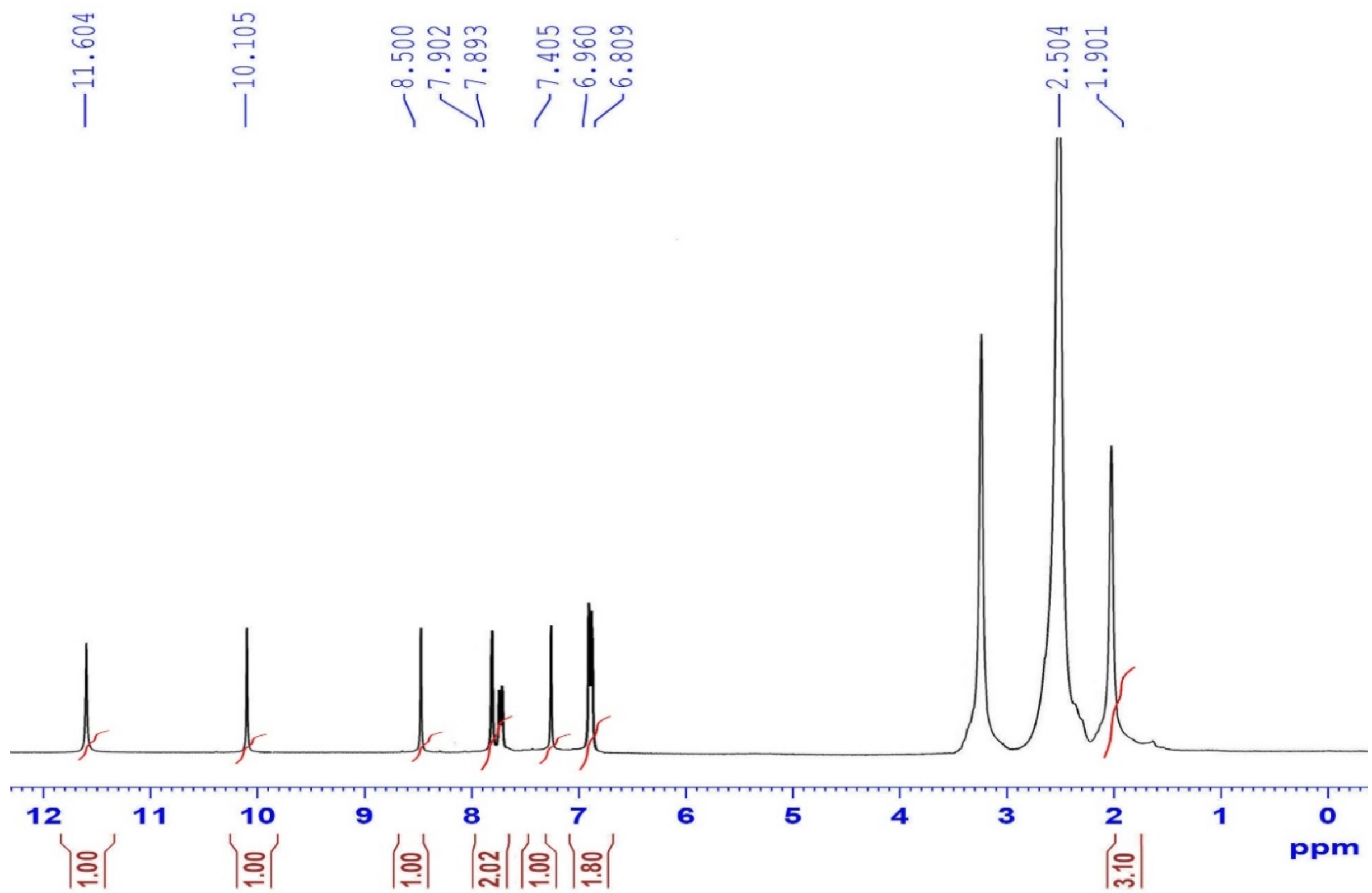

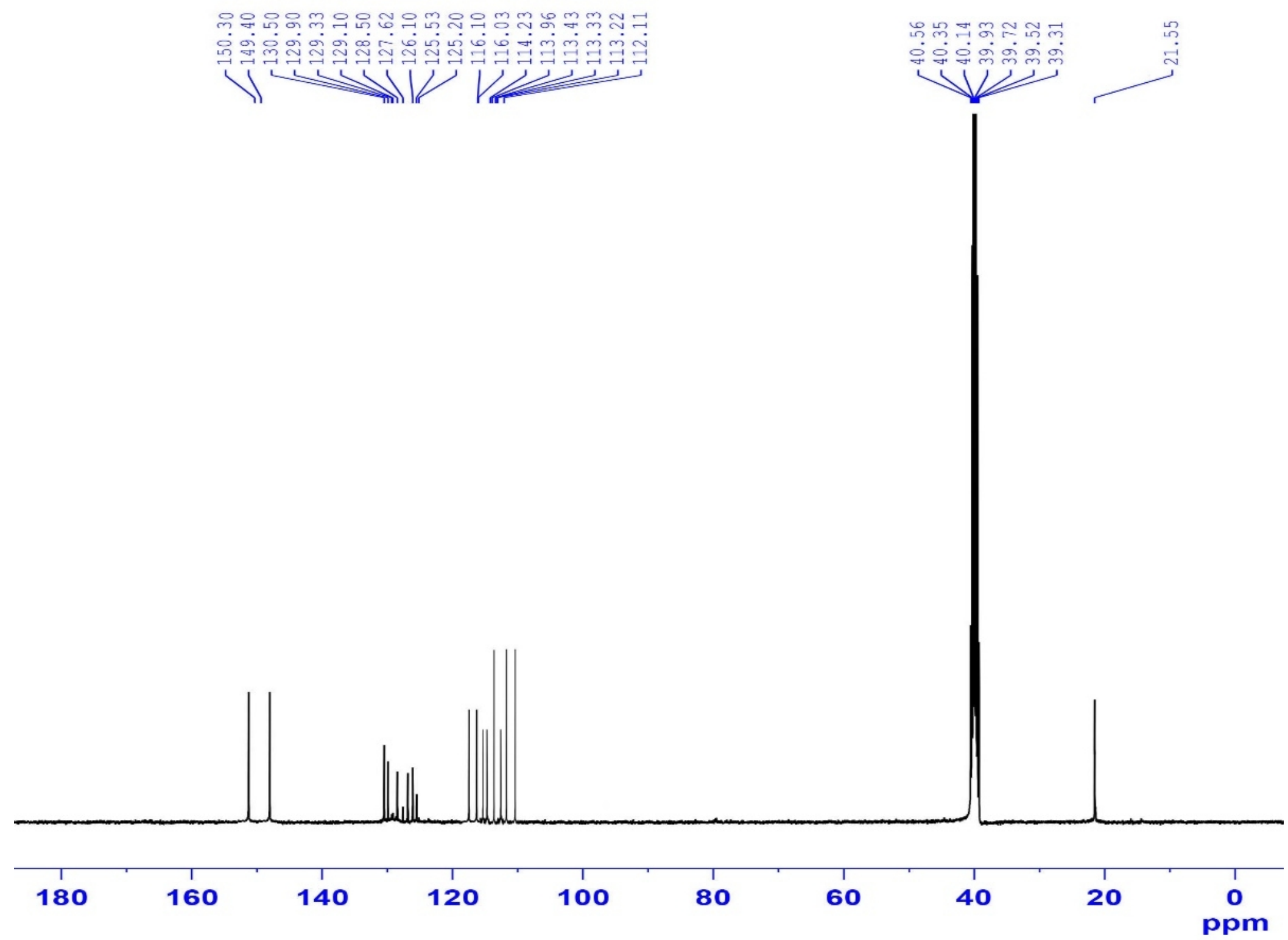

Figure S8: ^{1}H-NMR and ^{13}C-NMR spectra of compound (**9**)

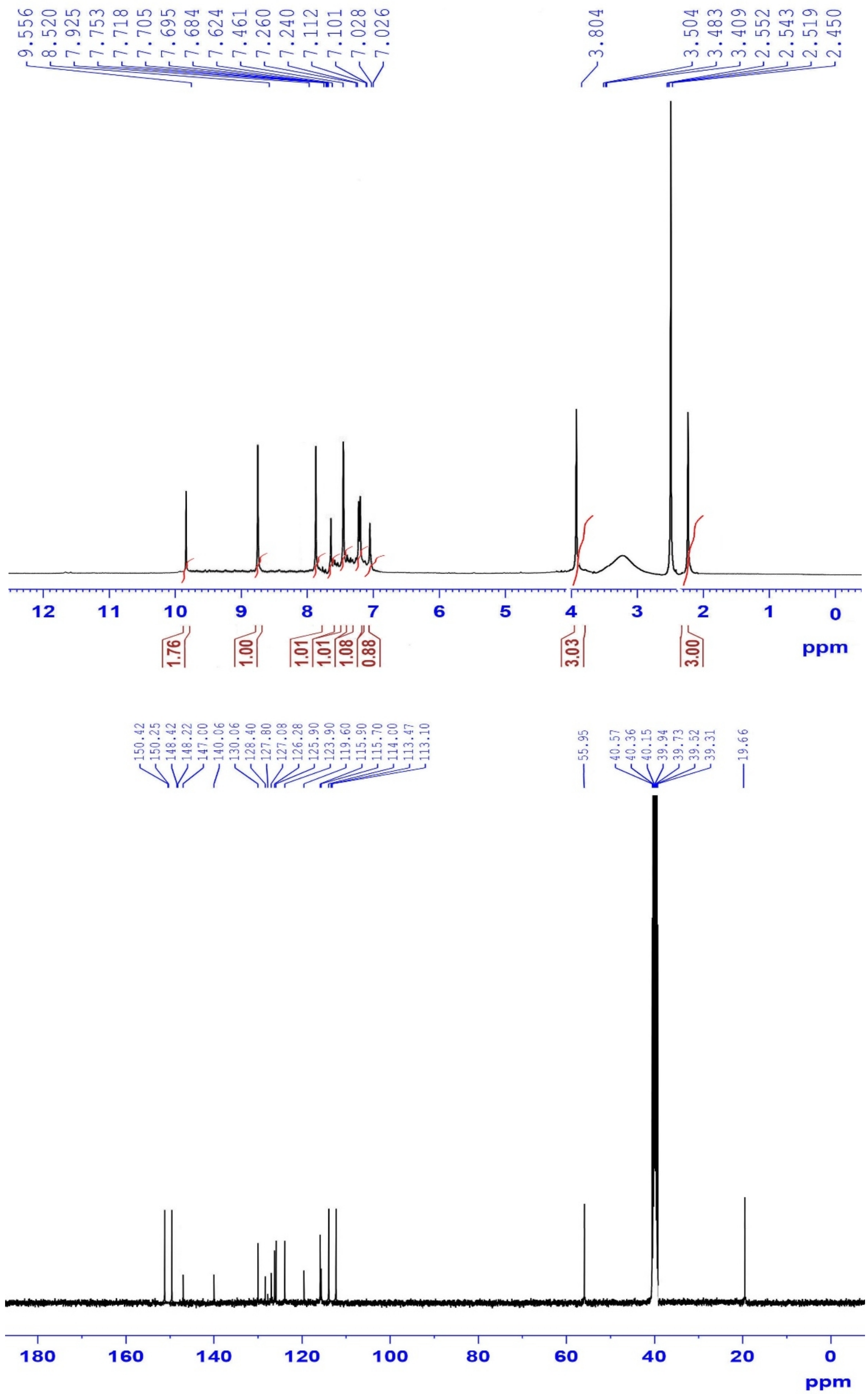

Figure S9: ^{1}H-NMR and ^{13}C-NMR spectra of compound (**10**)

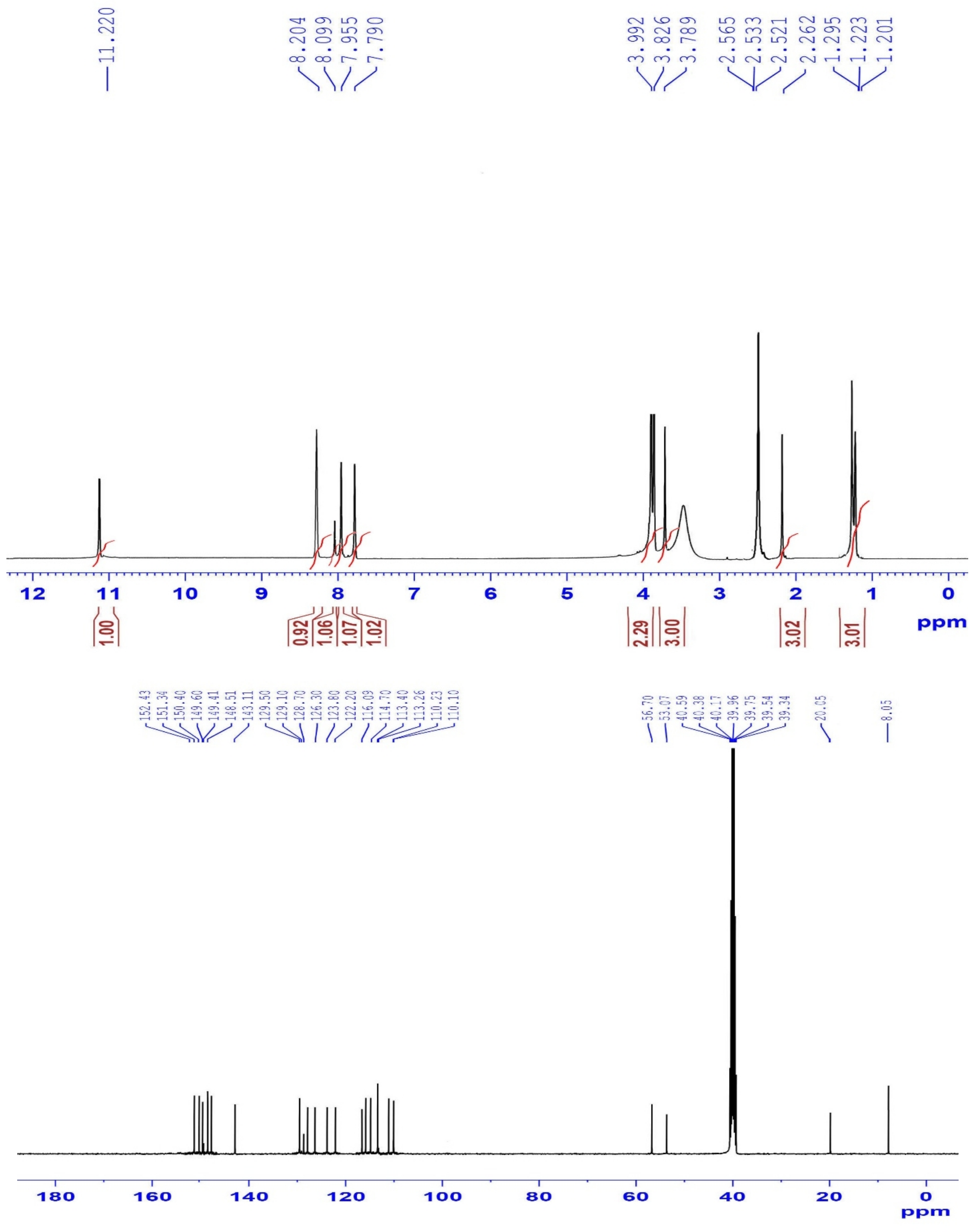

Figure S10: ^{1}H-NMR and ^{13}C-NMR spectra of compound **(11)**

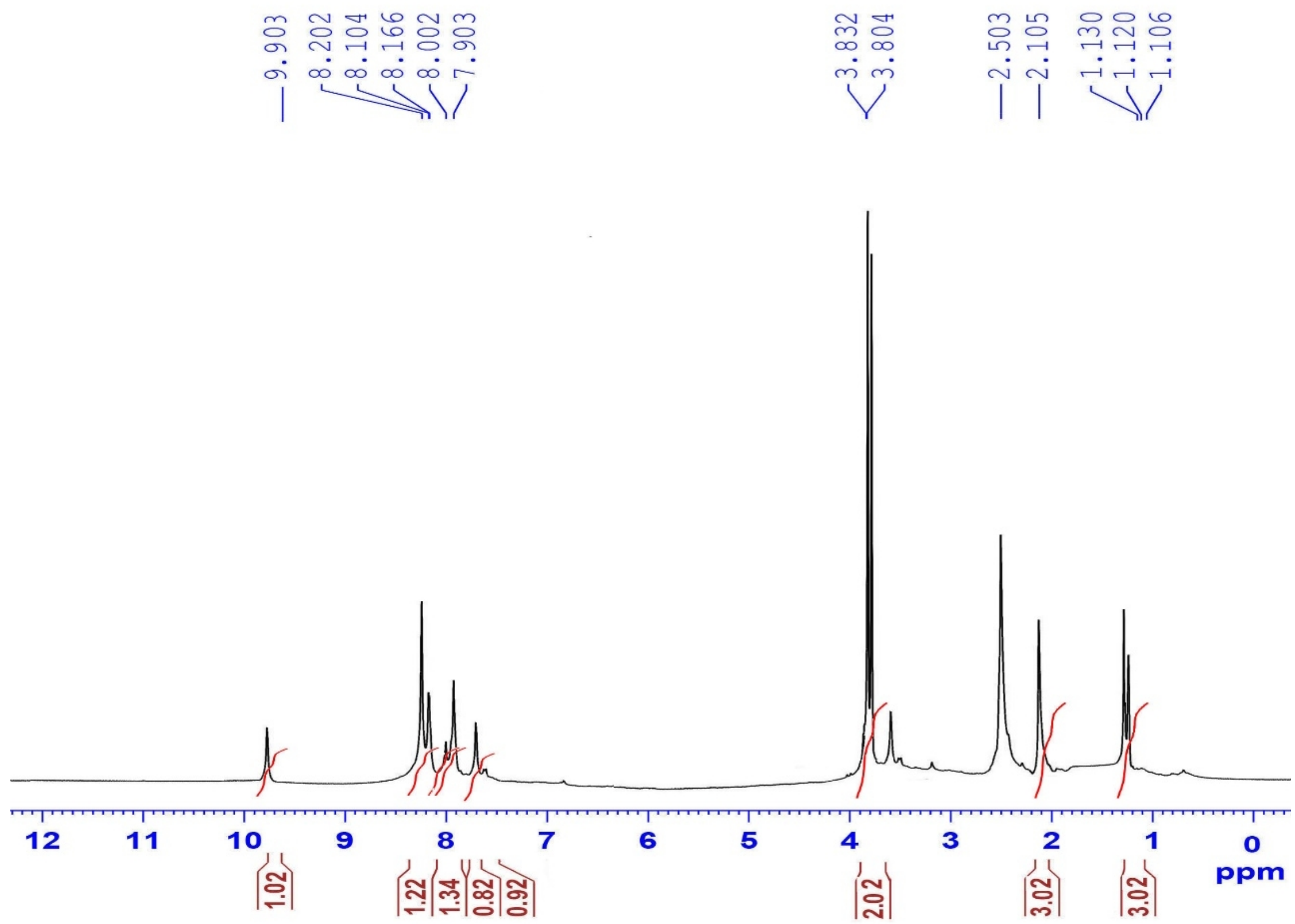

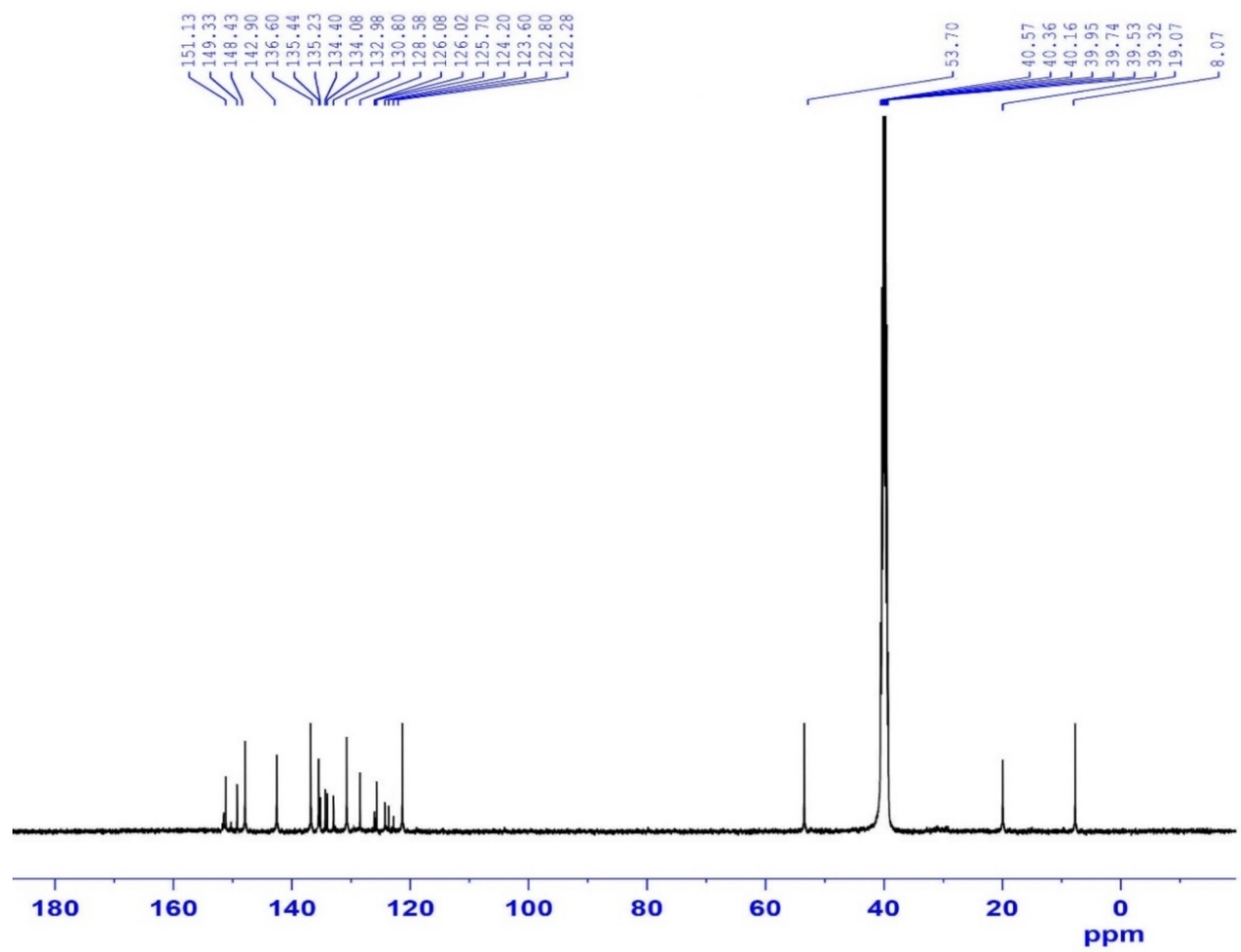

Figure S11: ^{1}H-NMR and ^{13}C-NMR spectra of compound (**12**)
